# *Pleurotus ostreatus* ethanolic extract exerts anti-cancer effects *via* PI3K/Akt/mTOR pathway modulation in DMBA-NMU induced breast cancer in female Sprague Dawley rats

**DOI:** 10.3389/fphar.2026.1766536

**Published:** 2026-04-10

**Authors:** Magdalene Eno Udobi, Israel Sunmola Afolabi, Cynthia Nwamaka Ikeji, Ebenezer Olatunde Farombi, Shalom Nwodo Chinedu

**Affiliations:** 1 Department of Biochemistry, College of Science and Technology, Covenant University, Ota, Ogun State, Nigeria; 2 Covenant Applied Informatics and Communication Africa Centre of Excellence (CApIC-ACE), Covenant University, Ota, Ogun State, Nigeria; 3 Covenant University Public Health and Wellbeing Research Cluster (CUPHWERC), Covenant University, Ota, Ogun State, Nigeria; 4 Molecular Drug Metabolism and Toxicology Laboratories, Department of Biochemistry, University of Ibadan, Ibadan, Nigeria

**Keywords:** anti-cancer, antioxidant status, breast cancer, chemoprevention, DMBA-NMU model, female rats, hormone modulation, PI3K/Akt/mTOR pathway

## Abstract

Hormone receptor–positive Breast Cancer (BC) driven by PI3K/Akt/mTOR signaling remains a major therapeutic challenge, particularly in settings where conventional chemotherapy causes severe systemic and reproductive toxicity. This study evaluated the antitumor and chemopreventive efficacy of *Pleurotus ostreatus* ethanolic extract (*PoEE*), compared with vincristine, a standard anticancer drug, in a 7,12-dimethylbenz(α)anthracene (DMBA) and N-methyl Urea (NMU) BC model using female Sprague-Dawley rats (*n* = 64). Animals were divided into eight groups receiving olive oil (control), DMBA-NMU (80 mg/kg–10 mg/kg), *PoEE* (600 mg/kg), vincristine (0.0005 mg/kg), and various combinations. After 25 weeks, mammary tissues were analyzed for antioxidant status, hormonal profiles, histopathology, and PI3K/Akt/mTOR pathway modulation using immunohistochemistry. ImageJ (NIH) and GraphPad Prism 8.0 were employed for image quantification and statistical analysis using one-way analysis of variance (ANOVA), respectively. DMBA-NMU administration induced aggressive hormone receptor–positive BC, elevating ER (∼26-fold) and PR (∼12-fold), with strong upregulation of PI3K (+21-fold), Akt (+9-fold), mTOR (+7-fold), Ras (+34-fold), MAPK (+45-fold), MDM2 (+13-fold), and PDK1 (+39-fold). Concurrently, tumor suppressors PTEN, GSK3β, and FOXO were significantly reduced by 81%, 95%, and 96%, respectively. This dysregulation was accompanied by decreased antioxidant enzyme activity (SOD −26.7%, CAT −27.2%) and hormonal imbalance (estradiol −23.9%, progesterone −11.3%). *PoEE* treatment markedly reversed these oncogenic alterations. Pre-*PoEE* treatment suppressed PI3K (−82%), Akt (−29%), mTOR (−17%), Ras (−93%), and MAPK (−83%), while restoring PTEN (+19-fold), GSK3β (+29-fold), and FOXO (+3-fold). *PoEE* enhanced estradiol (+60.9%) and progesterone (+78.9%) levels, increased SOD (+5%) and CAT (+63.6%) activities, and restored GST to 76% of control values. Post-treatment *PoEE* further reduced PI3K (−69%), Akt (−58%), and mTOR (−73%), while increasing PTEN (+10-fold), GSK3β (+7-fold), and FOXO (+27-fold), reflecting robust therapeutic potential. Vincristine moderately suppressed PI3K (−56%) and PDK1 (−88%) but elevated Akt (+19-fold) and MDM2 (+19-fold). *PoEE* and vincristine combination therapy showed selective synergy, suppressing ER (−91%), PR (−94%), Akt (−92%), and MDM2 (−94%), while increasing mTOR (+62%) and Ras (+98%). Conclusively, *PoEE* exerted potent anticancer effects through multi-target modulation of the PI3K/Akt/mTOR signaling axis. These underscore *PoEE*’s promise as a low-toxicity natural therapeutic or adjuvant for hormone receptor–positive and pathway-driven breast cancers.

## Graphical Abstract

**Figure:**
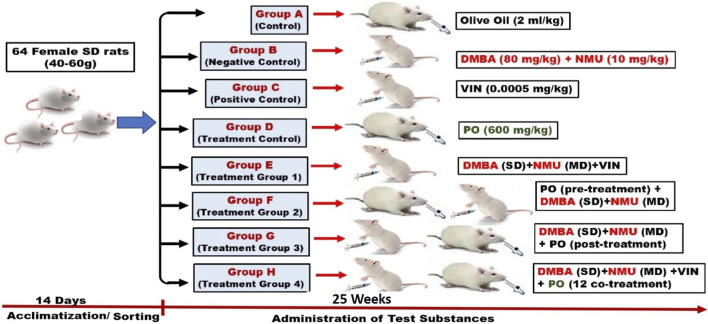

## 1 Introduction

Breast cancer (BC) remains the most prevalent malignancy among women worldwide and is a leading cause of cancer-related mortality, with a particularly disproportionate burden in low- and middle-income countries (Effiong et al., 2025a; Udobi et al., 2025). Despite major advances in detection and treatment, the disease continues to pose a global health challenge due to late diagnosis, high recurrence rates, and resistance to therapy (Xiong et al., 2025; Zafar et al., 2025). Conventional chemotherapeutic agents, such as vincristine, have improved survival outcomes, but their clinical utility is limited by severe adverse effects, including gonadotoxicity and compromised fertility, which are especially devastating for premenopausal women (Jaeck et al., 2025; Wang et al., 2025). These limitations underscore the need to identify novel therapeutic strategies that not only demonstrate efficacy against tumor progression but also minimize collateral damage to normal tissues (Effiong et al., 2025b).

A growing body of evidence suggests that natural compounds, particularly those derived from dietary sources and medicinal mushrooms, hold promise as safe and effective alternatives or adjuncts to conventional chemotherapy (Anifowose et al., 2023; Effiong et al., 2025a). *Pleurotus ostreatus*, commonly known as the oyster mushroom, is rich in bioactive compounds such as polysaccharides, triterpenoids, and phenolic acids, which have been reported to exert immunomodulatory, antioxidant, and anticancer effects (Effiong et al., 2025a; Singh et al., 2025). Importantly, mushrooms represent a unique source of multifunctional agents capable of targeting diverse carcinogenic processes, including oxidative stress, chronic inflammation, and dysregulated signaling cascades (Sreedharan et al., 2025). Given their natural origin and low toxicity, mushroom-derived extracts are attractive candidates for chemoprevention and therapy (Ikram et al., 2025), particularly in hormone receptor-negative breast cancers, which remain clinically intractable.

The Phosphatidylinositol 3-Kinase/Protein Kinase B/Mammalian Target of Rapamycin (PI3K/Akt/mTOR) pathway is a key oncogenic pathway frequently hyperactivated in breast cancer (Effiong et al., 2025a; Effiong et al., 2025c; Hassan and Aubel, 2025). As a central regulator of cell growth, proliferation, survival, and metabolism, its dysregulation is a hallmark of the disease, often leading to therapy resistance (Garg et al., 2025). The aberrant activation of this pathway, driven by the loss of tumour suppressors like Phosphatase and Tensin Homolog (deleted on chromosome 10) (PTEN) and the overexpression of Phosphatidylinositol 3-Kinase (PI3K) and Protein Kinase B (Akt), fosters a pro-survival and proliferative phenotype in cancer cells. Conversely, the activation of downstream tumour suppressors such as Forkhead box O (FOXO) can promote cell cycle arrest and apoptosis (Bergez-Hernández et al., 2024). Inhibition of PI3K/Akt/mTOR signaling has therefore emerged as a promising therapeutic strategy; however, available pharmacological inhibitors are constrained by toxicity, resistance, and limited efficacy in clinical settings (Qiang et al., 2025; Versari et al., 2025). Natural agents capable of modulating this pathway may offer a safer and more effective alternative for breast cancer treatment.

Previous studies have provided some experimental evidence for the anticancer properties of *P. ostreatus* (Jacq.) P. Kumm. (oyster mushroom), demonstrating its ability to induce apoptosis and cell cycle arrest in various cancer cell lines, including those of leukemia and breast cancer (Ebrahimi et al., 2018; Haque and Islam, 2019; Jayaprakash et al., 2024; Moyen Uddin Pk et al., 2024; Shi et al., 2013). Research has shown that extracts and bioactive compounds from this mushroom can suppress key enzymes like Matrix Metalloproteinase-2 (MMP-2) and Matrix Metalloproteinase-9 (MMP-9) (Jayaprakash et al., 2024). Furthermore, *P. ostreatus* has been consistently shown to possess potent antioxidant and anti-inflammatory properties, which are critical for neutralizing oxidative stress and chronic inflammation, two key drivers of carcinogenesis (Aguilar Uscanga et al., 2020; Elhusseiny et al., 2021; Mishra et al., 2022). *In vivo* studies have also demonstrated its ability to modulate oxidant/antioxidant status and reduce tumor growth in animal models, supporting its potential as a chemopreventive agent (Krishnamoorthy and Sankaran, 2016; Maiti et al., 2011). Nevertheless, a comprehensive *in-vivo* investigation linking *P. ostreatus*–mediated tumor suppression to PI3K/Akt/mTOR pathway modulation remains absent. Moreover, its interaction with conventional chemotherapeutics and influence on hormonal and antioxidant homeostasis have not been systematically explored.

The multistep nature of breast cancer characterized by sequential initiation, promotion, and progression events driven by cumulative genetic, epigenetic, and hormonal alterations necessitates a wholistic approach in mimicking this breast carcinogenic process *in-vivo*. Although single-agent models using 7,12-dimethylbenz [a]anthracene (DMBA) or N-methyl-N-nitrosourea (NMU) are standard, they often represent isolated aspects of carcinogenesis with DMBA requiring metabolic activation and typically inducing H-ras mutations, and NMU acting as a direct alkylating agent. In this study, a dual-carcinogen approach was employed to better recapitulate the multi-stage nature of human breast cancer. DMBA utilized as an initiator and NMU as a promoter/progressor, the novel model mimics the transition from early genetic insult to aggressive phenotypic progression. This sequential administration enhances the reliability of tumor induction and better reflects the molecular heterogeneity seen in human breast cancers, particularly through the sustained activation of the PI3K/Akt/mTOR signaling axis, which is frequently dysregulated in advanced clinical cases.

Therefore, this study addresses these critical gaps by evaluating the antitumor and chemopreventive effects of *PoEE* alone and in combination with vincristine, in a 7,12-dimethylbenz(α)anthracene (DMBA) and N-methyl Urea (NMU) (DMBA–NMU)–induced breast cancer model in female Sprague-Dawley (SD) rats. It applies a novel dual-carcinogen DMBA-NMU model that closely mimics the hormonal and molecular progression of human BC, by integrating histopathological, biochemical, and immunohistochemical analyses, the study elucidates how *PoEE* modulates PI3K/Akt/mTOR signaling, redox balance, and hormonal regulation to achieve tumor suppression. The novelty of this work further lies in its mechanistic *in vivo* demonstration that a naturally derived mushroom extract can restore tumor suppressor activity, attenuate oncogenic signaling particularly PI3K/Akt/mTOR pathway, and mitigate chemotherapy-related toxicity, providing a translational framework for developing safe, multi-targeted therapies for hormone-driven breast cancer.

## 2 Materials and methods

### 2.1 Sample collection

Twenty-five kilograms of fresh oyster mushrooms (*P*. *ostreatus*) were obtained from a certified local farm in Agbara, Ogun State, Nigeria, and authenticated by the Department of Botany, University of Ibadan. The species name was validated using Index Fungorum (accessed January 2024). The mushrooms were thoroughly washed to remove debris, wiped with a sterile towel to eliminate surface moisture, and oven-dried at 55 °C–65 °C until completely dehydrated (Effiong et al., 2024a; Effiong et al., 2025a). The dried samples were ground into fine powder using a laboratory blender, yielding 2.5 kg of mushroom powder, which was stored in airtight containers at room temperature prior to extraction.

Following the modified method of Zhang et al. (2020), 20 g of the powdered sample was extracted with 300 mL of absolute ethanol and continuously stirred for 24 h at room temperature. The mixture was then filtered twice using Whatman No. 1 filter paper, and the filtrate was concentrated under reduced pressure with a rotary evaporator to remove the solvent. The resulting *P*. *ostreatus* ethanolic extract (*PoEE*) was weighed to determine percentage yield and stored at 4 °C for subsequent biochemical and molecular analyses. Preliminary phytochemical screening and Gas Chromatography Mass Spectrometry (GCMS) of *PoEE* (Effiong et al., 2024a; Effiong et al., 2024b) revealed the presence of phenolic metabolites, flavonoids, polysaccharides, terpenoids, and alkaloids, which are known bioactive constituents contributing to the extract’s antioxidant and anticancer properties.

### 2.2 Chemical characterization of the components in *Pleurotus ostreatus* ethanolic extract (*PoEE*)

#### 2.2.1 Gas chromatography-mass spectrometry (GC-MS) analysis of *Pleurotus ostreatus* ethanolic extract

*Pleurotus ostreatus* ethanolic extract was subjected to GC-MS analysis on a GCMS-QP2010SE SHIMADZU JAPAN with a fused Optima-5MS capillary column that measured 30 m in length, 0.25 mm in diameter, and 0.25 μm in film thickness (Effiong et al., 2024a). Pure helium (1.56 mL min^−1^ flow rate and 37 cm s^−1^ linear velocity), injector temperature (200 °C), column oven temperature (60 °C initially, then increased to 160 °C and later to 250 °C at 10 °C min^−1^ with 2 min/increment hold time), and injection volume and split ratio (0.5 μL and 1:1, respectively) were the GC conditions. The MS settings included an ion source at 230 °C, an interface temperature of 250 °C, a solvent delay of 4.5 min, and a scan range of 50–700 amu. By comparing the retention time, mass spectrum data, and fragmentation pattern of the extracts with reputable libraries (National Institute of Standards and Technology (NIST) and Wiley libraries), unknown substances were found (Iheagwam et al., 2019a; Iheagwam et al., 2019b).

#### 2.2.2 HPLC analysis of *Pleurotus ostreatus* ethanolic extract

The HPLC identification and characterization of flavonoids, and phenols in the ethanol extract of *P. ostreatus* was carried out using the method described by (Afolabi et al., 2023). An aliquot of the sample extracts (0.1 g) was combined with 10 mL of 70% methanol in a closed test tube and left to stand for 1–2 h. The extracted material was then decanted, centrifuged using a chilled centrifuge (model: CR21G, serial number: S2025709), and filtered through a micron filter into a 5 mL sample container. The sample filtrate was used to analyze the saponins, phenolic and flavonoid components in the extracts of *P. ostreatus* using HPLC (Afolabi et al., 2023).

##### 2.2.2.1 HPLC analysis for the phenol fractions of *PoEE*

The extracted phenolic samples (40 µL) were injected into the HPLC (model: Agilent LC-8518) running with acetonitrile/water/acetic acid (19:80:1) mobile phase, at 272 nm wavelength, and a run time of 25 min. To analyze flavonoids in extracts, N2000 chromatography software was used with a high-sensitivity LC-8518 diode array (DA) detector, a column (150 mm × 4.6 mm) set at 35 °C, and a low-pressure gradient and solvent delivery LC-8518 pump with a high-pressure switching valve (Afolabi et al., 2023).

##### 2.2.2.2 HPLC analysis for the flavonoids fractions of *PoEE*

The extracted flavonoid samples (40 µL) were injected into the HPLC (model: Agilent LC-8518) running with acetonitrile, water and formic acid (25:74:1) mobile phase, 210 nm wavelength, and a run period of 25 min. To analyze flavonoids in extracts, N2000 chromatography software was used with a high-sensitivity LC-8518 diode array (DA) detector, a column (150 mm × 4.6 mm) set at 40 °C, and a low-pressure gradient and solvent delivery LC-8518 pump with a high-pressure switching valve (Afolabi et al., 2023).

### 2.3 Ethical declaration

The research team obtained approval from the management of Covenant University to carry out the study. The Covenant University Health Research Ethics Committee (CHREC) granted ethical approval for this study under the reference number CU/HREC/EME/204/23.

### 2.4 Animal housing

Sixty-four healthy female Sprague-Dawley rats (4–6 weeks old, 40–60 g) were obtained and acclimatized for 2 weeks prior to the experiment. The animals were housed in well-ventilated polypropylene cages (8 rats per cage) under standard laboratory conditions: 12-h light/12-h dark photoperiod, temperature of 22 °C ± 2 °C, and relative humidity of 50%–60%. Rats were provided with standard pellet diet and clean water *ad libitum*. All experimental procedures adhered to the Guide for the Care and Use of Laboratory Animals (MacArthur Clark and Sun, 2020) and were approved by the Covenant University Health Research Ethics Committee (CHREC) -. CU/HREC/EME/204/23. Efforts were made to minimize pain and distress throughout the study.

### 2.5 Experimental design

**FIGURE 1:**
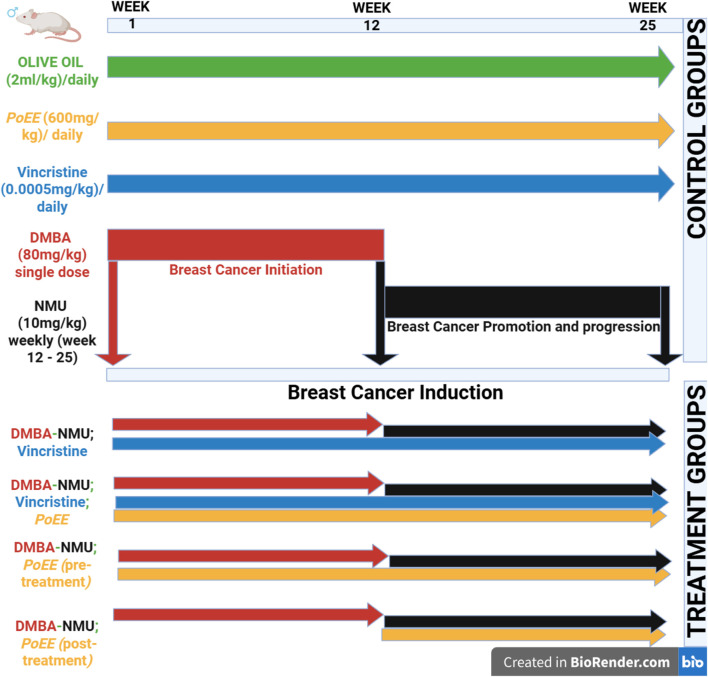
Experimental design for the evaluation of the effects of *PoEE* on DMBA-NMU induced breast cancer in Female Sprague Dawley Rats. Abbreviations: MD, Multiple doses; SD, Single Doses; VIN, Vincristine; DMBA, 7,12-dimethylbenz [a]anthracene; NMU, N-methyl-N-nitrosourea.

The experimental design comprised eight groups (*n* = 8 per group) (Figure 1; Table 1), with breast carcinogenesis induced using a dual-carcinogen model consisting of 7,12-dimethylbenz [a]anthracene (DMBA; 80 mg·kg^−1^, single oral dose) as initiator and N-methyl-N-nitrosourea (NMU; 10 mg·kg^−1^, intraperitoneal, weekly from week 12 post-DMBA) as promoter and progressor (Figure 1; Table 1). Doses were selected based on validated mammary tumor induction protocols: DMBA (80 mg·kg^−1^) from Dania et al. (2024), NMU (10 mg·kg^−1^) and vincristine (0.0005 mg·kg^−1^) from Adefisan et al. (2022), and *P*. *ostreatus* ethanolic extract (*PoEE*; 600 mg·kg^−1^) from Krishnamoorthy and Sankaran (2016). The *PoEE* was administered orally daily, a regimen chosen for its demonstrated chemopreventive efficacy and safety, as well as to provide sustained systemic exposure while minimizing handling stress. Vincristine was administered at a dosage of 0.0005 mg·kg^−1^, intraperitoneal, daily, allowing a direct and balanced comparison between therapeutic and natural-product regimens.

**TABLE 1:** Experimental groups and treatment regimens.

Group	Group name	Treatment description
A	Control	Olive oil only (2 mL·kg^−1^, oral, daily)
B	Negative control	DMBA (80 mg·kg^−1^, single oral dose) + NMU (10 mg·kg^−1^, intraperitoneal, weekly)
C	Positive control	*PoEE* (600 mg·kg^−1^, oral, daily)
D	Treatment control	Vincristine (0.0005 mg·kg^−1^, intraperitoneal, daily)
E	Treatment group 1	DMBA (80 mg·kg^−1^, single oral dose) + NMU (10 mg·kg^−1^, weekly) + vincristine (0.0005 mg·kg^−1^, daily)
F	Treatment group 2 (pre-treatment)	*PoEE* (600 mg·kg^−1^, oral, daily) administered before DMBA (80 mg·kg^−1^, single oral dose) + NMU (10 mg·kg^−1^, weekly)
G	Treatment group 3 (post-treatment)	DMBA (80 mg·kg^−1^, single oral dose) + NMU (10 mg·kg^−1^, weekly) followed by PoEE (600 mg·kg^−1^, oral, daily)
H	Treatment group 4 (combination)	DMBA (80 mg·kg^−1^, single oral dose) + NMU (10 mg·kg^−1^, weekly) + vincristine (0.0005 mg·kg^−1^, daily) + *PoEE* (600 mg·kg^−1^, oral, daily)

Using standard allometric scaling (Km = 6 for rats, 37 for humans), the corresponding human-equivalent dose of *PoEE* (600 mg·kg^−1^) is approximately 97 mg·kg^−1^ (≈5.8 g for a 60 kg adult), which lies within the tolerable range for oral mushroom extract consumption in humans.

$$\text{HED }\left(\text{mg}/\text{kg}\right)=\text{Animal Dose }\left(\text{mg}/\text{kg}\right)\times \left(\frac{\text{Rat}\;K_{m}\;\left(6\right)}{\text{Human}\;K_{m}\;\left(37\right)}\right)$$

Treatment groups included olive oil (2 mL·kg^−1^, daily) as control; *PoEE* alone; vincristine alone; and various *PoEE*–vincristine combinations. *PoEE* was administered both before (preventive phase) and after (therapeutic phase) DMBA–NMU exposure to assess its chemo-preventive and antitumor efficacy across initiation, promotion, and progression stages. The study lasted 25 weeks, after which all animals were anesthetized and humanely euthanized for tissue collection and analysis, in compliance with institutional ethical approvals (protocol no. CU/HREC/EME/204/23).

### 2.6 Clinical observation and sacrifice of experimental animals

During the administration period, the animals were observed for the development of breast tumors, changes in body weight, food consumption, and water consumption. The body weight of each rat was recorded in the course of the study. After the experimental duration, the animals were fasted overnight for 16 h and sacrificed by cardiac puncture under mild euthanasia using a ketamine/xylazine mixture (10:1 v/v). Ketamine at 30 mg/kg body weight (Ketamax, Troikaa Pharmaceuticals Ltd., India) and xylazine at 10 mg/kg body weight (Xylaxin, Indian Immunologicals Limited, India) was used. The blood was collected into non-heparinized tubes and EDTA bottles. The serum was then separated by centrifugation of the clotted blood at 4,000 g for 10 min with a table centrifuge.

### 2.7 Breast tissue preparation

Breast tissues were harvested, rinsed in ice-cold 1.15% potassium chloride (KCl) solution, blotted, weighed, and portions were fixed for histology. The remaining tissues were homogenized in 0.1 M phosphate buffer (pH 7.4) using a WHEATON Power Homogenizer and Overhead Stirrer, Complete Unit, 120 VAC (Wheaton, United States). The homogenates were centrifuged at 10,000 rpm for 15 min at 4 °C in a refrigerated centrifuge (Thermo Scientific Sorvall, Product Code: SM101446-16, United States) to obtain the post-mitochondrial fraction. Supernatants were collected for biochemical analyses, while tissues for histopathology were rinsed in 10% formalin and stored until processing.

### 2.8 Determination of total body and relative organ weights

The total body weight of each rat was determined using digital chemical balance before and after the experimental period (as initial and final body weights, respectively), and the mean body weight for each group was calculated. Weight changes were expressed as percentage weight increase and percentage growth rate where:

Percentage weight increase was calculated from the formula:

$$\frac{W_{y}-W_{x}}{W_{x}}\times 100$$

Where W_x_ = Initial mean body weight; W_y_ = Final mean body weight.

### 2.9 Biochemical assessments

The antioxidant assays carried out include determination of Superoxide Dismutase (SOD) activity, Catalase (CAT) activity, Glutathione S-Transferase (GST) activity, Glutathione Peroxidase (GPx), reduced glutathione (GSH) levels, and total thiol (TSH) levels in the homogenates of the breast tissues. Total protein concentration in the breast tissue homogenates were estimated according to the method described by Bradford, (1976), Kielkopf et al. (2020). The activity of SOD was determined by the method of Misra and Fridovich (1972). Catalase activity was determined using Claiborne (1985). Glutathione S-transferase activity was determined according to Habig et al. (1974). The method of Beutler et al. (1963) was followed in estimating the level of reduced glutathione (GSH). Glutathione peroxidase (GPX) activity was measured according to the procedure of Lawrence et al. (1974), with some modifications. Total Thiol levels was measured according to the procedure of Owumi et al. (2023), Ellman (1959).

#### 2.9.1 Determination of tissue protein concentration

The breast tissue protein content was determined using the Bradford method, with Bovine Serum Albumin (BSA) as the standard reference according to the method described by Bradford (1976), Kielkopf et al. (2020). This assay is based on the alteration of the Coomassie brilliant blue (CBB) G-250 dye’s ability to absorb light when in contact with the protein in the tissues. The absorption maxima of the dye shifts to 595 nm upon the introduction of protein in the tissues following its initial light absorbance at wavelength of 465 nm on the initial preparation of the dye in an acidic solution (Kielkopf et al., 2020). A standard solution of BSA containing protein concentrations from 0 to 50 µg protein/mL was dissolved in distilled water. Additionally, 1.6 mL of Coomassie brilliant blue dye was added to the individual 0.4 mL BSA solutions, then left to stand at room temperature for 5 min after which the optical densities were plotted against the BSA content following the determination of the solution’s absorbance at 595 nm (Moreira, 2022; Soares et al., 2023). To obtain protein values within the standard curve range in the breast post-mitochondria fraction, the breast were diluted in a ratio of 1:1,000.0.3 mL of the diluted samples were pipetted in a glass cuvette, then 1.47 mL of the Bradford reagent was added and left to sit for 5 min at room temperature. The absorbance was monitored at 595 nm (Moreira, 2022; Soares et al., 2023). The total protein levels in the tissue homogenates were extrapolated from the standard curve.

#### 2.9.2 Determination of superoxide dismutase activity

The activity of SOD was determined by the method of Misra and Fridovich (1972). The ability of SOD to inhibit the auto-oxidation of epinephrine at pH 10.2 makes this reaction a basis for a simple assay for this dismutase. Superoxide radicals cause the oxidation of epinephrine to adrenochrome, and the yield of adrenochrome produced per superoxide radicals introduced increases with the increasing pH and concentration of epinephrine. An aliquot (50 µL) of the sample was added to 2.5 mL of 0.05 M carbonate buffer (pH 10.2) and 0.3 mL of epinephrine in a cuvette, mixed by inversion and change in absorbance monitored every 30 s for 2.5 min at 480 nm. The reference cuvette was the same as the samples with water replacing them.

$$\%\text{ inhibition}=100\;–\;(100\;\times \frac{\left.\text{Increase}\;\text{in}\;\text{absorbance}\;\text{per}\min\text{for}\;\text{sample}\right)}{\text{Increase}\;\text{in}\;\text{absorbance}\;\text{per}\;\min\;\text{for}\;\text{blank}}$$

1 unit of SOD activity was given as the amount of SOD necessary to cause 50% inhibition of the auto-oxidation of epinephrine.

#### 2.9.3 Determination of catalase activity

Catalase activity was determined using the method described by Claiborne (1985). The method is based on the reduction in absorbance observed at 240 nm as catalase splits hydrogen peroxide. Despite the fact that hydrogen peroxide has no absorbance maximum at this wavelength, its absorbance correlates well enough with concentration to allow its use for a quantitative assay. An extinction coefficient of 0.0436 mM^−1^cm^−1^ was used (Hadwan, 2018). Hydrogen peroxide (2.95 mL of 19 mM solution) was pipetted into a 1 cm quartz cuvette, and 50 µL of sample was added. The mixture was rapidly inverted to mix and placed in a spectrophotometer. Change in absorbance was read at 240 nm every minute for 5 min.

$$\text{Catalase activity}=\frac{\Delta A_{240}/\min\times \text{reaction}\;\text{volume}\times \text{dilution}\;\text{factor}}{0.0436\times \text{sample}\;\text{volume}\times \text{mg}\;\text{protein}/\text{mL}}$$

$$=µ\text{mole }H_{2}O_{2}/\min/\text{mg protein}$$

#### 2.9.4 Estimation of glutathione s-transferase activity

Glutathione S-transferase (GST) activity was determined according to Habig et al. (1974). The assay is based on the principle that all known GST isozyme demonstrate a relatively high activity with 1-chloro-2,4-dinitrobenzene (CDNB) as the second substrate. When CDNB is conjugated to reduced glutathione, its absorption maximum shifts to a longer wavelength, and the absorption increase at the new wavelength of 340 nm directly measures the enzymatic reaction. The medium for the estimation was prepared and the reaction was allowed to run for 3 min with readings taken every 60 s against the blank at 340 nm.

The extinction coefficient of CDNB at 340 nm = 9.6 mM^−1^cm^−1^

$$\text{GSH }S-\text{transferase activity}=\frac{\Delta A_{340}/\min\times \text{reaction}\;\text{volume}\times \text{dilution}\;\text{factor}}{9.6\times \text{sample}\;\text{volume}\times \text{mg}\;\text{protein}/\text{mL}}$$

=µmole/⁡min⁡/mg protein

#### 2.9.5 Estimation of reduced glutathione (GSH) level

The method of Beutler et al. (1963), was followed in estimating the level of reduced glutathione (GSH). This method is based upon the development of a relatively stable yellow coloured product when 5,5^′^–dithiobis-2-nitrobenzoic acid (DTNB; Ellman’s reagent) is added to sulfhydryl compounds of which glutathione comprises the bulk in tissues. The resulting chromophoric product possesses maximum absorbance at 412 nm. About 80 µL of sample was added to 80 µL of precipitating solution, which was vortexed and centrifuged at 4,000 rpm for 5 min. Thereafter, 50 µL of the supernatant was added to 150 µL of Ellman’s reagent. The absorbance of the reaction mixture was read at 412 nm against a reagent blank using a plate reader. Serial dilutions of the GSH stock solution were prepared. The absorbance of the yellow colour formed upon the addition of Ellman’s reagent was read within 30 min at 412 nm against a blank of 1.5 mL of Ellman’s reagent and 0.5 mL phosphate buffer. A plot of absorbance against concentration of reduced GSH was then plotted. The GSH level was determined from the plot of absorbance against GSH.

#### 2.9.6 Determination of glutathione peroxidase activity

Glutathione peroxidase (GPX) activity was measured according to the procedure of Lawrence et al. (1974), with some modifications. Glutathione peroxidase is allowed to conjugate hydrogen peroxide to glutathione for a fixed period, after which the reaction is quenched. The remaining glutathione is reacted with Ellman’s reagent, and the GSH consumed is then used to measure enzyme activity. About 50 µL of phosphate buffer in a test tube, 10 µL of NaN_3_, 20 µL of GSH, 10 µL of H_2_O_2_, and 50 µL of the sample were added (added last). The reaction mixture was incubated for 3 min at 37 °C, after which 50 µL of TCA was added, and the final mixture was centrifuged at 3,000 rpm for 5 min. To 50 µL of the supernatants, 100 µL of K_2_HPO_4_ and 50 µL of DTNB were added, and the absorbance read against a reagent blank of 50 µL distilled water, 100 µL of K_2_HPO_4_ and 50 µL of DTNB at 412 nm in a microlitre plate using a plate reader.

$$\text{GSH consumed}=\text{initial GSH amount }\left(129.39\;µg\right)\;–\text{ GSH remaining }\left(µg/\text{mL}\times 4\text{ mL}\right)$$

GPX activity=GSH consumed/mg protein

=µg GSH/mg protein

#### 2.9.7 Determination of total thiol levels

Total Thiol (TSH) levels was measured according to the procedure of Owumi et al. (2020), Ellman (1959). This assay is based on the reaction of thiol groups with 5,5′-dithiobis (2-nitrobenzoic acid) (DTNB), commonly known as Ellman’s reagent, which produced a yellow-coloured compound, 2-nitro-5-thiobenzoate (TNB). The intensity of the yellow colour, measured at 412 nm using a spectrophotometer, which is directly proportional to the concentration of sulfhydryl groups in the sample. Ellman’s reagent was prepared in 0.1 M phosphate buffer (pH 8.0) at a standard concentration of 4 mg/mL. The reaction was initiated by mixing 1 mL of the tissue sample with 2 mL of phosphate buffer and 0.1 mL of Ellman’s reagent, followed by incubation at room temperature for 10–15 min. The absorbance of the reaction mixture was then recorded at 412 nm, with a blank containing only the buffer and DTNB to correct for background absorbance. The concentration of sulfhydryl groups was calculated using the molar extinction coefficient of TNB (13,600 M^−1^ cm^−1^) or by constructing a standard curve with known thiol compounds such as glutathione or cysteine.

#### 2.9.8 Oxidative stress indices and inflammatory biomarkers assay

Oxidative stress levels in the breast homogenates was determined using hydrogen peroxide (H_2_O_2_) and lipid peroxidation assays. Lipid peroxidation was determined by measuring the formation of thiobarbituric acid reactive substances (TBARS) present in the test sample according to the method of Varshney and Kale (1990), Adedara et al. (2017), Owumi et al. (2020). Hydrogen peroxide level was measured using the method described by Adedara et al. (2017). Inflammatory biomarkers were determined using the Myeloperoxidase Activity (MPO) and nitric oxide levels (NO). Myeloperoxidase (MPO) activity, an indicator of polymorphonuclear leukocyte accumulation, was determined by the modification of the method described by Trush et al. (1994). The level of NO was determined by the method of Green et al. (1982).

##### 2.9.8.1 Assessment of malondialdehyde concentration

Malondialdehyde was determined by measuring the formation of thiobarbituric acid reactive substances (TBARS) present in the test sample according to the method of Varshney and Kale, (1990), Adedara et al. (2017). An aliquot of 40 µL of the breast homogenate was mixed with 160 µL of Tris-KCl buffer, to which 50 µL of 30% TCA was added. Then 50 µL of 0.75% TBA was added and placed in a water bath for 45 min at 80 °C. This was then cooled in ice to room temperature and centrifuged at 3,000 rpm for 10 min*.* The clear supernatant was collected, and absorbance was measured against a reference blank of distilled water at 532 nm with a microplate reader. The MDA level was calculated using an extinction coefficient of 0.156 µM^−1^cm^−1^ Ádám-Vizi and Seregi, (1982).

$$\text{Malondialdehyde }\left(\text{nmole MDA}/\text{mg protein}\right)=\frac{\text{Absorbance}\times \text{volume}\;\text{of}\;\text{mixture}}{E532\;\text{nm}\times \text{volume}\;\text{of}\;\text{sample}\times \text{mg}\;\text{protein}/\text{mL}}$$

##### 2.9.8.2 Determination of hydrogen peroxide level

Hydrogen peroxide level was measured using the method described by Adedara et al. (2017). The hydrogen peroxide (H_2_O_2_) generated in the breast tissue of the rats was determined based on the method of Wolff (1994), centred on ferrous oxidation with xylenol orange. Each of the breast samples (50 µL) was added to a mixture containing 100 μM/L of xylenol orange, 250 μM/L of ammonium ferrous sulphate, 100 mmol/L of sorbitol, and 25 mmol/L of H_2_SO_4_ and vortexed. This was followed by incubation for 30 min at room temperature. The absorbance was then read spectrophotometrically at 560 nm and the values was expressed in nmol/mg protein (Rahman et al., 2023; Chrisnasari et al., 2024). The hydrogen peroxide level of the breast homogenate was determined from the absorbance and curve.

##### 2.9.8.3 Determination of myeloperoxidase activity

Myeloperoxidase (MPO) activity, an indicator of polymorphonuclear leukocyte accumulation, was determined by the modification of the method described by Trush et al. (1994). A portion (200 µL) of O- dianisidine mixture [containing 16.7 mg of o-dianisidine dihydrochloride (3,3- Dimethoxybenzidine, Fast Blue B, C_14_H_16_N_2_O_2_ Mol. Wt 244.3) in 100 mL of 50 mM phosphate buffer, pH of 6.0, plus 50 µL of dilute H_2_O_2_ (4 µL of 59% H_2_O_2_ diluted in 96 µL of dH_2_O)] was added to 7 µL of tissue homogenate (in triplicate). Three absorbance readings at 30 s intervals were taken at 460 nm using a spectrophotometer for 5 min. The MPO activity is in unit (U) of MPO/mg tissue, where one unit of MPO is defined as the amount needed to degrade 1 µmol of H_2_O_2_ per minute at room temperature. Considering that one unit (U) of MPO = 1 µmol of H_2_O_2_ split and that 1 µmol of H_2_O_2_ gives a change of absorbance of 1.13 × 10^−2^ nm/min, units of MPO in each sample is determined as change in absorbance, that is [ΔAbs (t_2_ – t_1_)]/Δmin × (1.13 × 10^−2^)]. About 200 µL of combined solutions (buffered O-dianisidine and H_2_O_2_) and 7 µL of sample in microplate and absorbance was measured at every 30 s interval for 4 min at 460 nm.

Calculation = ^A.

##### 2.9.8.4 Determination of nitric oxide level

The level of NO was determined by the method of Green et al. (1982). The amounts of nitrite in supernatants or in serum were measured following the Griess reaction by incubating 0.5 mL of the sample with 0.5 mL of Griess reagent [0.1% N-(1-naphthyl) ethylenediamine dihydrochloride; 1% sulphanilamide in 5% phosphoric acid] at room temperature for 20 min. The absorbance at 550 nm (OD 550) was measured spectrophotometrically. Nitrite concentration was calculated by comparison with the OD 550 of a standard solution of known sodium nitrite concentrations. Nitrite concentration was calculated by comparison with the optical density (OD) 550 of a standard solution of known sodium nitrite concentrations.

### 2.10 Hormonal assay

Estradiol, progesterone, prolactin, follicle stimulating hormone (FSH), Luteinizing hormone (LH) were assessed. Estradiol and progesterone assessment were done using the ELISA method. The ELISA kit was manufactured by Monobind Inc. 100 North Pointe Drive, Lake Forest, CA 92630 United States. Estradiol (Lot No. EIA-49K2I8) and progesterone (Lot No. EIA-48K2E8) levels were measured according to the manufacturer’s instructions as described by Saunders (1994) and Akhouri et al (2020).

#### 2.10.1 Histological examination of breast tissues

On sacrificing the experimental rats, their abdominal and thoracic regions were dissected and opened to expose and harvest the breast. A portion of the breast each sacrificed animal was excised, blotted, and perfused with 1.15% potassium chloride to remove all traces of haemoglobin that might contaminate the tissues. The samples were preserved and fixed in 10% buffered formal-saline and were then processed for paraffin sectioning. Sections of the breast tissues of about 5 μm thickness were obtained and fixed in 10% neutral buffered formalin. These tissues were processed for histopathology examination using a routine paraffin-wax embedded method. Sections of 5-μm thickness were stained with haematoxylin and eosin. All slides were coded before examination with a light microscope and photographed using a digital camera by a histopathologist who was blinded to control and treated groups (Adefisan et al., 2022).

#### 2.10.2 Immunohistochemical evaluation of PI3K/AkT/mTOR pathway expression

Immunohistochemistry was used as a semi-quantitative method to determine the expression of PI3K/Akt/mTOR pathway proteins in the breast tissues of the female Sprague Dawley rats. Immunostaining was performed to determine protein expression and localization in 5 µm thick paraffin-embedded tissue sections. Breast tissue blocks were sectioned using a microtome and mounted on gelatin-coated slides (Maity et al., 2013; Slaoui and Fiette, 2011). Standardized protocols were provided by the antibody manufacturers, and several dilutions were initially tested to determine optimal staining conditions. Variations in staining performance for all antibodies were further assessed on representative breast Tumor sections. A certified pathologist reviewed and validated all antibody optimizations prior to their implementation in routine assays. Following 12 h incubation in an oven to remove excess paraffin and enhance tissue adherence, sections were deparaffinized in two xylene changes and rehydrated through a series of graded ethanol washes (Wang Q. et al., 2024). Antigen retrieval was achieved by heating rehydrated sections in a 0.01M citrate buffer (pH 6.0) containing Triton X-100 at 80 °C for 30 min in a water bath, with evaporation minimized (Krenacs et al., 2010). After cooling to room temperature and rinsing in Phosphate Buffer Saline (PBS), endogenous peroxidase activity was blocked by a 15-min incubation in 5% hydrogen peroxide in 70% methanol at 37 °C in the dark. Sections were then blocked with 5% Bovine Serum Albumin (BSA) for 30 min at room temperature, followed by overnight incubation at 4 °C with specific primary antibodies in a humidified chamber. After washing in tris-buffered saline, sections were incubated for 1 hour at room temperature with horseradish peroxidase-conjugated goat anti-rabbit polyclonal secondary antibodies (Elabsciences). 3,3′-Diaminobenzidine (DAB) staining was used for detection, with hematoxylin counterstaining (Table 2). Finally, slides were cover-slipped with Diphenyl Xylene (DPX) and allowed to air-dry (Maity et al., 2013). The slides were examined under a light microscope (Nikon Diaphot, United States) and photographed using a digital camera coupled to it (Canon D50, United States).

**TABLE 2:** Details of antibodies used in immunohistochemistry procedures.

S/N	Antibiodies	Abbreviations	Dilution factor	Catalogue number
A	Genes for sustaining proliferative signaling	​		
1	Phosphoinositide 3-kinase	PI3K	1: 50–1:200	E-AB-91487
2	Protein kinase B	AKT	1:100–1:300	E-AB-30467
3	Mammalian target of rapamycin	mTOR	1:50–1:200	E-AB-15789
4	Rat sarcoma	Ras	1:100–1:300	E-AB-32152
5	Mitogen-activated protein kinase	MAPK/p38	1:100–1:300	E-AB-21027
6	Mouse double minute 2	MDM2	1:100–1:300	E-AB-31995
7	Phosphoinositide-dependent kinase 1	PDK1	1:100–1:300	E-AB-32535
8	FOrkhead boX O	FOXO	1:300–1:1,000	E-AB-70144
9	Glycogen synthase kinase 3 beta	GSK3β	1:100–1:300	E-AB-31629
10	Phosphatase and TENsin homolog deleted on chromosome 10	PTEN	1:20–1:100	E-AB-19312
11	Estrogen receptor	ER	1:76	E-AB-15624
12	Progesterone receptor	PR	1:100–1:300	E-AB-22105
13	Epidermal growth factor receptor	EGFR	1:60	E-AB-53244
​	Peroxidase HRP-conjugated	HRP-DAB	1:400–4,000	E-AB-1003
A	Genes for sustaining proliferative signaling	​	​	​
1	Phosphoinositide 3-kinase	PI3K	1: 50–1:200	E-AB-91487
2	Protein kinase B	AKT	1:100–1:300	E-AB-30467
3	Mammalian target of rapamycin	mTOR	1:50–1:200	E-AB-15789
4	Rat sarcoma	Ras	1:100–1:300	E-AB-32152
5	Mitogen-activated protein kinase	MAPK/p38	1:100–1:300	E-AB-21027
6	Mouse double minute 2	MDM2	1:100–1:300	E-AB-31995
7	Phosphoinositide-dependent kinase 1	PDK1	1:100–1:300	E-AB-32535
8	Telomerase reverse transcriptase	TERT	1:50–1:200	E-AB-12901
9	Estrogen receptor	ER	1:76	E-AB-15624
10	Progesterone receptor	PR	1:100–1:300	E-AB-22105
11	Epidermal growth factor receptor	EGFR	1:60	E-AB-53244
B	Genes for cell cycle arrest	​		
12	Retinoblastoma	Rb	1:100–1:300	E-AB-14899
13	Early region 2 binding factor	E2F	1:50–1:100	E-AB-40147
14	FOrkhead boX O	FOXO	1:300–1:1,000	E-AB-70144
15	Cyclin-dependent kinase inhibitor 1B	p27	1:50–1:200	E-AB-10569
16	Glycogen synthase kinase 3 beta	GSK3β	1:100–1:300	E-AB-31629
C	Genes for regulating genomic instability/DNA damage response	​		
17	Phosphatase and TENsin homolog deleted on chromosome 10	PTEN	1:20–1:100	E-AB-19312
18	BReast CAncer type 1 susceptibility protein	BRCA1	1:100–1:200	E-AB-40282
19	BReast CAncer type 2 susceptibility protein	BRCA2	1:100–1:200	E-AB-40288
20	Tumor related protein 53	p53	1:100–1:300	E-AB-32469
D	Genes for regulating tumor promoting inflammation/avoiding immune response	​		
21	Nuclear factor kappa B	NF-kB	1:50–1:200	E-AB-60843
22	GATA binding protein 3	GATA3	1:100–1:300	E-AB-19493
E	Genes for regulating apoptosis	​		
23	BCL2-associated X protein	BAX	1:100–200	E-AB-22128
24	BCL2-associated agonist of cell death	BAD	1:50–1:200	E-AB-13813
25	B-cell lymphoma 2 protein	BCl-2	1:50–1:100	E-AB-60012
26	Cytochrome C	Cyt-C	1:50–1:200	D-AB-10419L
27	Caspase 3	CAS-3	1:50–1:200	E-AB-13815
28	Caspase 9	CAS-9	1:100–1:300	E-AB-30760
29	Caspase 8	CAS-8	1:30–1:150	E-AB-19664
​	Peroxidase HRP-conjugated	HRP-DAB	1:400–4,000	E-AB-1003

#### 2.10.3 Statistical analysis of results

Data from the *in-vivo* toxicity and mammary tumorigenesis study were expressed as mean ± standard deviation (SD). Quantitative profiling of PI3K/Akt/mTOR pathway protein expression was performed using ImageJ (NIH) for densitometric quantification. Statistical evaluations were conducted using GraphPad Prism 8.0. Significant differences across the eight experimental groups were determined using one-way analysis of variance (ANOVA). In order to mitigate the risk of Type I errors associated with multiple pairwise comparisons, Tukey’s Honest Significant Difference (HSD) *post hoc* test was applied for inter-group comparisons. For all analyses, a *p*-value of less than 0.05 was considered statistically significant (*n* = 8 for protein expression and physiological parameters).

## 3 Results

### 3.1 Component analysis of *Pleurotus ostreatus* ethanolic extract

#### 3.1.1 Gas chromatography-mass spectroscopy (GC-MS) profile of *PoEE*

**FIGURE 2:**
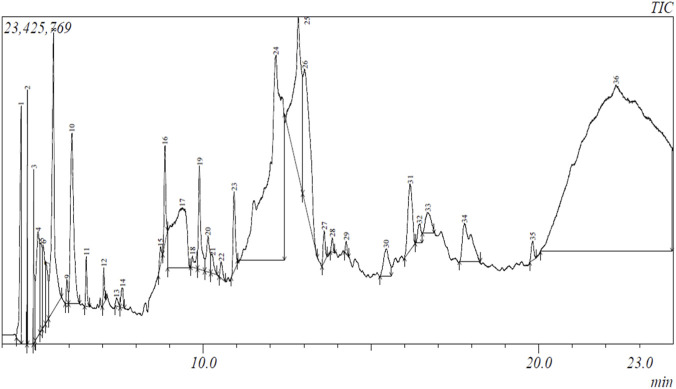
Gas chromatogram of *Pleurotus ostreatus* ethanolic extract.

The percentage yield of *PoEE* was 5.15% (w/w). The component analysis of *PoEE* revealed the presence of thirty-six (36) bioactive metabolites belonging to various metabolite classification (Figure 2; Table 3). Majority of the bioactive metabolites in the ethanol extracts were alcohols and fatty acids.

**TABLE 3:** Gas chromatography-mass spectroscopy (GC-MS) identified metabolites in *Pleurotus ostreatus* ethanolic extract.

Peak	Metabolite	Retention time (min)	Area (%)	Formula	Molecular weight
1.	Ethanol	4.568	1.20	C_2_H_6_O	46
2.	Ethanol	4.759	0.27	C_2_H_6_O	46
3.	2-Formylhistamine	4.950	0.48	C_6_H_9_N_3_O	139
4.	sec-Butylamine	5.081	1.52	C_4_H_11_N	598
5.	Acetone	5.170	0.74	C_3_H_6_O	58
6.	1-Propanol, 2-methyl	5.235	0.81	C_4_H_10_O	74
7.	Methylamine	5.335	0.60	C_3_H_9_N	59
8.	Acetic acid	5.537	3.39	C_2_H_4_O_2_	60
9.	2-Propanone	5.939	0.16	C_3_H_6_O_2_	74
10.	1-Butanol	6.083	2.41	C_5_H_12_O	88
11.	Propylene glycol	6.516	0.28	C_3_H_8_O_2_	76
12.	2,3-Butanediol	7.035	0.19	C_4_H_10_O_2_	90
13.	Pyrazine, methyl-	7.410	0.09	C_5_H_6_N_2_	94
14.	Butanoic acid	7.593	0.17	C_5_H_10_O_2_	102
15.	Pyrazine, 2,5-dimethyl	8.725	0.15	C_6_H_8_N_2_	108
16.	Butyrolactone 2(3H)-Furanone	8.858	0.68	C_4_H_6_O_2_	86
17.	L-lactic acid	9.369	3.72	C_3_H_6_O_3_	90
18.	Hexanoic acid, capronoic acid	9.680	0.20	C_6_H_12_O_2_	116
19.	2(5H)-furanone, 3-methyl	9.888	1.03	C_5_H_6_O_2_	98
20.	2H-pyran-2,6(3H)-dione	10.140	0.51	C_5_H_4_O_3_	112
21.	Pyrazine, trimethyl	10.280	0.22	C_7_H_10_N_2_	122
22.	3-Methyl-3-oxetanemethanol	10.535	0.16	C_5_H_10_O_2_	102
23.	2(3H)-furanone, dihydro-3-hydroxy-4,4-dimethyl-	10.921	0.73	C_6_H_10_O_3_	130
24.	2-Pyrrolidinone	12.163	13.33	C_4_H_7_NO	85
25.	4H-pyran-4-one	12.830	4.64	C_6_H_8_O_4_	144
26.	1-Butoxy-2-propanol acetate	13.025	4.00	C_9_H_18_O_3_	174
27.	2,4-Dimethyl-1,5-diazabicyclo [3.1.0]hexane (trans)	13.603	0.26	C_6_H_12_N_2_	112
28.	o-Tolylamino-acetic acid (4-nitro-benzylidene)-hydrazide	13.844	0.12	C_16_H_16_N_4_O_3_	312
29.	Isosorbide D-Glucitol	14.264	0.09	C_6_H_10_O_4_	146
30.	Niacin	15.445	0.64	C_6_H_5_NO_2_	123
31.	Heptane, 2,3-epoxy-	16.165	1.24	C_7_H_14_O	114
32.	2-Undecanone, 6,10-dimethyl	16.440	0.28	C_13_H_26_O	198
33.	Methoxyacetic acid, 2-tridecyl ester	16.690	0.50	C_16_H_32_O_3_	272
34.	Niacinamide	17.780	1.38	C_6_H_6_N_2_O	122
35.	Fumaric acid, ethyl 2-methylallyl ester	19.814	0.20	C_10_H_14_O_4_	198
36.	D-glucitol, 1,4-anhydro-	22.289	53.58	C_6_H_12_O_5_	164

#### 3.1.2 High performance liquid chromatography (HPLC) profile of *Pleurotus ostreatus* extracts

The HPLC profile of *P. ostreatus* ethanolic extracts revealed the presence of flavonoids and phenols and saponins (Table 4). The extracts possessed a wider array of flavonoid metabolites, followed by phenolic metabolites.

**TABLE 4:** HPLC Quantification of the flavonoids and phenolic content in *Pleurotus ostreatus* ethanolic extracts.

S/N	Metabolites (mg/kg)	*PoEE*
A	Flavonoids	​
1.	2.5-Dihydroxybenzoic acid	0.0107
2.	Caffeic acid	0.0329
3.	Gallic acid	0.0066
4.	Rutin hydrate	0.0308
5.	O-coumaric acid	0.0231
6.	Benzoic acid	76.1210
7.	Chlorogenic acid	23.6395
8.	Luteiolin	0.0594
9.	Unidentified	0.0291
10.	Unidentified	0.0280
B	Phenols	​
1.	Syringic acid	12.0117
2.	Vanillic acid	69.9143
3.	Ellagic acid	18.0513

### 3.2 *In-vivo* evaluation of the anticancer properties of *Pleurotus ostreatus* using female Sprague Dawley rats

The anti-cancer properties of *P. ostreatus* in breast cancer was evaluated using the effect on body weights (BW), tumor burden, antioxidant capacity, oxidative stress, inflammatory parameters, hormonal parameters, histological evaluation and expression of PI3K/Akt/mTOR pathway proteins.

#### 3.2.1 Effects of *Pleurotus ostreatus* ethanolic extract (*PoEE*) on body weight and tumor burden of DMBA-NMU induced female SD rats

The effects of *PoEE* on body weights of DMBA-NMU induced Female SD rats was evaluated as shown in Table 5. The data show significant differences in body weight (BW) changes and tumour mortality (M%) across treatment groups in a breast cancer model. Group A (olive oil control) had the highest weight gain (WG: 148.75 g, 200.34%), serving as a baseline (RWG = 1.00) with no mortality. In contrast, Group B (DMBA-NMU only, the carcinogen-treated group) showed reduced weight gain (104.71 g, 140.18%) and a 25% mortality rate, indicating tumour-induced health decline. Groups treated with *PoEE* (C, E, G) or vincristine (D, F) exhibited improved outcomes. Notably, Group C (*PoEE* -only) had the highest WG% (248.58%), suggesting no toxicity, while Group D (vincristine-only) showed moderate growth (143.07%) but no mortality. Combining *PoEE* with DMBA-NMU (Groups E, G) mitigated carcinogen effects, with WG% (∼142–191%) and lower mortality (12.5%) than Group B. Strikingly, Group H (DMBA-NMU + *PoEE* + vincristine) had the second-highest WG% (226.75%) but unexpectedly high mortality (37.5%), possibly due to drug interactions or toxicity at high doses.

**TABLE 5:** Effects of *PoEE* on the body weights of DMBA-NMU induced breast cancer in female Sprague Dawley Rats.

Abbreviations	Groups	Initial BW	Final BW	WG (g)	WG (%)	RWG	M (%)
A	Olive oil only	74.25 ± 21.48	223.00 ± 45.99	148.75	200.34	1.00	0.00
B	DMBA-NMU only	83.14 ± 17.19	187.86 ± 12.62	104.71	140.18	0.70	25.00*
C	*PoEE* only	62.00 ± 17.82	200.33 ± 7.87	138.33	248.58	0.93	0.00
D	Vincristine only	82.40 ± 7.00	198.60 ± 10.58	116.20	143.07	0.78	0.00
E	DMBA-NMU + *PoEE* (post)	84.80 ± 5.04	205.60 ± 27.00	120.80	142.44	0.81	12.50
F	DMBA-NMU + vincristine	75.50 ± 12.12	203.17 ± 18.76	127.25	170.39	0.86	12.50
G	DMBA-NMU + *PoEE* (pre)	69.40 ± 9.07	199.40 ± 10.61	130.00	191.57	0.87	12.50
H	DMBA-NMU + *PoEE* + vincristine	66.83 ± 16.18	208.83 ± 27.76	140.40	226.75	0.94	37.50*

Values are expressed as mean ± standard deviation (SD).

^a^ *p* < 0.05 when compared to the control.

^b^ *p* < 0.05 when compared to DMBA-NMU, only treated group.

^c^ *p* < 0.05, comparison between the co-treated groups, indicates the more potent treatment combination.

Abbreviations: W, Weight; %RW, % Relative Weight, WG, Weight gain; RWG, relative weight gain; M, mortality; Vin, Vincristine; Ext, *Pleurotus ostreatus* ethanolic extract (*PoEE*); DMBA- NMU, 7,12-dimethylbenz(α)anthracene (DMBA) and N-methyl Urea (NMU); Pre, Pre-administration of *PoEE*, before DMBA-NMU, induction; Post, Post administration of *PoEE*, after DMBA-NMU, induction.

**FIGURE 3:**
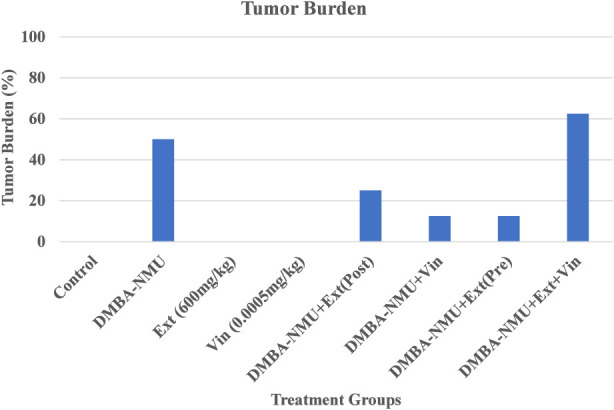
The effects of PoEE on the tumor burden in breast tissue of DMBA-NMU treated female Sprague Dawley rats. Abbreviations: Vin, Vincristine; Ext, *Pleurotus ostreatus* ethanolic extract (*PoEE*); DMBA- NMU, 7,12-dimethylbenz(α)anthracene (DMBA) and N-methyl Urea (NMU); Pre, Pre-administration of *PoEE* before DMBA-NMU induction; Post, Post administration of *PoEE* after DMBA-NMU induction.

DMBA-NMU administration successfully induced mammary tumorigenesis with a 50% incidence (4 tumors) in the disease control group, whereas the *PoEE* (600 mg/kg) and Vincristine (Vin) negative controls remained tumor-free (*p* < 0.05) (Figure 3). *PoEE* exhibited a significant timing-dependent chemopreventive effect, with prophylactic (Pre-treatment) and vincristine administration demonstrating the highest efficacy by reducing tumor incidence to 12.5% (1 tumor, *p* < 0.01). Therapeutic (Post-treatment) *PoEE* yielded a 25% incidence (2 tumors, *p* < 0.05), highlighting comparable efficacy in mitigating tumor burden. However, the combination of all three agents (DMBA-NMU + Ext + Vin) unexpectedly increased tumor incidence to 62.5% (5 tumors), suggesting complex metabolic interactions requiring further investigation.

#### 3.2.2 Effects of *Pleurotus ostreatus* ethanolic extract (*PoEE*) on antioxidant parameters in DMBA-NMU induced female Sprague Dawley rats

**FIGURE 4:**
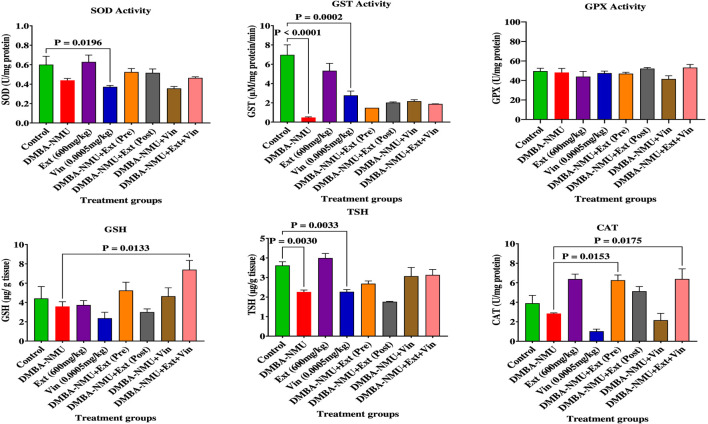
Effects of *PoEE* on antioxidant parameters in breast tissue homogenates of DMBA-NMU treated female Sprague Dawley rats Values are expressed as mean ± standard deviation (SD). Values are expressed as mean ± standard deviation (SD). *p* < 0.0001; *p* < 0.0001, *p* < 0.001, ^*^ *p* < 0.05, ^ns^ *p* > 0.05, comparison between control, DMBA-NMU and treatment groups. Abbreviations: Vin, Vincristine; Ext, *Pleurotus ostreatus* ethanolic extract (*PoEE*); DMBA, NMU - 7,12-dimethylbenz(α)anthracene (DMBA) and N-methyl Urea (NMU); Pre, Pre-administration of *PoEE* before DMBA-NMU induction; Post, Post administration of *PoEE* after DMBA-NMU induction; SOD, Superoxide dismutase; CAT, Catalase; GST, Glutathione-S-Transferase; GPX, Glutathione Peroxidase; TSH, Total Thiol; and GSH, Reduced Glutathione.

The effects of *P*. *ostreatus* ethanolic extract (*PoEE*) on antioxidant defence parameters in breast tissues were assessed by measuring enzymatic (Superoxide dismutase [SOD], Catalase [CAT], Glutathione peroxidase [GPx], Glutathione -S- Transferase [GST]) and non-enzymatic (total thiol [TSH], reduced glutathione [GSH]) activities (Figure 4). In control animals, baseline SOD activity was 0.60 U/mg protein, which decreased by 26.7% following DMBA-NMU administration (0.44 U/mg), confirming the induction of oxidative stress. *PoEE* alone enhanced SOD activity by 5% (0.63 U/mg), whereas vincristine suppressed it by 38.3% (0.37 U/mg). Preventive and therapeutic *PoEE* administration in DMBA-NMU-exposed animals preserved intermediate SOD activity (0.52 U/mg), while the combination with vincristine showed partial recovery (0.46 U/mg).

Catalase activity was more significantly modulated. Relative to controls (3.90 U/mg), DMBA-NMU reduced CAT activity by 27.2% (2.84 U/mg), and vincristine caused a more significant 73.6% suppression (1.03 U/mg). *PoEE* alone induced CAT by 63.6% (6.38 U/mg), while both preventive and therapeutic *PoEE* administration restored CAT to near-control or elevated levels (6.26 and 5.13 U/mg). Combination therapy fully restored CAT activity (6.38 U/mg), indicating strong preservation of hydrogen peroxide detoxification capacity.

Glutathione-linked enzymes displayed divergent patterns. GST activity was severely suppressed by DMBA-NMU (−93.3%; 6.97 to 0.47 U/mg). *PoEE* alone maintained 76% of control activity (5.31 U/mg), whereas combination therapies showed limited restoration (1.48–2.17 U/mg). GPx activity remained largely stable across groups (41.54–53.22 U/mg), suggesting relative resistance to oxidative perturbation.

Non-enzymatic antioxidant markers reflected treatment-specific shifts. Total thiol content was broadly reduced, with the largest decrease (−55.1%) in the post-treatment *PoEE* group. By contrast, GSH levels were significantly increased following preventive PoEE administration (+18.8% vs. control) and in combination groups (+67.8%), indicating enhanced glutathione reserve capacity. This effect was most pronounced when *PoEE* was combined with vincristine, possibly reflecting adaptive responses to dual oxidative stress.

#### 3.2.3 Effects of *PoEE* on oxidative stress indices and inflammatory biomarkers of DMBA-NMU induced BC in female SD rats

**FIGURE 5:**
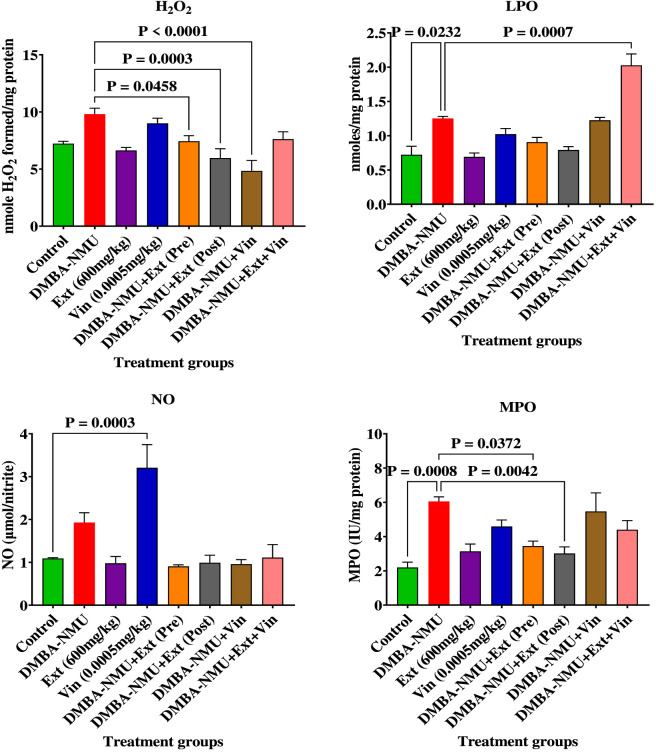
Effects of *PoEE* on oxidative stress and inflammatory biomarkers in breast tissues of DMBA-NMU treated female Sprague Dawley rats. Values are expressed as mean ± standard deviation (SD). *p* < 0.0001; *p* < 0.0001, *p* < 0.001, ^*^ *p* < 0.05, ^ns^p > 0.05, comparison between control, DMBA-NMU and treatment groups. Abbreviations: Vin, Vincristine; Ext, *P*. *ostreatus* ethanolic extract (*PoEE*); DMBA- NMU, 7,12-dimethylbenz(α)anthracene (DMBA) and N-methyl Urea (NMU); Pre, Pre-administration of *PoEE* before DMBA-NMU induction; Post, Post administration of *PoEE* after DMBA-NMU induction.

The protective effect of *PoEE* on the oxidative stress indices and inflammatory biomarkers of DMBA-NMU treated rats was assessed by evaluating the nitric oxide (NO) levels, myeloperoxidase (MPO) enzyme activity, hydrogen peroxide (H_2_O_2_) and lipid peroxidation (LPO) levels in the breast tissues as shown in Figure 5.

The effect of *PoEE* on hydrogen peroxide (H_2_O_2_) levels in DMBA-NMU-induced breast cancer in female rats was evaluated (Figure 5). No significant differences in H_2_O_2_ levels were observed among the control groups (*p* > 0.05). However, DMBA-NMU and vincristine treatments elevated H_2_O_2_ levels by 35.68% and 24.50%, respectively, compared to the olive oil control. Administration of *PoEE* prior to and after DMBA-NMU exposure significantly reduced H_2_O_2_ levels by 24.24% and 39.27%, respectively, indicating an ameliorative antioxidant effect. A more significant reduction of 50.72% (*p* < 0.05) was observed in the group co-treated with vincristine and the extract. Additionally, the combined administration of vincristine and *PoEE* to DMBA-NMU-exposed rats led to a 22.36% reduction in H_2_O_2_ levels, supporting the *PoEE*’s potential in mitigating oxidative stress.

Similarly, lipid peroxidation (LPO) levels (Figure 5) were significantly elevated following DMBA-NMU administration, with a 73.20% increase observed compared to the control group (*p* < 0.05). Vincristine treatment alone also increased LPO levels by 41.61%, while treatment with PoEE alone slightly decreased LPO levels by 4.28%. Pre- and post-treatment with the extract in DMBA-NMU-induced rats reduced LPO levels by 27.61% and 36.76%, respectively. A modest 2.08% reduction was observed in the vincristine + DMBA-NMU treatment group. However, the most substantial reduction was seen in the combined *PoEE* and vincristine treatment group, which showed a significant 62.00% decrease in LPO levels compared to the DMBA-NMU-only group (*p* < 0.05). These results further support the lipid peroxidation-inhibiting and cytoprotective effects of *PoEE*.

The effects of *PoEE* on nitric oxide (NO) levels in DMBA-NMU-induced breast cancer in female rats (Figure 5) revealed a significant elevation in NO following DMBA-NMU administration, with a 76.62% increase compared to the control group (*p* < 0.05). Vincristine alone further exacerbated this effect, causing a 193.93% increase in NO levels. However, treatment with *PoEE* alone reduced NO levels by 10.34% compared to control. Pre- and post-treatment with *PoEE* in DMBA-NMU-treated rats effectively lowered NO levels by 53.01% (*p* < 0.05) and 48.61%, respectively. Similarly, the vincristine + DMBA-NMU group showed a 50.26% reduction (*p* < 0.05), while the combined *PoEE* and vincristine treatment led to a 42.36% decrease relative to the DMBA-NMU-only group, suggesting significant anti-inflammatory potential of *PoEE*.

Myeloperoxidase (MPO) activity was also significantly elevated by 1.75 fold in the DMBA-NMU group and by 1.09 fold in the vincristine-only group compared to the control (*p* < 0.05). Pre- and post-treatment with *PoEE* markedly reduced MPO activity by 43.00% and 50.20%, respectively (*p* < 0.05) (Figure 2B). A modest reduction of 9.68% was observed in the vincristine + DMBA-NMU group, while the combination of extract and vincristine in DMBA-NMU-exposed rats resulted in a 27.30% decrease in MPO activity compared to DMBA-NMU treatment alone. These findings further support *PoEE*‘s modulatory role in oxidative and inflammatory responses in breast cancer.

#### 3.2.4 Effects of *PoEE* on hormonal parameters in the serum of DMBA-NMU induced BC in female SD rats

**FIGURE 6:**
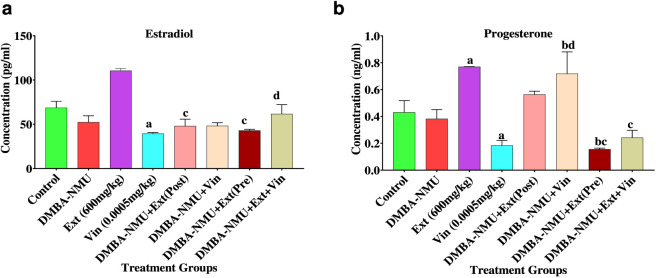
Effects of *PoEE* on estradiol **(a)** and progesterone **(b)** levels in the serum of DMBA-NMU treated female Sprague Dawley rats. Values are expressed as mean ± standard deviation (SD). ^a^ *p* < 0.05 when compared to the control; ^b^ *p* < 0.05 when compared to DMBA and NMU treated group, ^c^ *p* < 0.05, comparison to the extract treated group, and ^d^ *p* < 0.05, comparison to the vincristine treated group. Abbreviations: Vin, Vincristine; Ext, *P. ostreatus* ethanolic extract (*PoEE*); DMBA- NMU, 7,12-dimethylbenz(α)anthracene (DMBA) and N-methyl Urea (NMU); Pre, Pre-administration of *PoEE* before DMBA-NMU induction; Post, Post administration of *PoEE* after DMBA-NMU induction.

The modulatory effects of *P. ostreatus* ethanolic extract (*PoEE*) on serum reproductive hormones in DMBA-NMU–induced breast cancer rats were evaluated by measuring estradiol and progesterone (Figure 6). Administration of DMBA-NMU significantly reduced estradiol concentrations by 23.96% relative to control (52.25 ± 12.71 ng/mL), while vincristine produced a greater decline of 42.60% (39.44 ± 2.11 ng/mL). However, *PoEE* administration significantly elevated estradiol levels by 60.91% compared to control (110.56 ± 4.09 ng/mL), indicating potent estrogenic restorative effects. Co-administration of *PoEE* with vincristine partially reversed DMBA-NMU-induced suppression, raising estradiol by 18.15% relative to DMBA-NMU alone (61.73 ± 14.63 ng/mL).

Progesterone followed a similar trend. DMBA-NMU reduced serum progesterone levels by 11.27% compared to control (0.382 ± 0.098 ng/mL), while vincristine induced a higher decline of 57.26% (0.184 ± 0.065 ng/mL). *PoEE* alone significantly enhanced progesterone by 78.86% (0.770 ± 0.003 ng/mL). Preventive and post-treatment administration of *PoEE* also improved progesterone levels (0.564 ± 0.034 and 0.718 ± 0.230 ng/mL, respectively), though combined treatment with vincristine was antagonistic, lowering progesterone to 0.242 ± 0.094 ng/mL compared with extract-only treatment.

### 3.3 Effects of *Pleurotus ostreatus* extracts on the cyto-architecture of the breast

**FIGURE 7:**
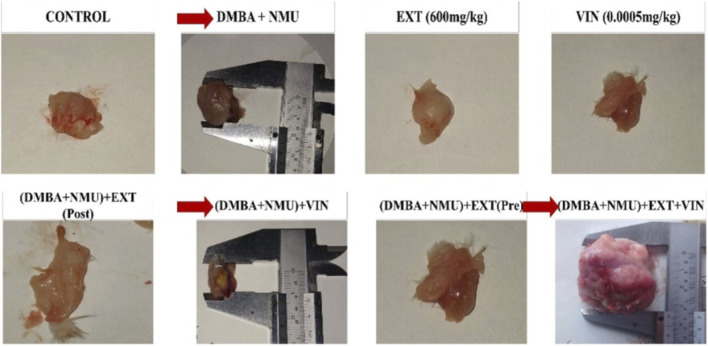
Gross examination of the breast tissues in control and treatment group showing the effects of *Pleurotus ostreatus* on DMBA-NMU induced female Sprague Dawley rat. Red arrows show groups with tumour incidence. Abbreviations: Vin, Vincristine; Ext, *P. ostreatus* ethanolic extract (PoEE); DMBA- NMU, 7,12-dimethylbenz(α)anthracene (DMBA) and N-methyl Urea (NMU); Pre, Pre-administration of PoEE before DMBA-NMU induction; Post, Post administration of PoE*E* after DMBA-NMU induction.

**FIGURE 8:**
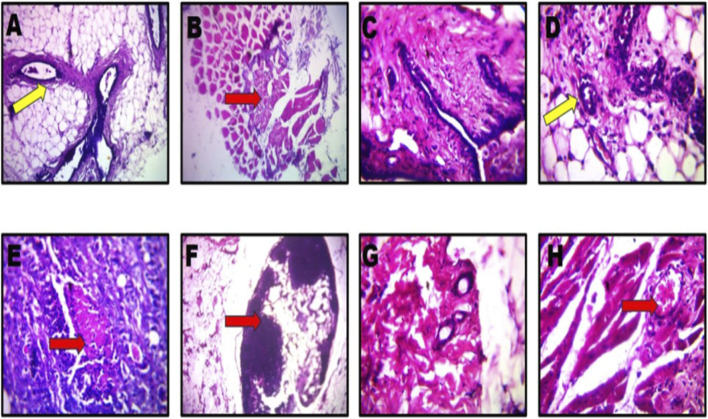
Effects of *PoEE* on the breast histology of DMBA-NMU induced breast cancer in Female Sprague Dawley rats **(A)** Olive oil only; **(B)** DMBA-NMU only; **(C)** *PoEE* only; **(D)** Vincristine only; **(E)** DMBA-NMU + *PoEE* (Post); **(F)** DMBA-NMU + Vincristine; **(G)** DMBA-NMU + *PoEE* (Pre); **(H)** DMBA-NMU + *PoEE* + Vincristine). Abbreviations: Vin, Vincristine; Ext, *P. ostreatus* ethanolic extract (PoEE); DMBA- NMU, 7,12-dimethylbenz(α)anthracene (DMBA) and N-methyl Urea (NMU); Pre, Pre-administration of *PoEE* before DMBA-NMU induction; Post, Post administration of *PoEE* after DMBA-NMU induction.

Histological analysis of mammary gland tissues revealed distinct morphological alterations across treatment groups in Figures 7, 8. The control group (olive oil only) and those treated solely with *P*. *ostreatus* extract or vincristine exhibited normal mammary architecture, including intact ducts and well-organized adipose tissue, with no signs of atypia or inflammation. In contrast, the DMBA-NMU-induced group showed hallmark features of mammary carcinoma, such as disorganized, pleomorphic epithelial clusters with hyperchromatic nuclei invading surrounding stroma. Vincristine-treated cancer rats exhibited partial tumor regression with reduced cellularity and signs of degeneration. Notably, both pre- and post-treatment with *P*. *ostreatus* extract led to evident tumor suppression, including necrosis, stromal fibrosis, and diminished tumor cell density. The most significant histological improvement was observed in the combined vincristine and extract-treated group, which demonstrated marked tumor regression, extensive fibrosis, and minimal residual neoplastic tissue, suggesting a synergistic therapeutic effect.

### 3.4 Effects of *PoEE* on PI3K/AkT/mTOR pathway protein expression in DMBA-NMU induced BC in female SD rats

Immunohistochemical analysis of mammary tissues demonstrated significant dysregulation of oncogenic and tumor suppressor proteins in PI3K/Akt/mTOR signaling (Supplementary Figures S1–S30). The proteins assessed includes: Estrogen receptor (ER), Progesterone Receptor (PR), Epidermal Growth Factor Receptor (EGFR), Phosphoinositide 3-kinase (PI3K), Protein Kinase B (AKT), Mammalian Target of Rapamycin (mTOR), Rat Sarcoma (Ras), Mitogen-Activated Protein Kinase (MAPK/p38), Mouse Double Minute 2 (MDM2), Phosphoinositide-Dependent Kinase 1 (PDK1); FOrkhead boX O (FOX O); Glycogen synthase kinase 3 beta (GSK3β); Phosphatase and TENsin homolog deleted on chromosome 10 (PTEN); Telomerase reverse transcriptase (TERT); c-Myc (cellular myelocytomatosis oncogene); Retinoblastoma protein (Rb); E2F (E2 promoter-binding factor; BRCA1 (Breast Cancer gene 1) and BRCA2 (Breast Cancer gene 2); p53 (tumor protein 53); NF-κB (nuclear factor kappa-light-chain-enhancer of activated B cells; GATA3 (GATA binding protein 3); BAX (Bcl-2-associated X protein) and BAD (Bcl-2-associated death promoter); Bcl-2 (B-cell lymphoma 2); cytochrome c (Cyt-c); caspase-3 (Cas-3); caspase-9 (Cas-9); and caspase-8 (Cas-8).

#### 3.4.1 Expression of proteins for sustaining proliferative signalling

**FIGURE 9:**
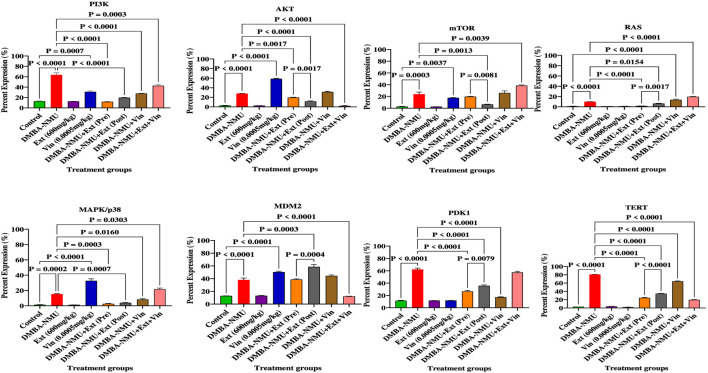
Expression of proteins for cell proliferation in breast tissues of DMBA-NMU induced breast cancer in female Sprague-Dawley Rats Values are expressed as mean ± standard deviation (SD). *p* < 0.0001; *p* < 0.0001, *p* < 0.001, ^*^ *p* < 0.05, ^ns^p > 0.05, comparison between control, DMBA-NMU and treatment groups. Abbreviations: Vin, Vincristine; Ext, *Pleurotus ostreatus* ethanolic extract (*PoEE*); DMBA- NMU, 7,12-dimethylbenz(α)anthracene (DMBA) and N-methyl Urea (NMU); Pre, Pre-administration of *PoEE* before DMBA-NMU induction; Post, Post administration of *PoEE* after DMBA-NMU induction.

The administration of DMBA-NMU triggered significant activation of the PI3K/Akt/mTOR and MAPK signaling axes (Figure 9). Compared to the control, DMBA-NMU significantly upregulated key oncogenic markers, including PI3K (63.63% ± 5.51%), Akt (27.78% ± 0.56%), mTOR (23.84% ± 5.19%), Ras (9.96% ± 0.26%), MAPK/p38 (15.31% ± 0.69%), MDM2 (38.06% ± 4.05%), PDK1 (62.23% ± 2.95%), and TERT (79.25% ± 1.15%) (*p* < 0.0001 for all). *PoEE* alone maintained a non-oncogenic profile similar to the control, vincristine (Vin) monotherapy presented a mixed response. Although Vin maintained low levels of PI3K, Ras, and PDK1, it induced significant compensatory upregulation of Akt (58.63% ± 1.56%), MAPK/p38 (32.65% ± 3.49%), and MDM2 (50.38% ± 1.68%) relative to the control (*p* < 0.0001), suggesting limited efficacy as a standalone agent. *PoEE* treatment effectively attenuated oncogenic signaling in both preventive and therapeutic models. Preventive administration (Ext + DMBA-NMU) significantly blunted protein induction, suppressing PI3K (11.65% ± 1.47%), Akt (19.72% ± 0.64%), Ras (1.06% ± 0.15%), and TERT (24.75% ± 0.45%) compared to the DMBA-NMU group (*p* < 0.05). Therapeutic post-treatment with *PoEE* similarly reduced DMBA-NMU-induced expression of PI3K (19.72% ± 0.64%), mTOR (6.54% ± 0.53%), and MAPK/p38 (3.76% ± 0.74%) (*p* < 0.05). Notably, the post-treatment regimen demonstrated superior efficacy in reducing mTOR and MAPK/p38 levels compared to the preventive model. Combination therapy (DMBA-NMU + Ext + Vin) revealed selective synergistic downregulation of specific proliferative nodes. This combination significantly enhanced the suppression of Akt (2.16% ± 1.60%) and MDM2 (13.25% ± 0.15%) compared to both DMBA-NMU and individual treatments (*p* < 0.0001), while reducing TERT expression to 19.85% ± 0.55%. However, the combination was less effective at suppressing PI3K (42.71% ± 3.45%) and mTOR (38.63% ± 1.56%) than *PoEE* monotherapy, indicating complex pharmacological interactions between the extract and the chemotherapeutic drug.

#### 3.4.2 Expression of cell cycle regulatory proteins

**FIGURE 10:**
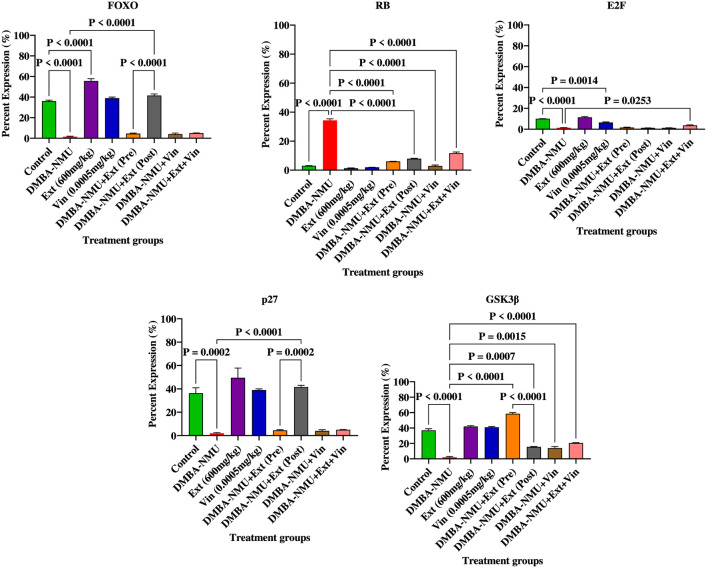
Expression of proteins for cell cycle regulation in breast tissues of DMBA-NMU induced breast cancer in female Sprague-Dawley Rats. Values are expressed as mean ± standard deviation (SD). *p* < 0.0001; *p* < 0.0001, *p* < 0.001, ^*^ *p* < 0.05, ^ns^p > 0.05, comparison between control, DMBA-NMU and treatment groups. Abbreviations: Vin, Vincristine; Ext, *P. ostreatus* ethanolic extract (*PoEE*); DMBA- NMU, 7,12-dimethylbenz(α)anthracene (DMBA) and N-methyl Urea (NMU); Pre, Pre-administration of *PoEE* before DMBA-NMU induction; Post, Post administration of *PoEE* after DMBA-NMU induction.

The administration of DMBA-NMU significantly disrupted cell cycle homeostasis, characterized by a significant suppression of tumor suppressor proteins and the induction of oncogenic regulators (Figure 10). Specifically, DMBA-NMU treatment inibited the expression of FOXO (*p* < 0.0001), p27 (P = 0.0002), and GSK3β (*p* < 0.0001) compared to the control group. This loss of inhibitory control was accompanied by a significant upregulation of RB (*p* < 0.0001) and a concomitant depletion of E2F (P = 0.0001), reflecting an aggressive proliferative state induced by the carcinogen. *PoEE* treatment exhibited a protective regulatory profile, significantly elevating FOXO and p27 levels compared to both control and DMBA-NMU groups. In contrast, vincristine (Vin) monotherapy showed limited efficacy in restoring these regulatory proteins, failing to significantly recover p27 and GSK3β levels from the levels observed in the control. Intervention with *PoEE*, particularly in the preventive (Pre) and therapeutic (Post) models, effectively reversed DMBA-NMU-induced cell cycle dysregulation. Preventive administration of *PoEE* significantly restored GSK3β expression (*p* < 0.0001), while post-treatment was markedly more effective in recovering FOXO (*p* < 0.0001) and p27 (P = 0.0002) levels. Also, *PoEE* post-treatment successfully reduced RB expression back toward control levels. Combination therapy (DMBA-NMU + *PoEE*+ Vin) demonstrated varied effects; while it significantly suppressed RB expression (*p* < 0.0001), it was less effective at restoring the key tumor suppressors FOXO, p27, and GSK3β compared to *PoEE* monotherapy.

#### 3.4.3 Expression of proteins for regulating genomic instability/DNA damage response (DDR)

**FIGURE 11:**
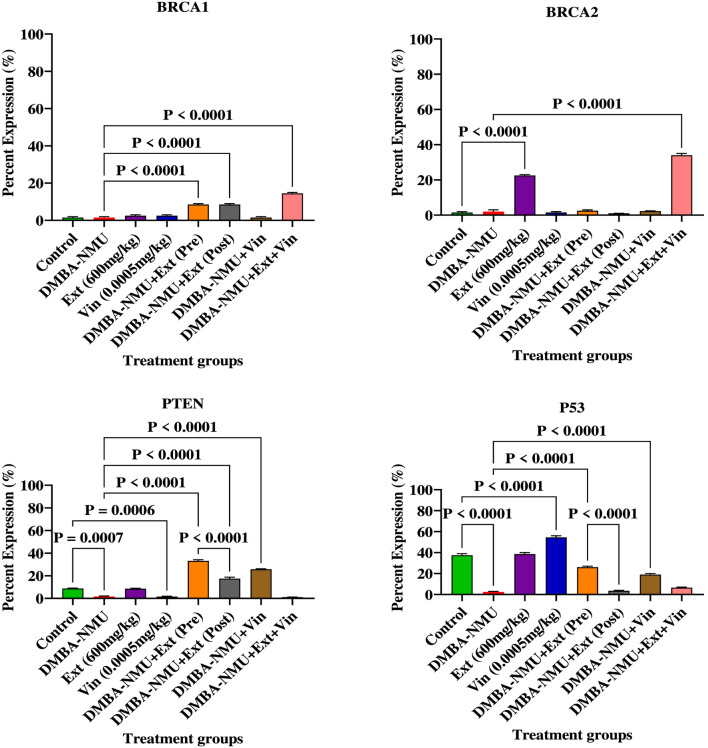
Expression of proteins for regulating genomic instability/DNA damage response (DDR) in breast tissues of DMBA-NMU induced breast cancer in female Sprague-Dawley Rats. Values are expressed as mean ± standard deviation (SD). *p* < 0.0001; *p* < 0.0001, *p* < 0.001, ^*^ *p* < 0.05, ^ns^p > 0.05, comparison between control, DMBA-NMU and treatment groups. Abbreviations: Vin, Vincristine; Ext, *P. ostreatus* ethanolic extract (*PoEE*); DMBA- NMU, 7,12-dimethylbenz(α)anthracene (DMBA) and N-methyl Urea (NMU); Pre, Pre-administration of *PoEE* before DMBA-NMU induction; Post, Post administration of *PoEE* after DMBA-NMU induction.

The administration of DMBA-NMU significantly compromised the DNA damage response (DDR) framework, evidenced by the near-complete suppression of key tumor suppressor proteins (Figure 11). Compared to the control group, DMBA-NMU treatment resulted in a drastic reduction in the expression of PTEN (P = 0.0007), p53 (*p* < 0.0001), BRCA1, and BRCA2. This depletion of genomic biomarkers indicates a state of heightened genomic instability and impaired repair capacity induced by the carcinogen. *PoEE* treatment exhibited a protective profile by significantly enhancing the baseline expression of DDR proteins. Specifically, PoEE treatment significantly elevated BRCA2 (*p* < 0.0001) and maintained high levels of p53 and PTEN relative to the DMBA-NMU group. In contrast, vincristine (Vin) monotherapy failed to adequately restore these pathways, showing negligible recovery of BRCA1 and BRCA2 levels and only a modest increase in p53 expression compared to the control. Intervention with *PoEE* in both preventive (Pre) and therapeutic (Post) models effectively reversed the DMBA-NMU-induced suppression of the DDR machinery. Preventive administration [DMBA-NMU + *PoEE* (Pre)] demonstrated the most substantial recovery, significantly upregulating PTEN (*P* < 0.0001), p53 (*P* < 0.0001), and BRCA1 (*P* < 0.0001) compared to the DMBA-NMU group. Post-treatment with PoEE also yielded significant therapeutic benefits, restoring PTEN (*P* < 0.0001) and BRCA1 (*P* < 0.0001) levels, though its effect on p53 was less pronounced than the preventive model. The combination therapy (DMBA-NMU + *PoEE* + Vin) demonstrated potent synergistic effects in enhancing specific DDR components. Most notably, the combination significantly increased BRCA1 (*P* < 0.0001) and BRCA2 (*P* < 0.0001) expression beyond the levels achieved by any individual treatment. Furthermore, it effectively restored PTEN and p53 levels compared to the DMBA-NMU group (*P* < 0.0001), suggesting that the integration of *PoEE* with vincristine may stabilize the genome and enhance DNA repair efficiency more effectively than monotherapy.

#### 3.4.4 Expression of proteins for regulating tumor-promoting inflammation/avoiding immune response

**FIGURE 12:**
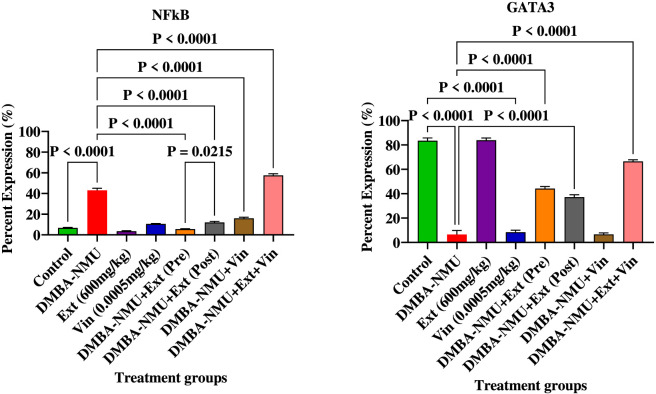
Expression of proteins for regulating tumor-promoting inflammation/avoiding immune response in breast tissues of DMBA-NMU induced breast cancer in female Sprague-Dawley Rats. Values are expressed as mean ± standard deviation (SD). *p* < 0.0001; *p* < 0.0001, *p* < 0.001, ^*^ *p* < 0.05, ^ns^p > 0.05, comparison between control, DMBA-NMU and treatment groups. Abbreviations: Vin, Vincristine; Ext, *P. ostreatus* ethanolic extract (*PoEE*); DMBA- NMU, 7,12-dimethylbenz(α)anthracene (DMBA) and N-methyl Urea (NMU); Pre, Pre-administration of *PoEE* before DMBA-NMU induction; Post, Post administration of *PoEE* after DMBA-NMU induction.

The induction of breast cancer *via* DMBA-NMU established a significant inflammatory and immunosuppressive microenvironment (Figure 12). This was primarily evidenced by a robust upregulation of the pro-inflammatory transcription factor NFkB (*P* < 0.0001) and a critical depletion of GATA3 (*P* < 0.0001), a key regulator of immune response and mammary cell differentiation. The administration of *PoEE* alone maintained baseline levels similar to the control, vincristine (Vin) monotherapy failed to reverse the inflammatory state, showing negligible impact on restoring GATA3 expression. Intervention with *PoEE*, particularly in the preventive (Pre) and therapeutic (Post) models, effectively mitigated tumor-promoting inflammation. Preventive administration of *PoEE* reduced the expression of NFkB (*P* < 0.0001) and significantly restored GATA3 expression (*P* < 0.0001) compared to the DMBA-NMU group. Therapeutic post-treatment of *PoEE* similarly reduced NFkB activation (*P* < 0.0001) and provided substantial recovery of GATA3 levels (*P* < 0.0001), suggesting a potent ability to reprogram the immune-inflammatory axis. The combination therapy (DMBA-NMU + *PoEE* + Vin) demonstrated complex interactions in immune regulation. The addition of Vin to *PoEE* resulted in the most significant restoration of GATA3 (*P* < 0.0001), it paradoxically induced a marked increase in NFkB expression (*P* < 0.0001) compared to the DMBA-NMU group. This suggests that while the combination may enhance certain aspects of immune signaling *via* GATA3, it may also trigger compensatory inflammatory responses that require careful pharmacological consideration.

#### 3.4.5 Expression of proteins for regulating apoptosis

**FIGURE 13:**
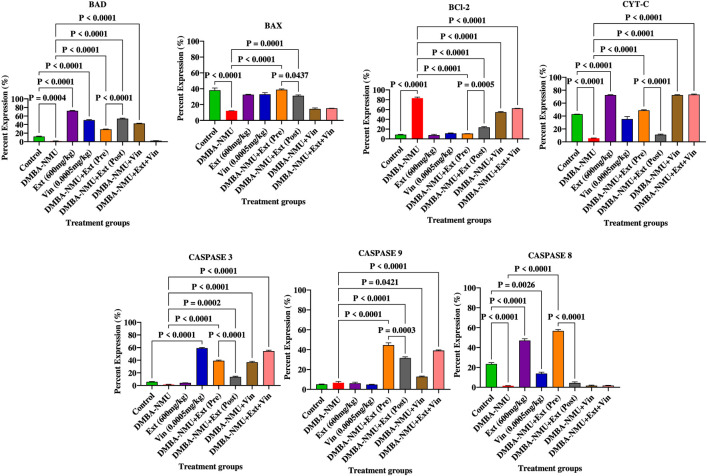
Expression of proteins for regulating apoptosis in breast tissues of DMBA-NMU induced breast cancer in female Sprague-Dawley Rats. Values are expressed as mean ± standard deviation (SD). *p* < 0.0001; *p* < 0.0001, *p* < 0.001, ^*^ *p* < 0.05, ^ns^p > 0.05, comparison between control, DMBA-NMU and treatment groups. Abbreviations: Vin, Vincristine; Ext, *P. ostreatus* ethanolic extract (*PoEE*); DMBA- NMU, 7,12-dimethylbenz(α)anthracene (DMBA) and N-methyl Urea (NMU); Pre, Pre-administration of *PoEE* before DMBA-NMU induction; Post, Post administration of *PoEE* after DMBA-NMU induction.

The administration of DMBA-NMU effectively suppressed the apoptotic machinery, facilitating tumor cell survival (Figure 13). Compared to the control group, DMBA-NMU induction resulted in the significant downregulation of pro-apoptotic markers, including BAD (P = 0.0004), BAX (*P* < 0.0001), CYT-C (*P* < 0.0001), Caspase 3 (*P* < 0.0001), and Caspase 8 (*P* < 0.0001). However, the anti-apoptotic regulator Bcl-2 was markedly upregulated (*P* < 0.0001 vs. control), establishing a high Bcl-2/Bax ratio indicative of apoptosis resistance. *PoEE* treatment exhibited a strong pro-apoptotic profile, significantly increasing the expression of BAD, CYT-C, and Caspase 8 (*P* < 0.0001) relative to the DMBA-NMU group, while maintaining Bcl-2 at baseline levels. In contrast, vincristine (Vin) monotherapy showed selective activity, significantly elevating Caspase 3 (*P* < 0.0001) and BAD levels, but it was less effective than *PoEE* in restoring CYT-C or Caspase 9 expression. Both preventive and therapeutic interventions with *PoEE* successfully re-established apoptotic signaling. Preventive administration [DMBA-NMU + *PoEE* (Pre)] significantly enhanced BAX (*P* < 0.0001), Caspase 3 (*P* < 0.0001), Caspase 9 (*P* < 0.0001), and Caspase 8 (*P* < 0.0001) expression compared to the DMBA-NMU group. Therapeutic post-treatment [DMBA-NMU + *PoEE* (Post)] was effective in restoring BAD and CyT-C (*P* < 0.0001), suggesting a significant reactivation of the intrinsic mitochondrial pathway. Both *PoEE* regimens successfully attenuated DMBA-NMU-induced Bcl-2 expression (*P* < 0.0001). Combination therapy (DMBA-NMU + *PoEE* + Vin) demonstrated a complex apoptotic profile. The combination significantly upregulated Bcl-2 (*P* < 0.0001) while simultaneously inducing high expression of Caspase 3 and Caspase 9 (*P* < 0.0001). However, the combination led to a near-total loss of BAD and Caspase 8 expression (*P* < 0.0001), indicating that while the combination triggers executioner caspases, it may bypass or inhibit certain upstream regulatory nodes.

## 4 Discussion

The PI3K/Akt/mTOR pathway, when overactive, plays a key role in breast cancer development, especially in hormone receptor-negative subtypes that do not respond to standard hormone therapies (Effiong et al., 2025a; Hassan and Aubel, 2025). This pathway controls cell growth, survival, and metabolism, and when disrupted, it leads to worse outcomes and treatment resistance (Garg et al., 2025). As a result, natural phytochemicals with anti-cancer activity are being sought out to offer effective treatment remedies with less associated toxicities, as compared to conventional anti-cancer drugs. *Pleurotus ostreatus*, a widely consumed mushroom has been reported to contain an array of bioactive metabolites with high antioxidant properties (Effiong et al., 2024a; Effiong et al., 2024b). In this study, the mechanism of action of the ethanolic extract of *P*. *ostreatus* (oyster mushroom) in preventing and treating breast cancer by targeting multiple points in this pathway, blocking cancer-promoting signals while restoring normal cellular controls was evaluated. This study evaluated the potent chemopreventive effects of *P. ostreatus* ethanolic extract (*PoEE*) against DMBA-NMU-induced breast carcinogenesis, with significant modulation of oxidative stress, inflammation, DNA repair, cell cycle regulation, cell proliferation and apoptosis pathways. The findings support the multifaceted anti-cancer potential of *PoEE* and provide mechanistic insights into its biochemical actions.

The distinct effects of *P*. *ostreatus* ethanolic extract (*PoEE*) and vincristine on body weight and tumor-related mortality in the DMBA-NMU-induced breast cancer model highlight important mechanistic differences in their modes of action. DMBA-NMU alone reduced weight gain and higher mortality in rats, indicating metabolic stress and oxidative damage, similar to reports by Adefisan-Adeoye et al. (2025). *PoEE*-only treatment promoted weight gain without mortality, which negates the findings of Ganesan and Xu (2018), demonstrating its antioxidant, anti-inflammatory, and metabolic regulatory effects. When administered alongside DMBA-NMU, *PoEE* offered partial protection, improving metabolic balance and reducing mortality through suppression of oxidative DNA damage and oncogenic signalling, similar to reports by Naguib et al. (2014), Iqbal et al. (2024). Vincristine alone reduced weight gain due to its cytotoxicity but caused no mortality, similar to reports by López-Tofiño et al. (2024), Sztukowski et al. (2024), Donovan et al. (2023), Takahashi et al. (2015). The combination of *PoEE* and vincristine increased weight gain but elevated mortality, suggesting that *PoEE* ameliorated vincristine’s systemic toxicity and supported metabolism but attenuated its anti-tumor efficacy by activating survival pathways or altering drug bioavailability. The study suggests that *PoEE* provides significant chemopreventive and protective benefits as a standalone agent, but its interaction with vincristine may be antagonistic, requiring careful optimization of combination regimens.

The selection of a 600 mg/kg daily dose for *P. ostreatus* ethanolic extract (PoEE) was strategically based on established protocols that demonstrate peak chemopreventive efficacy in rodent mammary models Krishnamoorthy and Sankaran (2016). The 600 mg/kg dose represents a robust pharmacological challenge for a chronic 25-week study, the regimen was highly tolerable, with no mortality or overt signs of clinical toxicity observed across the initiation, promotion, and progression stages. This safety profile is supported by previous acute and sub-chronic studies where *P. ostreatus* extracts showed no adverse effects at doses up to 600 mg/kg, likely due to the high biocompatibility of mushroom-derived polysaccharides and phenolic metabolites (Effiong et al., 2025b; Krishnamoorthy and Sankaran, 2016). In order to facilitate clinical translatability, the animal dosage was converted to a Human Equivalent Dose (HED) using the body surface area (BSA) normalization method, according to standard allometric scaling, for a 600 mg/kg rat dose, the HED is approximately 97.3 mg/kg. Relating to a standard 60 kg adult, this equates to a daily oral intake of approximately 5.8 g of extract (Nair and Jacob, 2016). This value lies within the feasible therapeutic range for nutraceutical intervention; as many clinical trials investigating mushroom-derived beta-glucans utilize daily doses ranging from 3 to 10 g without significant gastrointestinal or systemic toxicity (Ito et al., 2014; Venturella et al., 2021).

Free radicals are unstable molecules produced in the body, that can cause damage to cells through a process known as oxidative stress (Salemcity et al., 2020). Antioxidants are molecules that neutralize free radicals. They consist of enzymatic and non-enzymatic subtypes (Nwozo et al., 2023; Nwozol and Effiong, 2019). *Pleurotus ostreatus* have been reported to be rich in phytochemicals such as phenols and flavonoids which possess high antioxidant properties (Effiong et al., 2025a; Effiong et al., 2024a). The administration of DMBA-NMU suppressed key antioxidant enzymes (SOD, CAT, GST) and depleted non-enzymatic thiols, reducing the capacity to scavenge free radicals and resulting in oxidative stress, similar to reports by Adefisan-Adeoye et al. (2025), Adefisan et al. (2022) on the individual mechanism of action of DMBA and NMU respectively. This reflects a classical mechanism of carcinogen-driven redox stress, in which polycyclic hydrocarbons such as DMBA undergo metabolic activation, generating ROS (superoxide anions, hydrogen peroxide, hydroxyl radicals) that overwhelm endogenous antioxidant defenses, leading to DNA adduct formation and initiation of tumorigenesis (Adefisan-Adeoye et al., 2025; Ma et al., 2018). *PoEE* effectively counteracted the oxidative stress induced by DMBA-NMU through enzymatic and non-enzymatic antioxidant pathways, indicating preventive and therapeutic function by restoring superoxide and hydrogen peroxide detoxification capacity (*via* SOD and CAT), preserving phase II conjugative defense (GST), and enhancing non-enzymatic thiol buffering (GSH). These biochemical effects directly mitigate ROS-driven DNA damage and signaling perturbations, contributing to its observed chemo-preventive efficacy against DMBA–NMU–induced mammary carcinogenesis.

Oxidative stress is a well-established hallmark of carcinogenesis, as reactive oxygen species (ROS) such as hydrogen peroxide (H_2_O_2_) and lipid peroxides induce DNA damage and oncogenic mutations (Chandimali et al., 2025; Dong et al., 2025; Ma et al., 2025). The significant elevation in H_2_O_2_ and lipid peroxidation (LPO) levels following DMBA-NMU exposure is consistent with studies showing that polycyclic aromatic hydrocarbons induce ROS accumulation *via* cytochrome P450 metabolism (Fukugami et al., 2025; Ni et al., 2025; Yu et al., 2022). *PoEE*’s marked reduction of both H_2_O_2_ and LPO levels suggests potent antioxidant activity, likely mediated by bioactive phenolic metabolites and polysaccharides inherent in *P. ostreatus*. The co-treatment with vincristine and *PoEE* synergistically reduced H_2_O_2_ and LPO, highlighting the adjunctive chemoprotective role of *PoEE*. Nitric oxide (NO) and myeloperoxidase (MPO) are inflammatory mediators associated with tumor progression (Kim and Lee, 2025). Elevated NO levels can induce DNA deamination, while MPO promotes oxidative DNA lesions such as 8-oxoguanine (Zhou et al., 2025). *PoEE*’s significant suppression of NO and MPO activity suggests interference with iNOS and neutrophil infiltration, respectively. This aligns with findings by Jedinak et al. (2011), Jayasuriya et al. (2020), Choi et al. (2025), who reported that mushroom extracts exert anti-inflammatory activity *via* NFκB pathway inhibition. The vincristine–*PoEE* combination slightly diminished this effect, possibly due to conflicting redox dynamics.

### 4.1 Effects of *PoEE* on upstream proteins in the PI3K/AkT/mTOR pathway

Among these mechanisms, the PI3K/Akt/mTOR pathway is a central oncogenic signaling axis in breast cancer, driving cell survival, proliferation, and therapy resistance (Udobi et al., 2026), and its modulation by *PoEE* suggests a critical role in restraining tumor progression (Effiong et al., 2025a; Hassan and Aubel, 2025). The observed dysregulation of PI3K/Akt/mTOR signaling in DMBA-NMU–induced breast cancer reflects the central role of this pathway in hormone-sensitive tumorigenesis. The marked upregulation of ER and PR indicates that DMBA-NMU drives a luminal-like breast cancer phenotype, which is particularly dependent on estrogen-driven mitogenic signaling, similar to reports by Adefisan-Adeoye et al. (2025). This hormonal activation converges on PI3K/Akt/mTOR and Ras/MAPK cascades, amplifying proliferative and anti-apoptotic signals. Elevated PI3K and PDK1 levels likely facilitated high Akt phosphorylation, which in turn activated mTOR to promote protein synthesis, cell growth, and angiogenesis (Anifowose et al., 2023). Simultaneous overexpression of MDM2 suggests enhanced degradation of p53, thereby bypassing genomic surveillance mechanisms (Zeng et al., 2024). Suppression of PTEN, GSK3β, and FOXO further illustrates how tumor suppressor proteins were downregulated, collectively producing a permissive environment for unchecked proliferation, survival, and therapy resistance (Glaviano et al., 2023).

The administration of *P. ostreatus* ethanolic extract (*PoEE*) effectively countered these oncogenic alterations through multiple biochemical mechanisms (Effiong et al., 2025b). Preventive administration of *PoEE* showed the most significant impact, suggesting that early intervention can block the initial activation of PI3K/Akt/mTOR and Ras/MAPK signaling before tumor establishment, which corresponds with its reported *in silico* anticancer activity by Effiong et al. (2025a). The restoration of PTEN, GSK3β, and FOXO is particularly important, as PTEN directly antagonizes PI3K activity, GSK3β restricts β-catenin–mediated proliferation, and FOXO transcription factors re-establish pro-apoptotic and cell-cycle arrest programs, shifting the balance from growth promotion to tumor suppression (Glaviano et al., 2023). Therapeutic post-treatment with *PoEE* also showed significant anticancer effect, although less when compared to the preventive use, indicating that while *PoEE* can attenuate established oncogenic signaling, its maximal efficacy lies in chemoprevention.

Vincristine monotherapy only partially suppressed oncogenic drivers and upregulated Akt and MAPK, a pattern consistent with adaptive resistance mechanisms in conventional chemotherapeutic use. However, its combination with *P. ostreatus* ethanolic extract (*PoEE)* produced selective synergy, notably in suppressing ER, PR, Akt, and MDM2, suggesting that *PoEE* enhanced vincristine’s apoptotic and anti-proliferative potential. The antagonistic upregulation of mTOR and Ras in the combination group indicates compensatory activation within the PI3K/Akt/mTOR cascade, which is often linked to pathway cross-talk and negative feedback loops. This antagonism may also reflect pharmacokinetic or metabolic interactions between vincristine and mushroom-derived metabolites, such as ergothioneine, β-glucans, and triterpenoids, which can modulate cytochrome p450 activity and drug efflux transporters (Wanwimolruk and Prachayasittikul, 2014).

The present findings reinforce the notion that *PoEE* acts through dual mechanisms, direct attenuation of the PI3K/Akt/mTOR pathway and restoration of tumor suppressor networks (PTEN, FOXO, GSK3β) while concurrently normalizing oxidative and endocrine homeostasis. The *PoEE* dose used (600 mg/kg) corresponds to a human equivalent dose of approximately 97 mg/kg, suggesting potential feasibility for clinical use. *PoEE* demonstrated strong multi-pathway anticancer activity, mitigating oxidative stress, hormonal imbalance, and PI3K/Akt/mTOR-driven proliferation (Effiong et al., 2025b). Notably, the simultaneous upregulation of Ras and mTOR in the *PoEE* and vincristine combination group presents a significant mechanistic finding regarding drug-extract interactions. The elevation of these specific markers suggests the activation of compensatory signaling feedback loops. In oncology, the potent inhibition of downstream effectors can frequently relieve the negative feedback inhibition typically exerted by mTORC1 on upstream receptors and the Ras/MAPK cascade. This “rebound” activation likely represents an adaptive survival response by the mammary tumor cells to bypass the pharmacological blockade induced by the combination therapy. The rise in mTOR levels, in particular, may reflect a shift toward mTORC2-mediated survival or a stress-induced metabolic adaptation. These results underscore the plastic nature of the signaling architecture in this model and suggest that while *PoEE* enhances the efficacy of vincristine, the tumor microenvironment engages alternative nodes to evade total apoptotic collapse—a phenomenon that warrants further investigation into multi-target “bypass” inhibitors (Krishnamoorthy and Sankaran, 2016; Adefisan et al., 2022).

### 4.2 Effects of *PoEE* on downstream proteins in the PI3K/AkT/mTOR pathway

At the core of cancer cell immortality is the sustained expression of telomerase, driven by TERT and its transcriptional regulator c-Myc (Khattar and Tergaonkar, 2017; Robinson and Schiemann, 2022). TERT ensures telomere elongation, allowing cells to evade replicative senescence, while c-Myc promotes uncontrolled proliferation *via* the transcriptional activation of E2F targets and repression of cell cycle inhibitors such as p21 and p27 (Liu et al., 2025; Sias et al., 2025). *POEE* significantly downregulated both hTERT and c-Myc, suggesting inhibition of telomerase-driven replication and proliferative signalling. This suppression is consistent with observed increases in tumor suppressors FOXO and p27, both of which are negatively regulated by c-Myc and Akt. In contrast, vincristine, while effective at downregulating c-Myc, was less potent at reducing hTERT, potentially due to its microtubule-disrupting mechanism that targets mitosis rather than transcriptional or epigenetic regulation.

The *POEE*-induced downregulation of Rb, together with context-dependent modulation of E2F, may reflect dynamic cell cycle checkpoint control, where reactivation of the Rb/E2F axis underlies growth arrest or apoptotic priming (Pellarin et al., 2025; Swiss and Casaccia, 2010; Zhou et al., 2023). The combination of *POEE* and vincristine led to significant E2F upregulation, potentially due to feedback from enhanced tumor suppressor signaling or differential stabilization of E2F complexes under dual treatment pressure (Zhou et al., 2023).

The results also demonstrate that *POEE* efficiently restores the expression of the tumor suppressor PTEN, a critical antagonist of PI3K/Akt signaling. As PTEN dephosphorylates PIP3 to PIP2, thereby inhibiting Akt activation, its re-expression likely contributed to the observed reductions in downstream targets such as mTOR, TERT, and BCl-2. PTEN restoration also coincided with upregulation of FOXO, p27, and GSK3β, all targets of Akt-dependent phosphorylation and degradation, further reinforcing the suppressive effect of *POEE* on this oncogenic axis (Hassan and Aubel, 2025; Molecular Docking Appraisal of Pleurotus Ostreatus Phytochemicals as Potential Inhibitors of PI3K/Akt Pathway for Breast Cancer Treatment - Magdalene Eno Effiong, Mercy Bella-Omunagbe, Israel Sunmola Afolabi, Shalom Nwodo Chinedu, 2025, n. d.). Vincristine, while capable of upregulating PTEN to some extent, did not elicit the same magnitude of downstream pathway suppression, possibly because its action occurs post-translationally rather than *via* modulation of upstream phosphoinositide dynamics.

Furthermore, the *POEE*-treated groups demonstrated significant activation of both intrinsic and extrinsic apoptotic pathways, as evidenced by marked upregulation of Cyt-C, Caspase-9, Caspase-3, and pro-apoptotic BAX/BAD proteins. These findings suggest that mitochondrial outer membrane permeabilization (MOMP) and caspase cascade activation were critical components of *POEE*-induced cell death (Dho et al., 2025). The concurrent downregulation of anti-apoptotic BCl-2 further supports this mechanism, tipping the balance toward apoptosis in treated tumors (Qian et al., 2022). Notably, the vincristine-only group displayed weaker activation of intrinsic apoptosis (Cyt-C and Caspase-9), consistent with its primary cytotoxic mechanism involving microtubule depolymerization and mitotic arrest rather than mitochondrial destabilization. POEE’s capacity to modulate both BCl-2 family dynamics and caspase activation underscores its potential to bypass common resistance mechanisms in triple-negative and hormone receptor-negative breast cancers, which often exhibit high BCl-2 expression and impaired apoptosis.

The regulation of BRCA1 and BRCA2 further highlights *POEE*’s contribution to genomic stability. BRCA1 and BRCA2 are essential for the repair of DNA double-strand breaks *via* homologous recombination (Xu et al., 2025; Zong et al., 2025). Their upregulation suggests that *POEE* may enhance DNA repair fidelity, mitigating the mutagenic burden imposed by carcinogens like DMBA and NMU. This is especially relevant in hormone receptor-negative breast cancers, which often harbor BRCA deficiencies (Armstrong et al., 2019; Arun et al., 2024; Choi et al., 2023). The additive effect observed in the *POEE* + vincristine combination, particularly on BRCA1 expression, supports the hypothesis that *POEE* could restore genomic surveillance pathways in otherwise repair-deficient tumor contexts.

Futhermore, *POEE*’s modulation of NFκB, a master transcriptional regulator of inflammation, cell survival, and immune evasion. In the cancer model, NFκB was markedly elevated, consistent with its known role in promoting tumor progression and chemoresistance. *POEE* sharply reduced NFκB expression, thereby removing a critical block to apoptosis and possibly impairing the inflammatory microenvironment that fuels tumorigenesis (Mao et al., 2025; Tripathi et al., 2025). However, when combined with vincristine, NFκB suppression was partially reversed, indicating potential antagonism, perhaps through stress-activated compensatory signalling in response to dual drug exposure.

### 4.3 Comparative effects of *PoEE* and vincristine on DMBA-NMU induced breast cancer

The comparative evaluation of *P. ostreatus* ethanolic extract (*PoEE*) and Vincristine (Vin) reveals that while both agents target oncogenic progression, *PoEE* functions as a comprehensive multi-target regulator, whereas Vin exhibits a more selective and paradoxically compensatory profile. *PoEE* achieved balanced suppression of the PI3K/Akt/mTOR axis, reducing PI3K, Akt, and mTOR by 69%, 58%, and 73% respectively, avoiding the significant compensatory upregulation of Akt, MAPK/p38, and MDM2 observed with Vin monotherapy. Furthermore, *PoEE* demonstrated superior efficacy in restoring essential tumor suppressors and DNA repair machinery, significantly upregulating p27, FOXO, PTEN, and BRCA1/2, which remained largely unresponsive to Vin treatment. While Vin was a potent inducer of Caspase 3, *PoEE* provided a more holistic reactivation of the apoptotic machinery by restoring BAD, BAX, CYT-C, and Caspase 8 while more effectively attenuating Bcl-2. Additionally, *PoEE* successfully reprogrammed the tumor microenvironment by suppressing NFkB and restoring GATA3, whereas the Vin combination paradoxically triggered pro-inflammatory NFkB signaling.

### 4.4 Antagonistic drug (vincristine)-Herb(*PoEE*) interaction

The investigation into the combination of *P. ostreatus* ethanolic extract (*PoEE*) and vincristine (Vin) revealed a complex pharmacological profile characterized by selective molecular synergy alongside marked physiological toxicity. Although the combination enhanced BRCA1, BRCA2, and GATA3 expression and suppressed Akt and MDM2, these molecular improvements did not translate into improved survival, as evidenced by increased tumor burden and mortality. This paradox is consistent with the broader concept of pharmacological antagonism described in traditional medicine systems, such as the “Eighteen Incompatibilities,” where certain herbal pairs, despite individual therapeutic value, can generate adverse outcomes when co-administered due to ratio-dependent, duration-dependent, or mechanistic conflicts (Truong et al., 2025). Contemporary pharmacological literature further supports that herb–drug interactions may occur through pharmacokinetic mechanisms (e.g., modulation of CYP450 enzymes, P-glycoprotein transport, altered drug clearance) or pharmacodynamic mechanisms (e.g., opposing or dysregulated signaling effects) (Czigle et al., 2023; Rosenkranz et al., 2012). In the present model, the combination was less effective than *PoEE* monotherapy in suppressing the PI3K/mTOR axis and failed to adequately restore FOXO, p27, and GSK3β, suggesting pathway-level antagonism. More critically, the marked upregulation of NFκB alongside near-complete loss of BAD and Caspase-8 indicates disruption of upstream apoptotic control and possible compensatory inflammatory activation. Such signaling imbalance may reflect off-target pharmacodynamic interference or altered vincristine disposition induced by bioactive constituents of *PoEE*, ultimately amplifying systemic stress. These findings align with emerging evidence that multi-component natural products can both potentiate and impair chemotherapeutic efficacy depending on context, dosage, and molecular target engagement (Jung and Cheon, 2024). Therefore, the observed toxicity signal underscores the need for rigorous mechanistic evaluation, including pharmacokinetic profiling, inflammatory mediator assessment, and dose-optimization studies, to decipher whether the adverse outcomes arise from pathway antagonism, toxic metabolite generation, altered drug metabolism, or maladaptive immune activation before clinical translation can be considered.

## 5 Conclusion

This study demonstrates the preclinical efficacy of *P. ostreatus* ethanolic extract (*PoEE*), highlighting its role as a natural candidate for further investigation into its mechanistic effects against breast cancer. *PoEE* effectively restored endocrine balance by preserving ovarian steroidogenesis and normalizing key hormonal regulators, thereby counteracting endocrine disruption associated with tumorigenesis. In parallel, *PoEE* inhibited the PI3K/Akt/mTOR signaling axis, a central pathway driving proliferation, survival, and therapy resistance, suggesting its potential to overcome aggressive and treatment-resistant breast cancer subtypes. Furthermore, by maintaining oxidative homeostasis and reducing redox imbalance, *PoEE* addressed a critical driver of DNA damage and malignant progression. These findings reveal the anti-cancer mechanism of *PoEE* through hormonal, molecular, and oxidative regulation, making it a strong candidate for development as a chemo-preventive and therapeutic agent, with particular relevance for hormone receptor–negative breast cancers, where effective interventions remain limited. Combining *PoEE* with vincristine reveals both synergistic and antagonistic interactions, highlighting the complexity of natural–synthetic therapeutic crosstalk. Future studies integrating pharmacometabolic modeling and molecular docking with *in-vivo* dose-response data will be vital to refine combination strategies and enhance clinical translation of mushroom-derived anticancer agents.

## Data Availability

The datasets presented in this study can be found in online repositories. The names of the repository/repositories and accession number(s) can be found in the article/Supplementary Material.

## References

[B112] Adám-Vizi V. Seregi A. (1982). Receptor independent stimulatory effect of noradrenaline on Na,K-ATPase in rat brain homogenate. Role of lipid peroxidation. Biochem. Pharmacol. 31 (13), 2231–2236. 10.1016/0006-2952(82)90106-x 6127081

[B1] Adedara I. A. Subair T. I. Ego V. C. Oyediran O. Farombi E. O. (2017). Chemoprotective role of quercetin in manganese-induced toxicity along the brain-pituitary-testicular axis in rats. Chemico-Biological Interact. 263, 88–98. 10.1016/j.cbi.2016.12.019 28040552

[B2] Adefisan A. O. Owumi S. E. Soetan K. O. Adaramoye O. A. (2022). Chloroform extract of *Calliandra portoricensis* inhibits tumourigenic effect of *N* -methyl- *N* -nitrosourea and benzo(a)pyrene in breast experimental cancer. Drug Chem. Toxicol. 45 (6), 2424–2438. 10.1080/01480545.2021.1957556 34325589

[B3] Adefisan-Adeoye A. O. Ayanbanjo O. O. Adeoye T. D. Jayesimi T. E. Unuofin J. O. Lebelo S. L. (2025). Bisdemethoxycurcumin chemoprevents 7,12-dimethylbenz(a)anthracene-induced mammary toxicity *via* modulation of oxidative processes. Sci. Rep. 15 (1), 9170. 10.1038/s41598-025-94168-x 40097731 PMC11914581

[B109] Afolabi I. S. Adigun A. J. Garuba P. A. Ahuekwe E. F. Odutayo O. E. Adeyemi A. O. (2023). Enterococcus faecalis-aided fermentation to facilitate edible properties and bioactive transformation of underutilized cyathea dregei leaves. Fermentation 9 (8), 707. 10.3390/fermentation9080707

[B4] Aguilar Uscanga B. R. Cavazos Garduño A. Solís Pacheco J. R. Sandoval Garcia F. Navarro Hernández R. E. Serrano Niño J. (2020). *In-vivo* assessment of the protection of β-glucans of Pleurotus ostreatus against oxidative stress caused by acrylamide intake (part II). Nutr. Hosp. 37, 1028–1032. 10.20960/nh.03117 32960618

[B110] Akhouri V. Kumar A. Kumari M. (2020). Antitumour Property of pterocarpus santalinus seeds against dmba-induced breast cancer in rats. Breast Cancer (Auckl) 14, 1178223420951193. 10.1177/1178223420951193 32913391 PMC7444153

[B5] Anifowose L. O. Paimo O. K. Adegboyega F. N. Ogunyemi O. M. Akano R. O. Hammad S. F. (2023). Molecular docking appraisal of Dysphania ambrosioides phytochemicals as potential inhibitor of a key triple-negative breast cancer driver gene. Silico Pharmacol. 11 (1), 15. 10.1007/s40203-023-00152-6 37323538 PMC10267046

[B6] Armstrong N. Ryder S. Forbes C. Ross J. Quek R. G. (2019). A systematic review of the international prevalence of BRCA mutation in breast cancer. Clin. Epidemiol. 11, 543–561. 10.2147/CLEP.S206949 31372057 PMC6628947

[B7] Arun B. Couch F. J. Abraham J. Tung N. Fasching P. A. (2024). BRCA-mutated breast cancer: the unmet need, challenges and therapeutic benefits of genetic testing. Br. J. Cancer 131 (9), 1400–1414. 10.1038/s41416-024-02827-z 39215191 PMC11519381

[B8] Bergez-Hernández F. Irigoyen-Arredondo M. Martínez-Camberos A. (2024). A systematic review of mechanisms of PTEN gene down-regulation mediated by miRNA in prostate cancer. Heliyon 10 (15), e34950. 10.1016/j.heliyon.2024.e34950 39144981 PMC11320309

[B9] Beutler E. Duron O. Kelly B. M. (1963). Improved method for the determination of blood glutathione. J. Lab. Clin. Med. 61, 882–888. 13967893

[B10] Bradford M. M. (1976). A rapid and sensitive method for the quantitation of microgram quantities of protein utilizing the principle of protein-dye binding. Anal. Biochem. 72 (1–2), 248–254. 10.1016/0003-2697(76)90527-3 942051

[B11] Chandimali N. Bak S. G. Park E. H. Lim H.-J. Won Y.-S. Kim E.-K. (2025). Free radicals and their impact on health and antioxidant defenses: a review. Cell Death Discov. 11 (1), 19. 10.1038/s41420-024-02278-8 39856066 PMC11760946

[B12] Choi E. Mun G. Lee J. Lee H. Cho J. Lee Y.-S. (2023). BRCA1 deficiency in triple-negative breast cancer: protein stability as a basis for therapy. Biomed. Pharmacother. 158, 114090. 10.1016/j.biopha.2022.114090 36493696

[B13] Choi H. J. Choi J. W. Park S. J. Lee S. H. Hwang J. H. Park Y. (2025). Anti-inflammatory mechanisms of *Pleurotus citrinopileatus*: inhibition of MAPK and NF-κB signaling pathways, and activation of ROS/PI3K/Nrf2/HO-1 signaling pathway in LPS-stimulated RAW264.7 cells. J. Microbiol. Biotechnol. 35, e2501012. 10.4014/jmb.2501.01012 40374537 PMC12099615

[B113] Chrisnasari R. Ewing T. A. Hilgers R. Van Berke W. J. H. Vincken J.-P. Hennebelle M. (2024). Versatile ferrous oxidation–xylenol orange assay for high-throughput screening of lipoxygenase activity. Appl. Microbiol. Biotechnol. 108 (1), 266. 10.1007/s00253-024-13095-5 38498184 PMC10948578

[B14] Claiborne A. (1985). “Catalase activity,” in CRC handbook of methods for oxygen radical research, CRC press. Editor Greenwald R. A. (Boca Raton: References—Scientific Research Publishing), 283–284.

[B15] Czigle S. Nagy M. Mladěnka P. Tóth J. OEMONOM. (2023). Pharmacokinetic and pharmacodynamic herb-drug interactions—Part I. Herbal medicines of the central nervous system. PeerJ 11, e16149. 10.7717/peerj.16149 38025741 PMC10656908

[B16] Dania O. E. Dokunmu T. M. Adegboye B. E. Adeyemi A. O. Chibuzor F. C. Iweala E. E. J. (2024). Pro-estrogenic and anti-inflammatory effects of corchorus olitorius and *Amaranthus hybridus* leaves in DMBA-induced breast cancer. Phytomedicine Plus 4 (2), 100567. 10.1016/j.phyplu.2024.100567

[B17] Dho S. H. Cho M. Woo W. Jeong S. Kim L. K. (2025). Caspases as master regulators of programmed cell death: apoptosis, pyroptosis and beyond. Exp. Mol. Med. 57 (6), 1121–1132. 10.1038/s12276-025-01470-9 40555741 PMC12229594

[B18] Dong R. Wang J. Guan R. Sun J. Jin P. Shen J. (2025). Role of oxidative stress in the occurrence, development, and treatment of breast cancer. Antioxidants 14 (1), 104. 10.3390/antiox14010104 39857438 PMC11760893

[B19] Donovan J. Deng Z. Bian F. Shukla S. Gomez-Arroyo J. Shi D. (2023). Improving anti-tumor efficacy of low-dose vincristine in rhabdomyosarcoma *via* the combination therapy with FOXM1 inhibitor RCM1. Front. Oncol. 13, 1112859. 10.3389/fonc.2023.1112859 36816948 PMC9933126

[B20] Ebrahimi A. Atashi A. Soleimani M. Mashhadikhan M. Barahimi A. Kaviani S. (2018). Comparison of anticancer effect of *Pleurotus ostreatus* extract with doxorubicin hydrochloride alone and plus thermotherapy on erythroleukemia cell line. J. Complem. Integr. Med. 15 (2), 20160136. 10.1515/jcim-2016-0136 29257758

[B21] Effiong M. E. Bella-Omunagbe M. Afolabi I. S. Chinedu S. N. (2024a). *In silico* evaluation of potential breast cancer receptor antagonists from GC-MS and HPLC identified compounds in *Pleurotus ostreatus* extracts. RSC Adv. 14 (33), 23744–23771. 10.1039/D4RA03832K 39131188 PMC11310660

[B22] Effiong M. E. Umeokwochi C. P. Afolabi I. S. Chinedu S. N. (2024b). Comparative antioxidant activity and phytochemical content of five extracts of *Pleurotus ostreatus* (oyster mushroom). Sci. Rep. 14 (1), 3794. 10.1038/s41598-024-54201-x 38361132 PMC10869810

[B23] Effiong M. E. Bella-Omunagbe M. Afolabi I. S. Chinedu S. N. (2025a). Molecular docking appraisal of *Pleurotus ostreatus* phytochemicals as potential inhibitors of PI3K/Akt pathway for breast cancer treatment. Bioinforma. Biol. Insights 19, 11779322251316864. 39906062 10.1177/11779322251316864 PMC11792010

[B24] Effiong M. E. Chinedu S. N. Afolabi I. S. Ezike K. N. Oguntebi E. E. Abdul O. A. (2025b). Age-specific patterns of breast cancer in Nigerian women unraveled through histological analysis. Sci. Rep. 16, 128. 10.1038/s41598-025-28685-0 41318755 PMC12764456

[B25] Effiong M. E. Cynthia I. Bella-Omunagbe M. Afolabi I. S. Chinedu S. N. (2025c). Microbiome dynamics in breast cancer: mechanisms, therapeutic impacts and research gaps. Crit. Rev. Oncology/Hematology 215, 104879. 10.1016/j.critrevonc.2025.104879 40783076

[B27] Elhusseiny S. M. El-Mahdy T. S. Awad M. F. Elleboudy N. S. Farag M. M. S. Yassein M. A. (2021). Proteome analysis and *in vitro* antiviral, anticancer and antioxidant capacities of the aqueous extracts of *Lentinula edodes* and *Pleurotus ostreatus* edible mushrooms. Molecules 26 (15), 4623. 10.3390/molecules26154623 34361776 PMC8348442

[B28] Ellman G. L. (1959). Tissue sulfhydryl groups. Archives Biochem. Biophysics 82 (1), 70–77. 10.1016/0003-9861(59)90090-6 13650640

[B29] Fukugami S. Yamasaki M. Kokushi E. Uno S. (2025). Influence of CYP1A and AhR modulation on polycyclic aromatic hydrocarbon-induced developmental defects in Japanese medaka. Aquat. Toxicol. 280, 107267. 10.1016/j.aquatox.2025.107267 39933340

[B30] Ganesan K. Xu B. (2018). Anti-obesity effects of medicinal and edible mushrooms. Molecules 23 (11), 2880. 10.3390/molecules23112880 30400600 PMC6278646

[B31] Garg P. Ramisetty S. Nair M. Kulkarni P. Horne D. Salgia R. (2025). Strategic advancements in targeting the PI3K/AKT/mTOR pathway for breast cancer therapy. Biochem. Pharmacol. 236, 116850. 10.1016/j.bcp.2025.116850 40049296

[B32] Glaviano A. Foo A. S. C. Lam H. Y. Yap K. C. H. Jacot W. Jones R. H. (2023). PI3K/AKT/mTOR signaling transduction pathway and targeted therapies in cancer. Mol. Cancer 22 (1), 138. 10.1186/s12943-023-01827-6 37596643 PMC10436543

[B33] Green L. C. Wagner D. A. Glogowski J. Skipper P. L. Wishnok J. S. Tannenbaum S. R. (1982). Analysis of nitrate, nitrite, and [15N]nitrate in biological fluids. Anal. Biochem. 126 (1), 131–138. 10.1016/0003-2697(82)90118-X 7181105

[B34] Habig W. H. Pabst M. J. Jakoby W. B. (1974). Glutathione S-transferases. The first enzymatic step in mercapturic acid formation. J. Biol. Chem. 249 (22), 7130–7139. 4436300

[B35] Hadwan M. H. (2018). Simple spectrophotometric assay for measuring catalase activity in biological tissues. BMC Biochem. 19 (1), 7. 10.1186/s12858-018-0097-5 30075706 PMC6091033

[B36] Haque Md. A. Islam Md. A. U. (2019). Pleurotus highking mushroom induces apoptosis by altering the balance of proapoptotic and antiapoptotic genes in breast cancer cells and inhibits tumor sphere Formation. Medicina 55 (11), 716. 10.3390/medicina55110716 31661925 PMC6915458

[B37] Hassan A. Aubel C. (2025). The PI3K/Akt/mTOR signaling pathway in triple-negative breast cancer: a resistance pathway and a prime target for targeted therapies. Cancers 17 (13), 2232. 10.3390/cancers17132232 40647529 PMC12248877

[B38] Iheagwam F. N. Israel E. N. Kayode K. O. De Campos O. C. Ogunlana O. O. Chinedu S. N. (2019a). GC-MS analysis and inhibitory evaluation of *Terminalia catappa* leaf extracts on major enzymes linked to diabetes. Evidence-Based Complementary Altern. Med. 2019 (1), 6316231. 10.1155/2019/6316231 31662777 PMC6748200

[B39] Iheagwam F. N. Ogunlana O. O. Chinedu S. N. (2019b). Model optimization and *in silico* analysis of potential dipeptidyl peptidase IV antagonists from GC-MS identified compounds in Nauclea latifolia leaf extracts. Int. J. Mol. Sci. 20 (23), 5913. 10.3390/ijms20235913 31775302 PMC6929178

[B40] Ikram A. Ibrahim N. A. Arshad M. T. Fatima A. Taseer A. A. Hussain M. F. (2025). Mushroom bioactive molecules as anticancerous agents: an overview. Food Sci. Nutr. 13 (7), e70580. 10.1002/fsn3.70580 40661813 PMC12256995

[B41] Iqbal T. Sohaib M. Iqbal S. Rehman H. (2024). Exploring therapeutic potential of *Pleurotus ostreatus* and *Agaricus bisporus* mushrooms against hyperlipidemia and oxidative stress using animal model. Foods 13 (5), 709. 10.3390/foods13050709 38472823 PMC10930387

[B42] Ito T. Urushima H. Sakaue M. Yukawa S. Honda H. Hirai K. (2014). Reduction of adverse effects by a mushroom product, active Hexose Correlated Compound (AHCC) in patients with advanced cancer during chemotherapy—the significance of the levels of HHV-6 DNA in saliva as a surrogate biomarker during chemotherapy. Nutr. Cancer 66 (3), 377–382. 10.1080/01635581.2014.884232 24611562

[B43] Jaeck S. Depuydt C. Bernard V. Ammar O. Hocké C. Carrière J. (2025). How to preserve fertility in reproductive-age women with cancer. J. Clin. Med. 14 (6), 1912. 10.3390/jcm14061912 40142718 PMC11942802

[B44] Jayaprakash B. Suresh A. R. Thiruvengadam R. Alharbi N. S. Kadaikunnan S. Sankaran S. (2024). Evaluation of oyster mushroom (*Pleurotus ostreatus*)-derived anthraquinone on the induction of apoptosis and suppression of MMP-2 and MMP-9 expression in breast cancer cells. Int. J. Med. Sci. 21 (6), 1016–1026. 10.7150/ijms.93334 38774755 PMC11103395

[B45] Jayasuriya W. J. A. B. N. Handunnetti S. M. Wanigatunge C. A. Fernando G. H. Abeytunga D. T. U. Suresh T. S. (2020). Anti‐inflammatory activity of *Pleurotus ostreatus*, a culinary medicinal mushroom, in Wistar rats. Evidence-Based Complementary Altern. Med. 2020 (1), 6845383. 10.1155/2020/6845383 32215044 PMC7077046

[B46] Jedinak A. Dudhgaonkar S. Wu Q. Simon J. Sliva D. (2011). Anti-inflammatory activity of edible oyster mushroom is mediated through the inhibition of NF-κB and AP-1 signaling. Nutr. J. 10 (1), 52. 10.1186/1475-2891-10-52 21575254 PMC3120742

[B47] Jung T. Cheon C. (2024). Synergistic and additive effects of herbal medicines in combination with chemotherapeutics: a scoping review. Integr. Cancer Ther. 23, 15347354241259416. 10.1177/15347354241259416 38867515 PMC11179546

[B48] Khattar E. Tergaonkar V. (2017). Transcriptional regulation of telomerase reverse transcriptase (TERT) by MYC. Front. Cell Dev. Biol. 5, 1. 10.3389/fcell.2017.00001 28184371 PMC5266692

[B49] Kielkopf C. L. Bauer W. Urbatsch I. L. (2020). Bradford assay for determining protein concentration. Cold Spring Harb. Protoc. 2020 (4), pdb.prot102269. 10.1101/pdb.prot102269 32238597

[B50] Kim M. E. Lee J. S. (2025). Advances in the regulation of inflammatory mediators in nitric oxide synthase: implications for disease modulation and therapeutic approaches. Int. J. Mol. Sci. 26 (3), 1204. 10.3390/ijms26031204 39940974 PMC11818275

[B51] Krenacs L. Krenacs T. Stelkovics E. Raffeld M. (2010). Heat-induced antigen retrieval for immunohistochemical reactions in routinely processed paraffin sections. Methods Mol. Biol. 588, 103–119. 10.1007/978-1-59745-324-0_14 20012825 PMC7604828

[B52] Krishnamoorthy D. Sankaran M. (2016). Modulatory effect of *Pleurotus ostreatus* on oxidant/antioxidant status in 7, 12-dimethylbenz (a) anthracene induced mammary carcinoma in experimental rats—A dose-response study. J. Cancer Res. Ther. 12 (1), 386–394. 10.4103/0973-1482.148691 27072268

[B53] Lawrence R. A. Sunde R. A. Schwartz G. L. Hoekstra W. G. (1974). Glutathione peroxidase activity in rat lens and other tissues in relation to dietary selenium intake. Exp. Eye Res. 18 (6), 563–569. 10.1016/0014-4835(74)90062-1 4852169

[B54] Liu B. Peng Z. Zhang H. Zhang N. Liu Z. Xia Z. (2025). Regulation of cellular senescence in tumor progression and therapeutic targeting: mechanisms and pathways. Mol. Cancer 24 (1), 106. 10.1186/s12943-025-02284-z 40170077 PMC11963325

[B55] López‐Tofiño Y. De Sosa F. Vera G. López‐Gómez L. Herradón E. López‐Miranda V. (2024). Effects of vincristine and monosodium glutamate on gastrointestinal motility and visceral sensitivity. Neurogastroenterol. Motil. 36 (1), e14704. 10.1111/nmo.14704 37964110

[B56] Ma Z. Kim Y. Howard E. Feng X. Kosanke S. Yang S. (2018). DMBA promotes ErbB2-mediated carcinogenesis *via* ErbB2 and estrogen receptor pathway activation and genomic instability. Oncol. Rep. 40, 1632–1640. 10.3892/or.2018.6545 30015966 PMC6072406

[B57] Ma N. Wang Y. Li X. Xu M. Tan D. (2025). Reactive oxygen species in cancer: mechanistic insights and therapeutic innovations. Cell Stress Chaperones 30 (5), 100108. 10.1016/j.cstres.2025.100108 40769273 PMC12398932

[B58] MacArthur Clark J. A. Sun D. (2020). Guidelines for the ethical review of laboratory animal welfare people’s Republic of China National Standard GB/T 35892‐2018 [issued 6 February 2018 effective from 1 September 2018]. Animal Models Exp. Med. 3 (1), 103–113. 10.1002/ame2.12111 32318667 PMC7167230

[B59] Maiti S. Mallick S. K. Bhutia S. K. Behera B. Mandal M. Maiti T. K. (2011). Antitumor effect of culinary-medicinal oyster mushroom, *Pleurotus ostreatus* (Jacq.: Fr.) P. Kumm., derived protein fraction on tumor-bearing mice models. Int. J. Med. Mushrooms 13 (5), 427–440. 10.1615/IntJMedMushr.v13.i5.20 22324408

[B60] Maity B. Sheff D. Fisher R. A. (2013). Immunostaining. Methods Cell Biol. 113, 81–105. 10.1016/B978-0-12-407239-8.00005-7 23317899

[B61] Mao H. Zhao X. Sun S. (2025). NF-κB in inflammation and cancer. Cell. Mol. Immunol. 22 (8), 811–839. 10.1038/s41423-025-01310-w 40562870 PMC12310982

[B62] Mishra V. Tomar S. Yadav P. Vishwakarma S. Singh M. P. (2022). Elemental analysis, phytochemical screening and evaluation of antioxidant, antibacterial and anticancer activity of *Pleurotus ostreatus* through *in vitro* and *in silico* approaches. Metabolites 12 (9), 821. 10.3390/metabo12090821 36144225 PMC9502197

[B63] Misra H. P. Fridovich I. (1972). The role of superoxide anion in the autoxidation of epinephrine and a simple assay for superoxide dismutase. J. Biol. Chem. 247 (10), 3170–3175. 4623845

[B64] Molecular Docking (2025). Molecular docking appraisal of *Pleurotus ostreatus* phytochemicals as potential inhibitors of PI3K/Akt pathway for breast cancer treatment—Magdalene Eno Effiong, Mercy Bella-Omunagbe, Israel Sunmola Afolabi, Shalom Nwodo Chinedu. (n.d.). 10.1177/11779322251316864 PMC11792010 39906062

[B65] Moreira D. C. (2022). RGBradford: accurate measurement of protein concentration using a smartphone camera and the blue to green intensity ratio. Anal. Biochem. 655, 114839. 10.1016/j.ab.2022.114839 35987416

[B66] Moyen Uddin Pk M. O’Sullivan J. Sayful Islam M. Shahangir Biswas M. Arbia L. Pervin R. (2024). Investigating the anticancer effects of *Pleurotus ostreatus* polysaccharide on G0/G1 cell cycle arrest and apoptosis in ehrlich ascites carcinoma cells. Chem. Biodivers. 21 (9), e202400897. 10.1002/cbdv.202400897 38970566

[B67] Naguib Y. M. Azmy R. M. Samaka R. M. Salem M. F. (2014). *Pleurotus ostreatus* opposes mitochondrial dysfunction and oxidative stress in acetaminophen-induced hepato-renal injury. BMC Complementary Altern. Med. 14 (1), 494. 10.1186/1472-6882-14-494 25510860 PMC4301462

[B68] Nair A. B. Jacob S. (2016). A simple practice guide for dose conversion between animals and human. J. Basic Clin. Pharm. 7 (2), 27–31. 10.4103/0976-0105.177703 27057123 PMC4804402

[B69] Ni Y. Wang W. Xu Y. Zhang W. (2025). A study on the impact of polycyclic aromatic hydrocarbons (PAHs) on the risk of liver disease in middle-aged and older adults people based on the CHARLS database. Ecotoxicol. Environ. Saf. 300, 118493. 10.1016/j.ecoenv.2025.118493 40494191

[B70] Nwozo O. S. Effiong E. M. Aja P. M. Awuchi C. G. (2023). Antioxidant, phytochemical, and therapeutic properties of medicinal plants: a review. Int. J. Food Prop. 26 (1), 359–388. 10.1080/10942912.2022.2157425

[B71] Nwozol S. O. Effiong M. E. (2019). Phytochemical composition, mineral content and antioxidant activities of the methanol extract of *Curcuma longa* and *Viscum album* . J. Food Pharm. Sci. 3 (1), 45–54. 10.22146/jfps.708

[B72] Owumi S. E. Nwozo S. O. Effiong M. E. Najophe E. S. (2020). Gallic acid and omega-3 fatty acids decrease inflammatory and oxidative stress in manganese-treated rats. Exp. Biol. Med. 245 (9), 835–844. 10.1177/1535370220917643 32252555 PMC7273889

[B73] Owumi S. E. Umez A. O. Arunsi U. Irozuru C. E. (2023). Dietary aflatoxin B1 and antimalarial-a lumefantrine/artesunate-therapy perturbs male rat reproductive function *via* pro-inflammatory and oxidative mechanisms. Sci. Rep. 13 (1), 12172. 10.1038/s41598-023-39455-1 37500724 PMC10374580

[B74] Pellarin I. Dall’Acqua A. Favero A. Segatto I. Rossi V. Crestan N. (2025). Cyclin-dependent protein kinases and cell cycle regulation in biology and disease. Signal Transduct. Target. Ther. 10 (1), 11. 10.1038/s41392-024-02080-z 39800748 PMC11734941

[B75] Qian S. Wei Z. Yang W. Huang J. Yang Y. Wang J. (2022). The role of BCL-2 family proteins in regulating apoptosis and cancer therapy. Front. Oncol. 12, 985363. 10.3389/fonc.2022.985363 36313628 PMC9597512

[B76] Qiang M. Chen Z. Liu H. Dong J. Gong K. Zhang X. (2025). Targeting the PI3K/AKT/mTOR pathway in lung cancer: mechanisms and therapeutic targeting. Front. Pharmacol. 16, 1516583. 10.3389/fphar.2025.1516583 40041495 PMC11877449

[B115] Rahman M. Asaeda T. Fukahori K. Imamura F. Nohara A. Matsubayashi M. (2023). Hydrogen Peroxide Measurement Can Be Used to Monitor Plant Oxidative Stress Rapidly Using Modified Ferrous Oxidation Xylenol Orange and Titanium Sulfate Assay Correlation Int. J. Plant Biol. 14 (3), 546–557. 10.3390/ijpb14030043

[B77] Robinson N. J. Schiemann W. P. (2022). Telomerase in cancer: function, regulation, and clinical translation. Cancers 14 (3), 808. 10.3390/cancers14030808 35159075 PMC8834434

[B78] Rosenkranz B. Fasinu P. Bouic P. (2012). An overview of the evidence and mechanisms of herb–drug interactions. Front. Pharmacol. 3, 69. 10.3389/fphar.2012.00069 22557968 PMC3339338

[B79] Salemcity A. J. Nwaneri-Chidozie V. O. Adameh E. Eno Effiong M. (2020). Antioxidant and free radical scavenging activities of Newbouldia laevis leaf extracts. Free Radicals Antioxidants 10 (1), 10–15. 10.5530/fra.2020.1.3

[B111] Saunders W. B. (1994). Tietz Textbook of Clinical Chemistry. 2nd Edn. Philadelphia, PA.

[B80] Shi X. Zhao Y. Jiao Y. Shi T. Yang X. (2013). ROS-dependent mitochondria molecular mechanisms underlying antitumor activity of *Pleurotus abalonu*s acidic polysaccharides in human breast cancer MCF-7 cells. PLoS ONE 8 (5), e64266. 10.1371/journal.pone.0064266 23691187 PMC3653930

[B81] Sias F. Zoroddu S. Migheli R. Bagella L. (2025). Untangling the role of MYC in sarcomas and its potential as a promising therapeutic target. Int. J. Mol. Sci. 26 (5), 1973. 10.3390/ijms26051973 40076599 PMC11900228

[B82] Singh A. Saini R. K. Kumar A. Chawla P. Kaushik R. (2025). Mushrooms as nutritional powerhouses: a review of their bioactive compounds, health benefits, and value-added products. Foods 14 (5), 741. 10.3390/foods14050741 40077445 PMC11899115

[B83] Slaoui M. Fiette L. (2011). Histopathology procedures: from tissue sampling to histopathological evaluation. Drug Saf. Eval. 691, 69–82. 10.1007/978-1-60761-849-2_4 20972747

[B84] Soares C. L. R. Wilairatana P. Silva L. R. Moreira P. S. Vilar Barbosa N. M. M. Da Silva P. R. (2023). Biochemical aspects of the inflammatory process: a narrative review. Biomed. Pharmacother. 168, 115764. 10.1016/j.biopha.2023.115764 37897973

[B85] Sreedharan P. L. Kishorkumar M. Neumann E. G. Kurup S. S. (2025). The emerging role of oyster mushrooms as a functional food for complementary cancer therapy. Foods 14 (1), 128. 10.3390/foods14010128 39796417 PMC11719500

[B86] Swiss V. A. Casaccia P. (2010). Cell‐context specific role of the E2F/Rb pathway in development and disease. Glia 58 (4), 377–390. 10.1002/glia.20933 19795505 PMC2882865

[B87] Sztukowski K. E. Yaufman Z. Cook M. R. Aarnes T. K. Husbands B. D. (2024). Vincristine‐induced adverse events related to body weight in dogs treated for lymphoma. J. Veterinary Intern. Med. 38 (3), 1686–1692. 10.1111/jvim.17063 38563346 PMC11099714

[B88] Takahashi T. Honma Y. Miyake T. Adachi K. Takami S. Okada M. (2015). Synergistic combination therapy with cotylenin A and vincristine in multiple myeloma models. Int. J. Oncol. 46 (4), 1801–1809. 10.3892/ijo.2015.2882 25672400

[B89] Tripathi S. Sharma Y. Kumar D. (2025). Unveiling the link between chronic inflammation and cancer. Metab. Open 25, 100347. 10.1016/j.metop.2025.100347 39876904 PMC11772974

[B90] Truong M.-N. Duong-Thi N.-L. Vo T.-T. Le-Thi L.-P. (2025). The antagonistic interactions between traditional medicine herbs and licorice: a non-systematic review. Nat. Product. Commun. 20 (8), 1934578X251366164. 10.1177/1934578X251366164

[B91] Trush M. A. Egner P. A. Kensler T. W. (1994). Myeloperoxidase as a biomarker of skin irritation and inflammation. Food Chem. Toxicol. 32 (2), 143–147. 10.1016/0278-6915(94)90175-9 8132173

[B92] Udobi M. E. Chinedu S. N. Afolabi I. S. Ezike K. N. Ikeji N. C. Farombi E. O. (2025). 1268 age-dependent PI3K/AKT/mTOR signaling dynamics reveal immune microenvironment heterogeneity in Nigerian breast cancer subtypes. Regul. Young Investigator Award Abstr., 31 (1), A1444–A1444. 10.1136/jitc-2025-SITC2025.1268

[B200] Udobi M. E. Chinedu S. N. Afolabi I. S. Ezike K. N. Ikeji N. C. Farombi E. O. (2026). PI3K/Akt/mTOR pathway expression profiling reveals age- and subtype-specific molecular heterogeneity in the Nigerian breast cancer landscape. Front. Oncol. 16, 1766066. 10.3389/fonc.2026.1766066 41889413 PMC13012984

[B93] Varshney R. Kale R. K. (1990). Effects of Calmodulin antagonists on radiation-induced lipid peroxidation in microsomes. Int. J. Radiat. Biol. 58 (5), 733–743. 10.1080/09553009014552121 1977818

[B94] Venturella G. Ferraro V. Cirlincione F. Gargano M. L. (2021). Medicinal mushrooms: bioactive compounds, use, and clinical trials. Int. J. Mol. Sci. 22 (2), 634. 10.3390/ijms22020634 33435246 PMC7826851

[B95] Versari I. Salucci S. Bavelloni A. Battistelli M. Traversari M. Wang A. (2025). The emerging role and clinical significance of PI3K-Akt-mTOR in Rhabdomyosarcoma. Biomolecules 15 (3), 334. 10.3390/biom15030334 40149870 PMC11940244

[B96] Wang Q. Gu R. Olm F. Bèchet N. B. Lindstedt S. (2024). Xylene *versus* isopropanol for paraffin wax processing of lung tissue. Appl. Sci. 14 (5), 1726. 10.3390/app14051726

[B97] Wang X. Huang R. Liu L. Wang X. Zhang X. (2025). Evaluation and preservation of fertility in patients with hematologic malignancies. Cancer Lett. 616, 217569. 10.1016/j.canlet.2025.217569 39983893

[B98] Wanwimolruk S. Prachayasittikul V. (2014). Cytochrome P450 enzyme mediated herbal drug interactions (part 1). EXCLI J. 13, 347–391. 26417265 PMC4463967

[B99] Wolff S. P. (1994). [18] ferrous ion oxidation in presence of ferric ion indicator xylenol orange for measurement of hydroperoxides. Methods Enzym. 233, 182–189. 10.1016/S0076-6879(94)33021-2

[B100] Xiong X. Zheng L.-W. Ding Y. Chen Y.-F. Cai Y.-W. Wang L.-P. (2025). Breast cancer: pathogenesis and treatments. Signal Transduct. Target. Ther. 10 (1), 49. 10.1038/s41392-024-02108-4 39966355 PMC11836418

[B101] Xu Z. Xie H. Song L. Huang Y. Huang J. (2025). BRCA1 and BRCA2 in DNA damage and replication stress response: insights into their functions, mechanisms, and implications for cancer treatment. DNA Repair 150, 103847. 10.1016/j.dnarep.2025.103847 40373656

[B102] Yu Y. Jin H. Lu Q. (2022). Effect of polycyclic aromatic hydrocarbons on immunity. J. Transl. Autoimmun. 5, 100177. 10.1016/j.jtauto.2022.100177 36561540 PMC9763510

[B103] Zafar A. Khatoon S. Khan M. J. Abu J. Naeem A. (2025). Advancements and limitations in traditional anti-cancer therapies: a comprehensive review of surgery, chemotherapy, radiation therapy, and hormonal therapy. Discov. Oncol. 16 (1), 607. 10.1007/s12672-025-02198-8 40272602 PMC12021777

[B104] Zeng Q. Zeng S. Dai X. Ding Y. Huang C. Ruan R. (2024). MDM2 inhibitors in cancer immunotherapy: current status and perspective. Genes Dis. 11 (6), 101279. 10.1016/j.gendis.2024.101279 39263534 PMC11388719

[B105] Zhang X. Bai Y. Wang Y. Wang C. Fu J. Gao L. (2020). Anticancer properties of different solvent extracts of *Cucumis melo* L. seeds and whole fruit and their metabolite profiling using HPLC and GC‐MS. BioMed Res. Int. 2020 (1), 5282949. 10.1155/2020/5282949 32185208 PMC7061113

[B106] Zhou Y. Nakajima R. Shirasawa M. Fikriyanti M. Zhao L. Iwanaga R. (2023). Expanding roles of the E2F-RB-p53 pathway in tumor suppression. Biology 12 (12), 1511. 10.3390/biology12121511 38132337 PMC10740672

[B107] Zhou Y. Shen G. Zhou X. Li J. (2025). Therapeutic potential of tumor-associated neutrophils: dual role and phenotypic plasticity. Signal Transduct. Target. Ther. 10 (1), 178. 10.1038/s41392-025-02242-7 40461514 PMC12134342

[B108] Zong D. Pavani R. Nussenzweig A. (2025). New twist on BRCA1-mediated DNA recombination repair and tumor suppression. Trends Cell Biol. S0962892425001126, 4–12. 10.1016/j.tcb.2025.05.002 40480845 PMC12354295

