# Supplementary material for: Pleurotus ostreatus ethanolic extract exerts anti-cancer effects via PI3K/Akt/mTOR pathway modulation in DMBA-NMU induced breast cancer in female Sprague Dawley rats
Source: Front Pharmacol. 2026 Apr 10;17:1766536. doi: 10.3389/fphar.2026.1766536 (PMC13106606; doi:10.3389/fphar.2026.1766536)
Supplement: Supplementary file 1 [file Supplementaryfile1.pdf]

Supplementary Figures

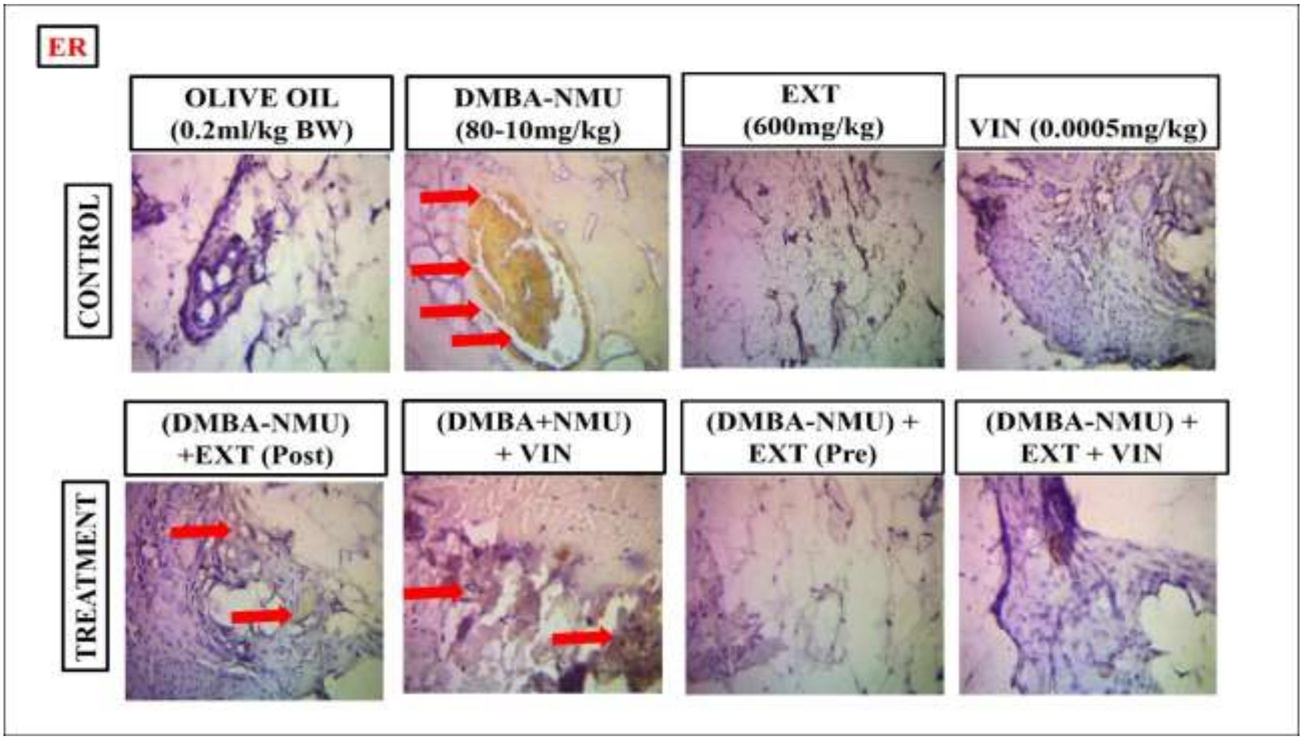

Supplementary Figure 1: Photomicrographs of the effects of *PoEE* on the Expression of ER protein in breast tissues of DMBA-NMU induced BC in Female SD rats. Red arrows in the photomicrographs show the areas of expression.

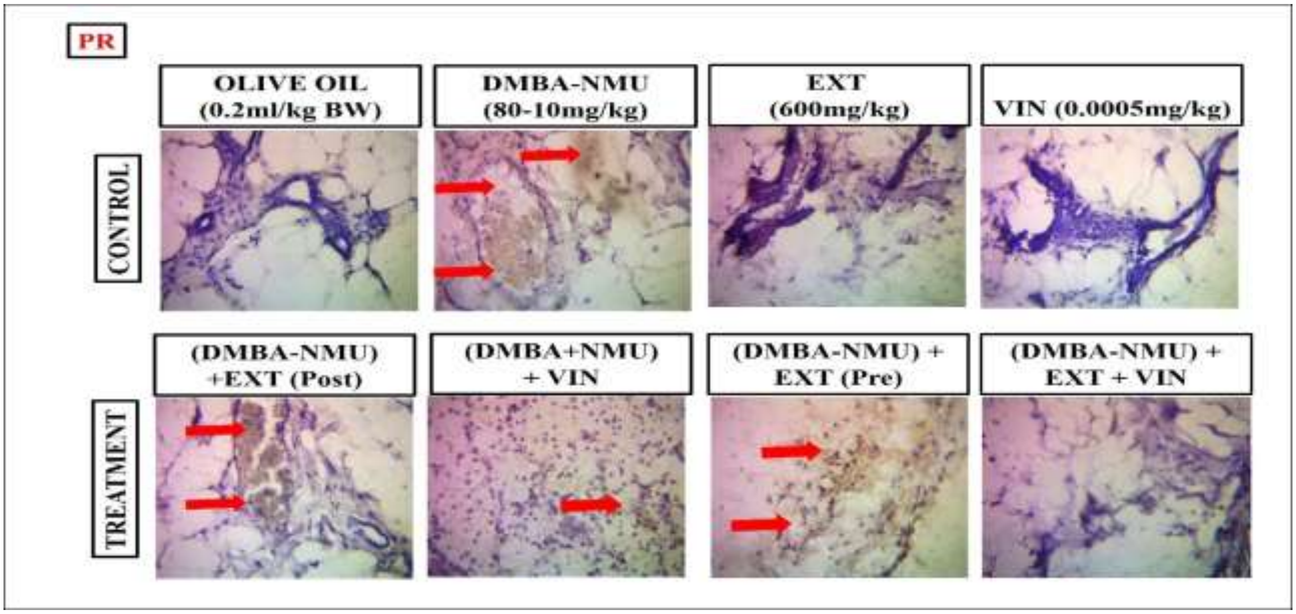

Supplementary Figure 2: Photomicrographs of the effects of *PoEE* on the Expression of PR protein in breast tissues of DMBA-NMU induced BC in Female SD rats. Red arrows in the photomicrographs show the areas of expression.

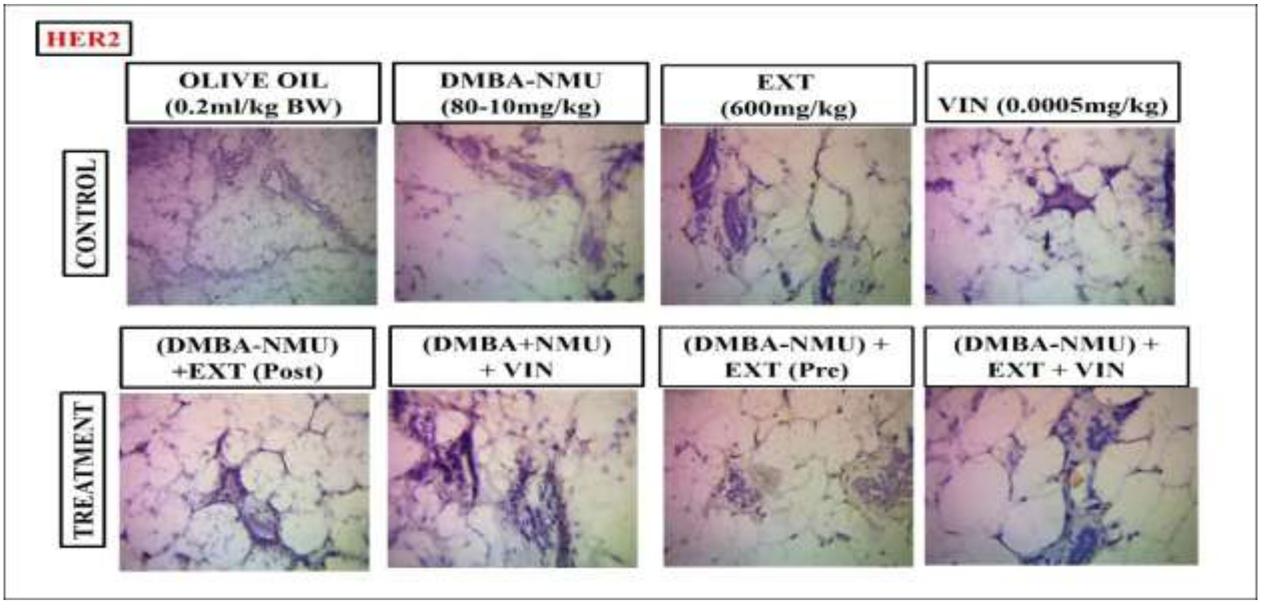

Supplementary Figure 3: Photomicrographs of the effects of *PoEE* on the Expression of EGFR protein in breast tissues of DMBA-NMU induced BC in Female SD rats. Red arrows in the photomicrographs show the areas of expression.

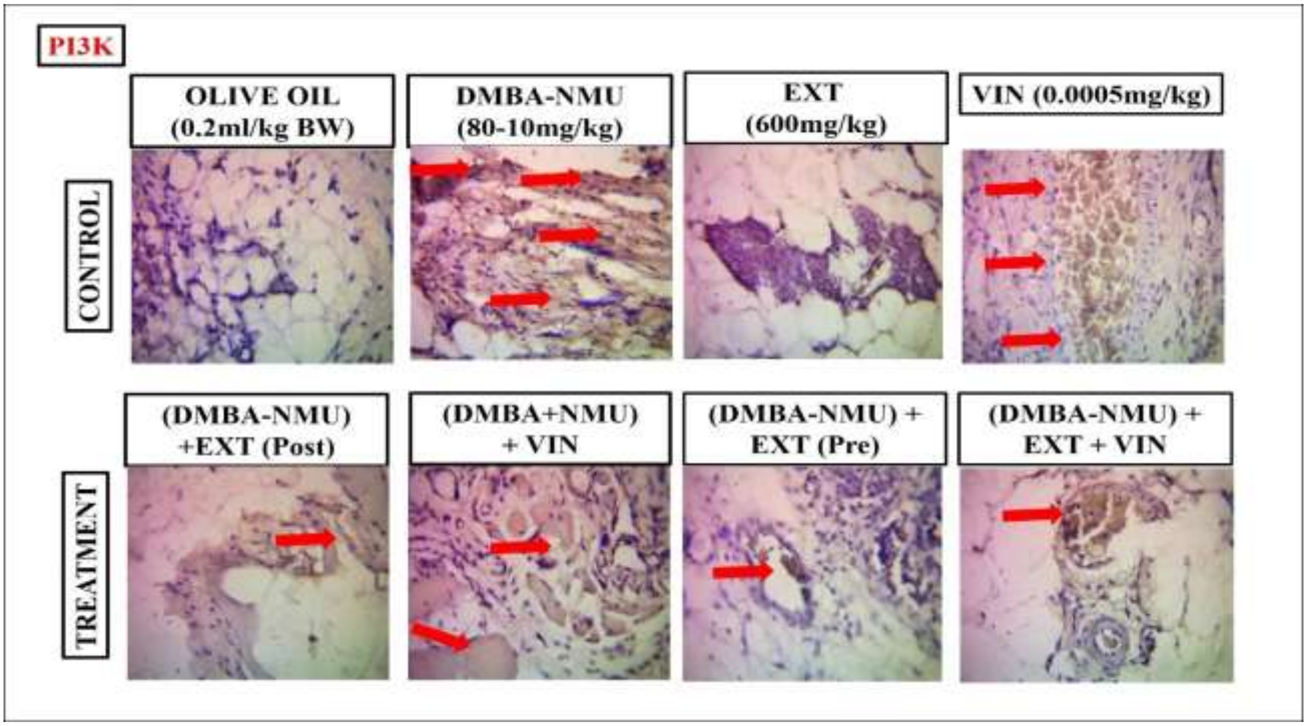

Supplementary Figure 4: Photomicrographs of the effects of *PoEE* on the Expression of EGFR protein in breast tissues of DMBA-NMU induced BC in Female SD rats. Red arrows in the photomicrographs show the areas of expression.

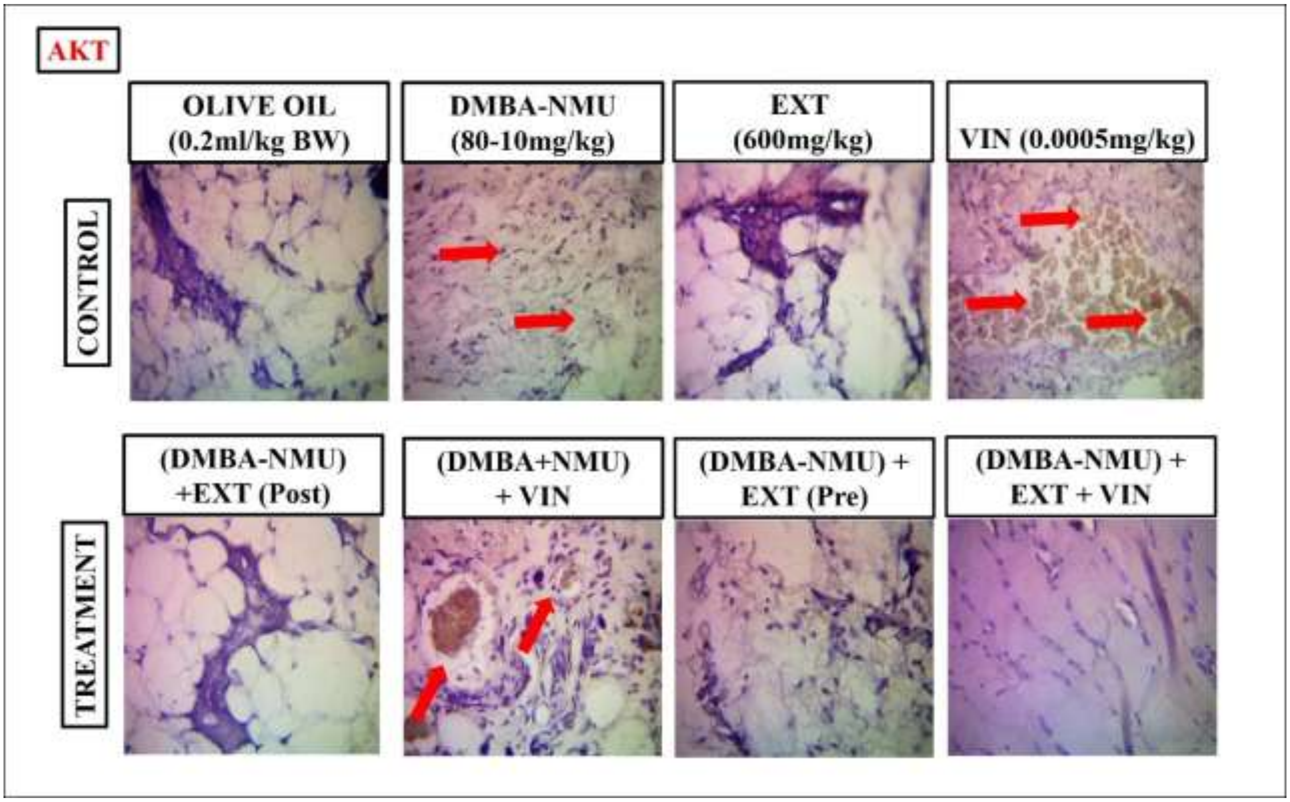

Supplementary Figure 5: Photomicrographs of the effects of *PoEE* on the Expression of AKT protein in breast tissues of DMBA-NMU induced BC in Female SD rats. Red arrows in the photomicrographs show the areas of expression.

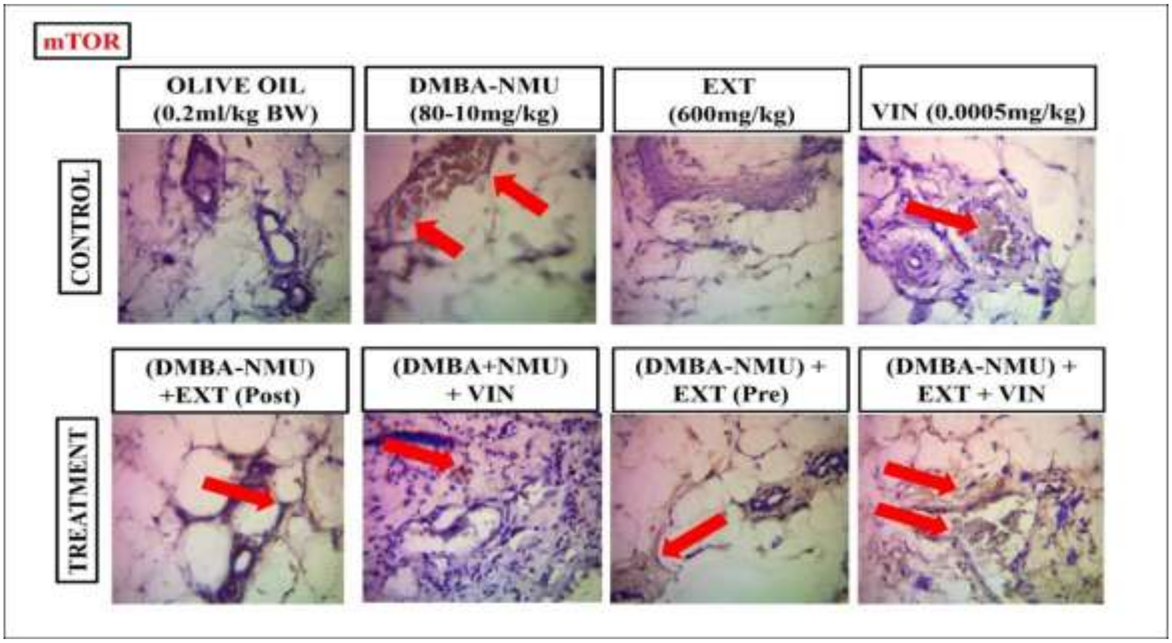

Supplementary Figure 6: Photomicrographs of the effects of *PoEE* on the Expression of mTOR protein in breast tissues of DMBA-NMU induced BC in Female SD rats. Red arrows in the photomicrographs show the areas of expression.

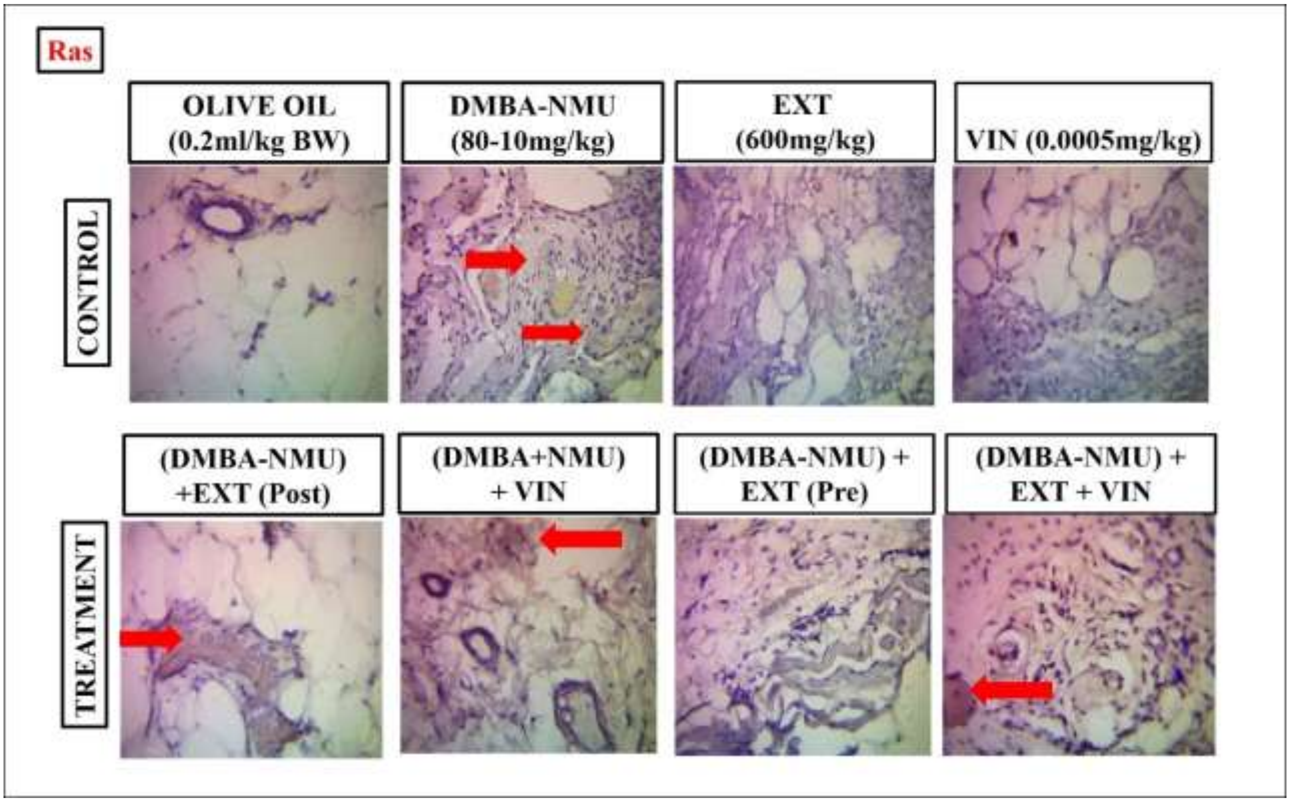

Supplementary Figure 7: Photomicrographs of the effects of *PoEE* on the Expression of Ras protein in breast tissues of DMBA-NMU induced BC in Female SD rats. Red arrows in the photomicrographs show the areas of expression.

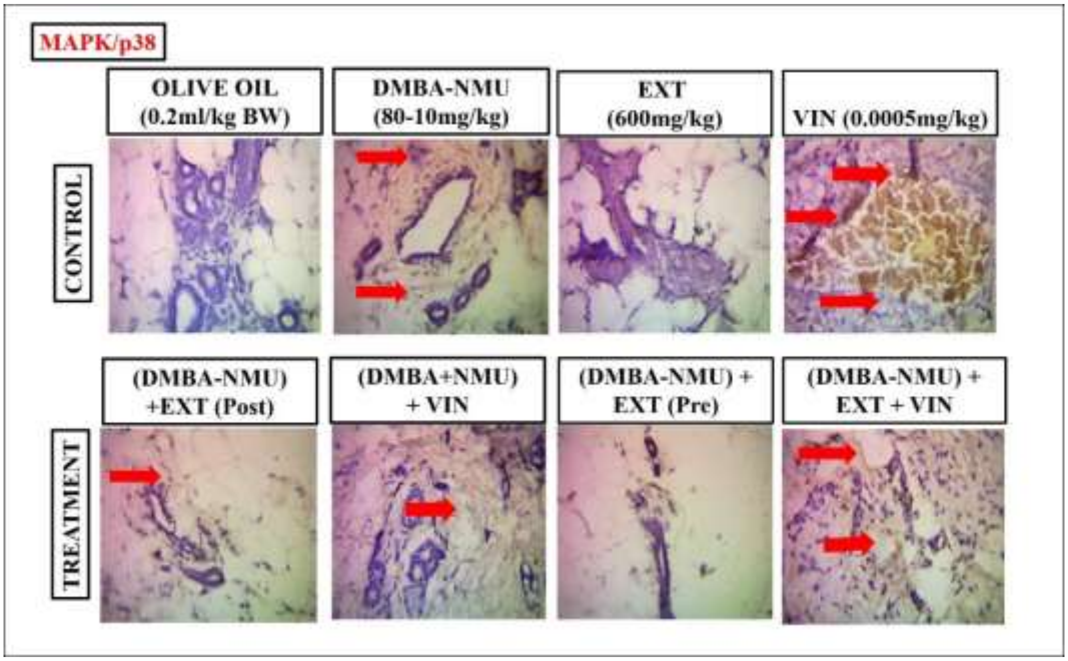

Supplementary Figure 8: Photomicrographs of the effects of *PoEE* on the Expression of MAPK(p38) protein in breast tissues of DMBA-NMU induced BC in Female SD rats. Red arrows in the photomicrographs show the areas of expression.

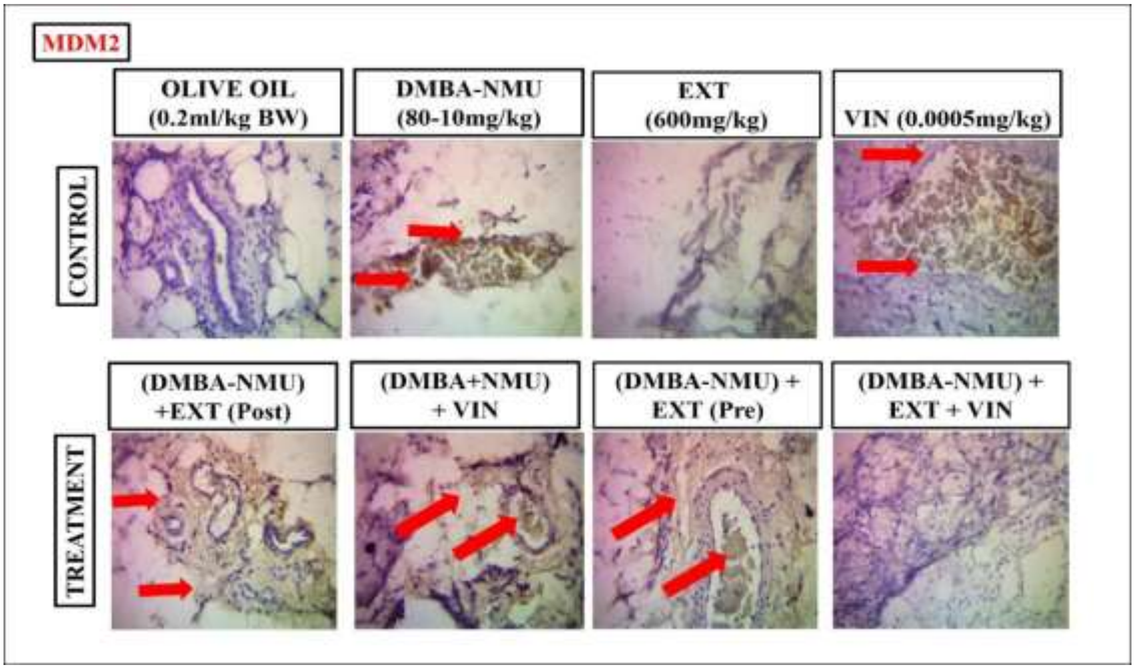

Supplementary Figure 9: Photomicrographs of the effects of *PoEE* on the Expression of MDM2 protein in breast tissues of DMBA-NMU induced BC in Female SD rats. Red arrows in the photomicrographs show the areas of expression.

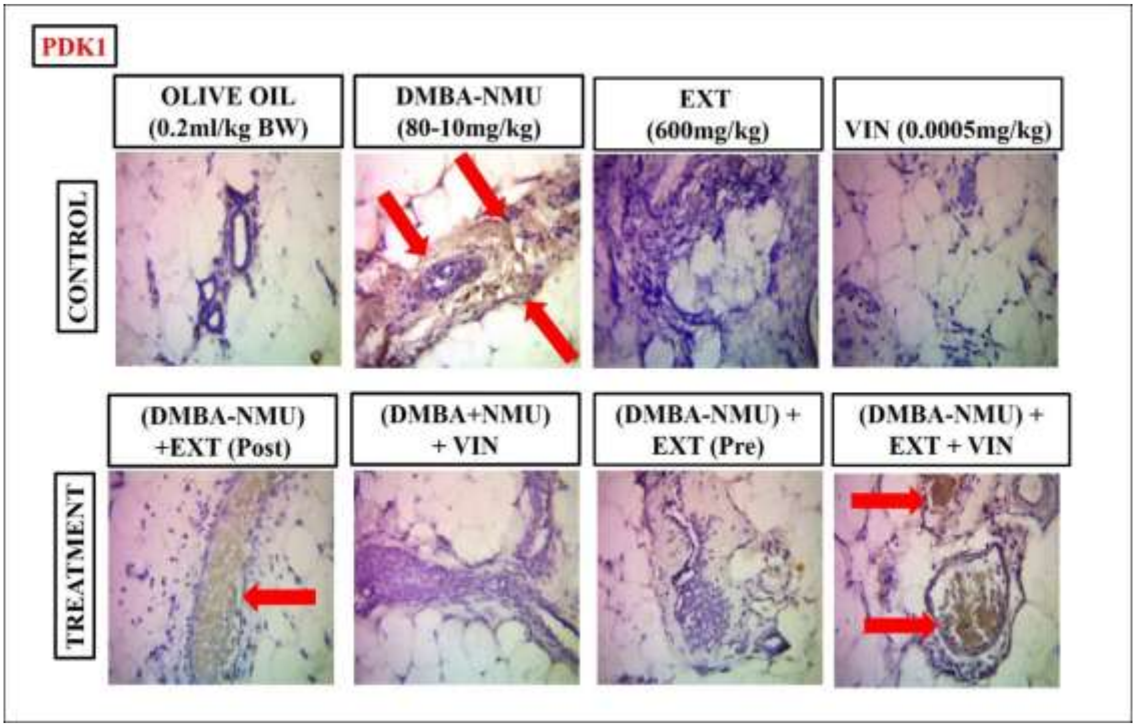

Supplementary Figure 10: Photomicrographs of the effects of *PoEE* on the Expression of PDK1 protein in breast tissues of DMBA-NMU induced BC in Female SD rats. Red arrows in the photomicrographs show the areas of expression.

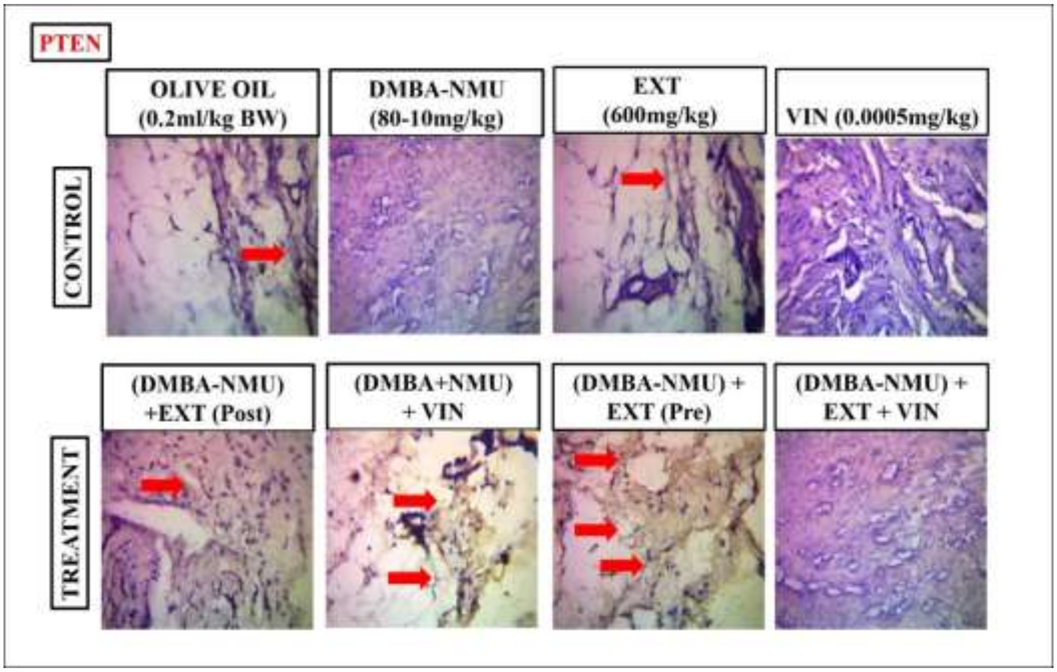

Supplementary Figure 11: Photomicrographs of the effects of *PoEE* on the Expression of PTEN protein in breast tissues of DMBA-NMU induced BC in Female SD rats. Red arrows in the photomicrographs show the areas of expression.

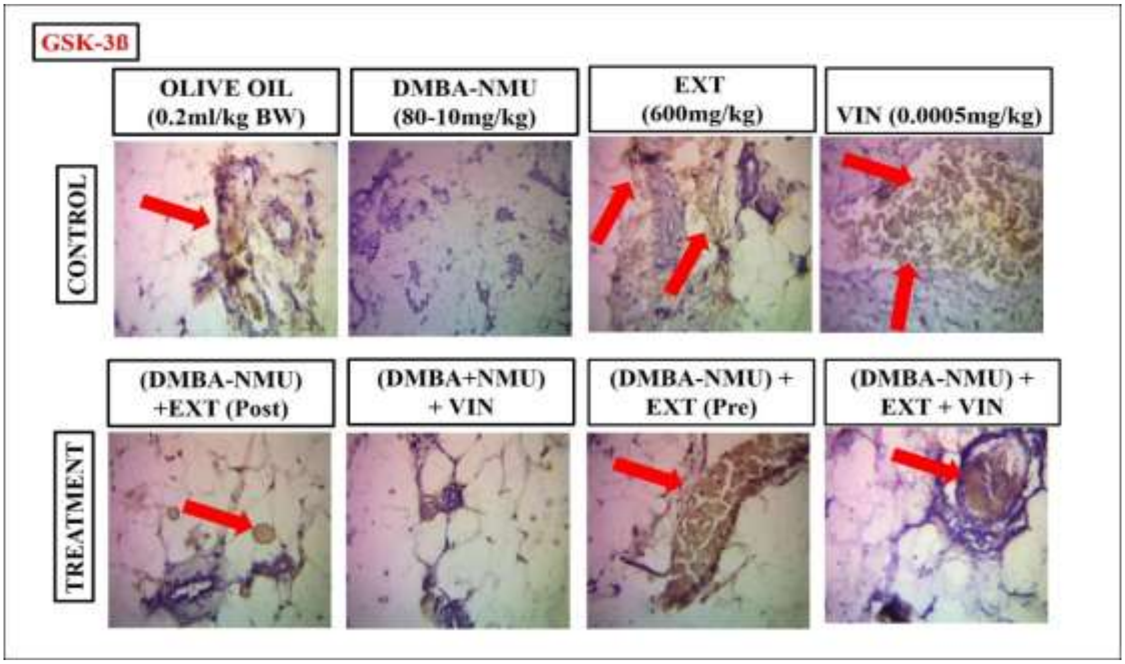

Supplementary Figure 12: Photomicrographs of the effects of *PoEE* on the Expression of GSK3β protein in breast tissues of DMBA-NMU induced BC in Female SD rats. Red arrows in the photomicrographs show the areas of expression.

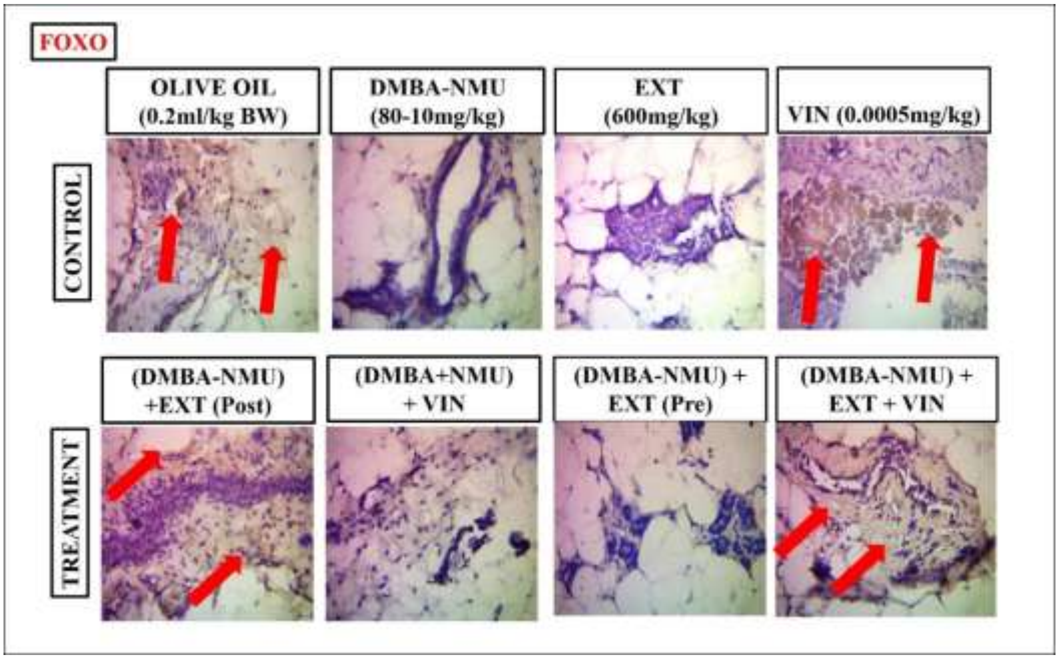

Supplementary Figure 13: Photomicrographs of the effects of *PoEE* on the Expression of FOXO protein in breast tissues of DMBA-NMU induced BC in Female SD rats. Red arrows in the photomicrographs show the areas of expression.

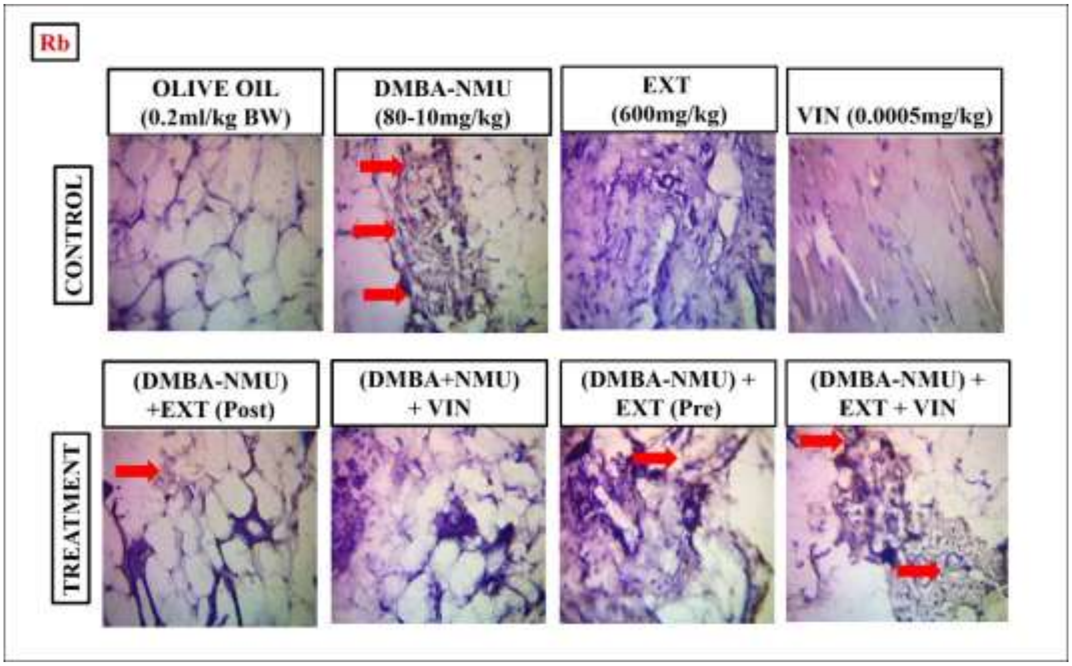

Supplementary Figure 14: Photomicrographs of the effects of *PoEE* on the Expression of Rb protein in breast tissues of DMBA-NMU induced BC in Female SD rats. Red arrows in the photomicrographs show the areas of expression.

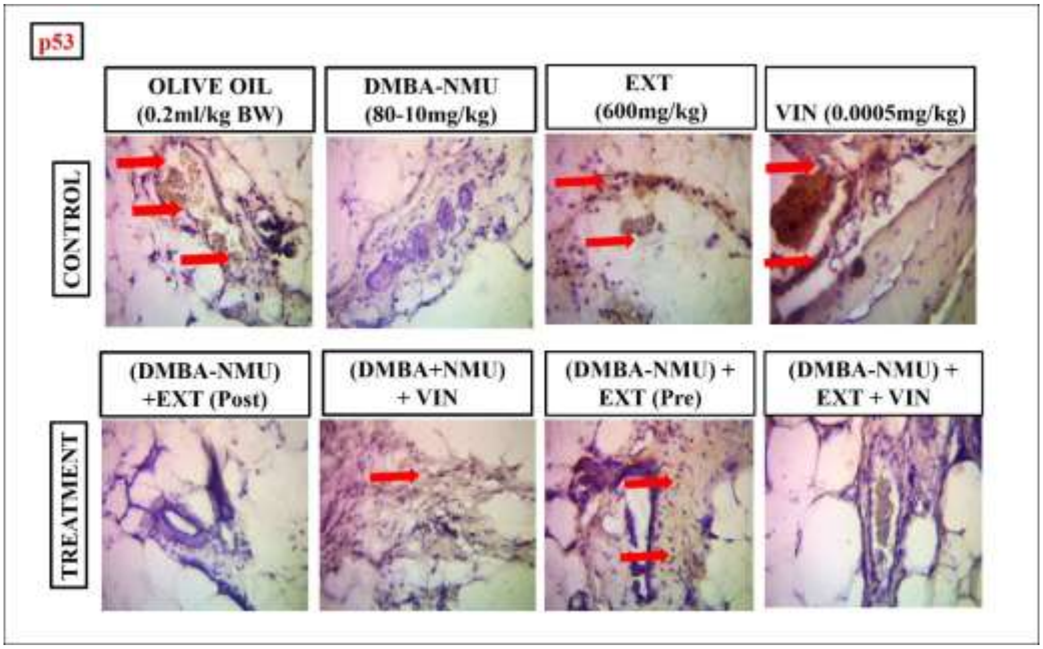

Supplementary Figure 15: Photomicrographs of the effects of *PoEE* on the Expression of p53 protein in breast tissues of DMBA-NMU induced BC in Female SD rats. Red arrows in the photomicrographs show the areas of expression.

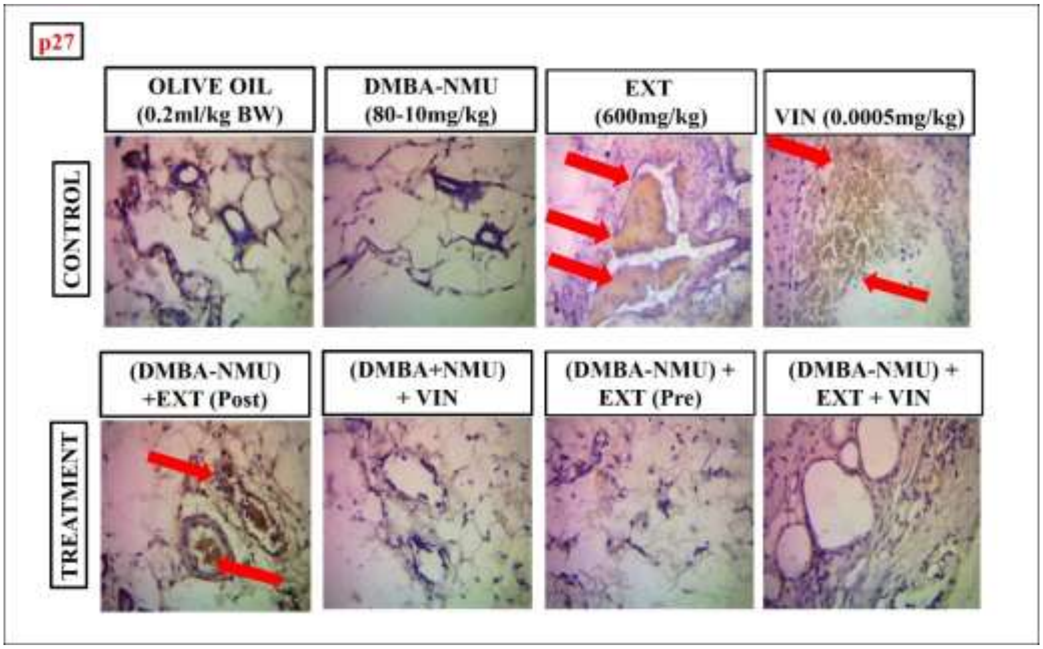

Supplementary Figure 16: Photomicrographs of the effects of *PoEE* on the Expression of p27 protein in breast tissues of DMBA-NMU induced BC in Female SD rats. Red arrows in the photomicrographs show the areas of expression.

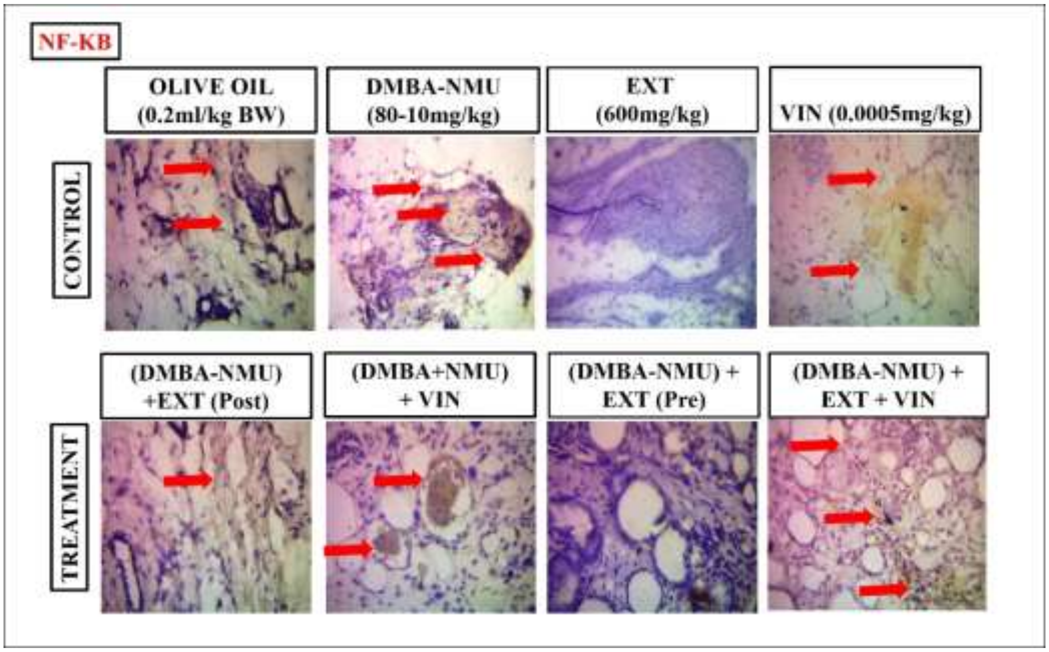

Supplementary Figure 17: Photomicrographs of the effects of *PoEE* on the Expression of NFkB protein in breast tissues of DMBA-NMU induced BC in Female SD rats. Red arrows in the photomicrographs show the areas of expression.

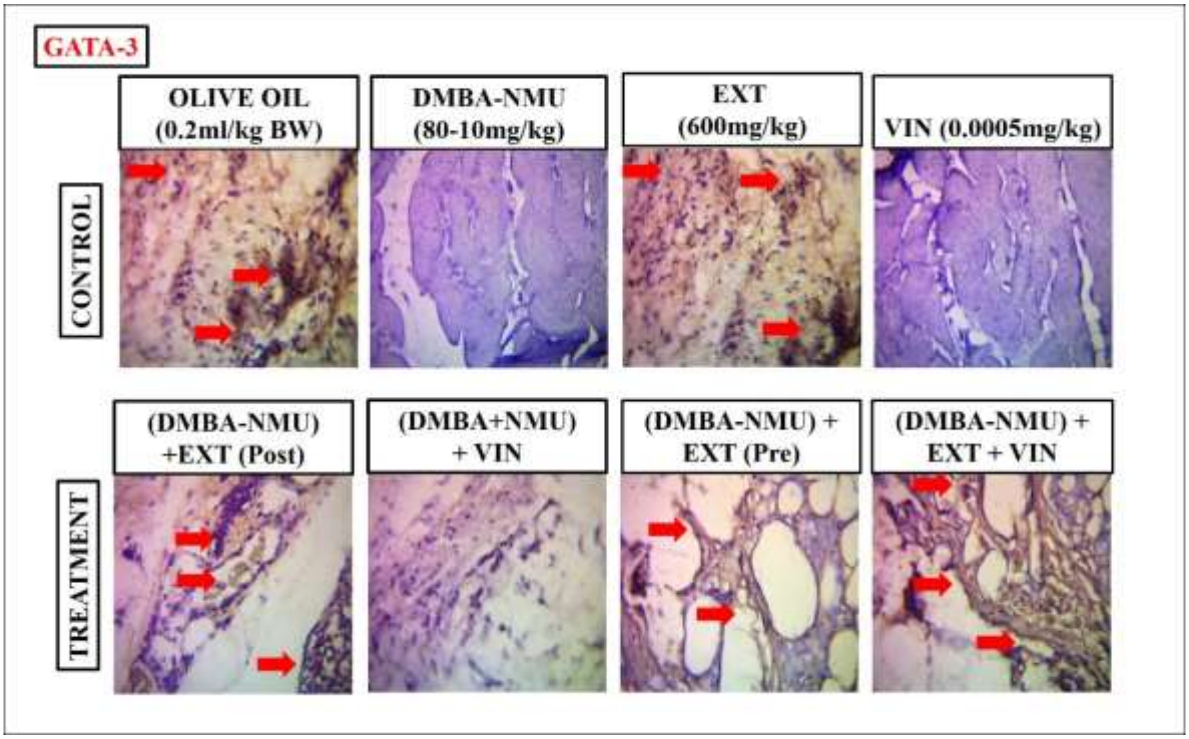

Supplementary Figure 18: Photomicrographs of the effects of *PoEE* on the Expression of GATA-3 protein in breast tissues of DMBA-NMU induced BC in Female SD rats. Red arrows in the photomicrographs show the areas of expression.

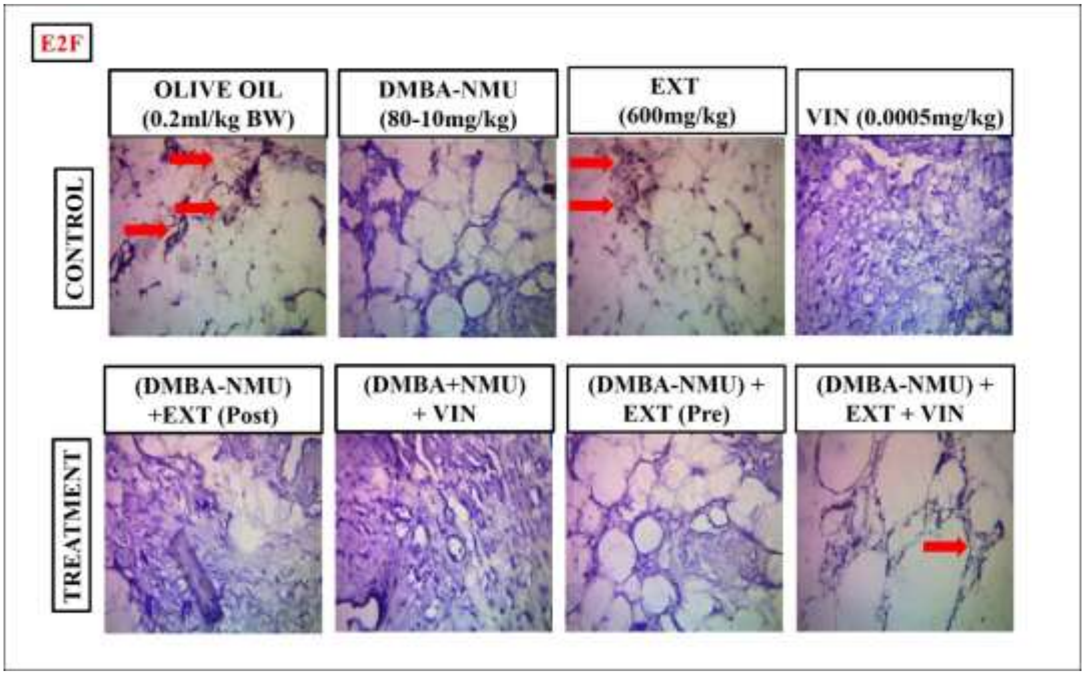

Supplementary Figure 19: Photomicrographs of the effects of *PoEE* on the Expression of E2F protein in breast tissues of DMBA-NMU induced BC in Female SD rats. Red arrows in the photomicrographs show the areas of expression.

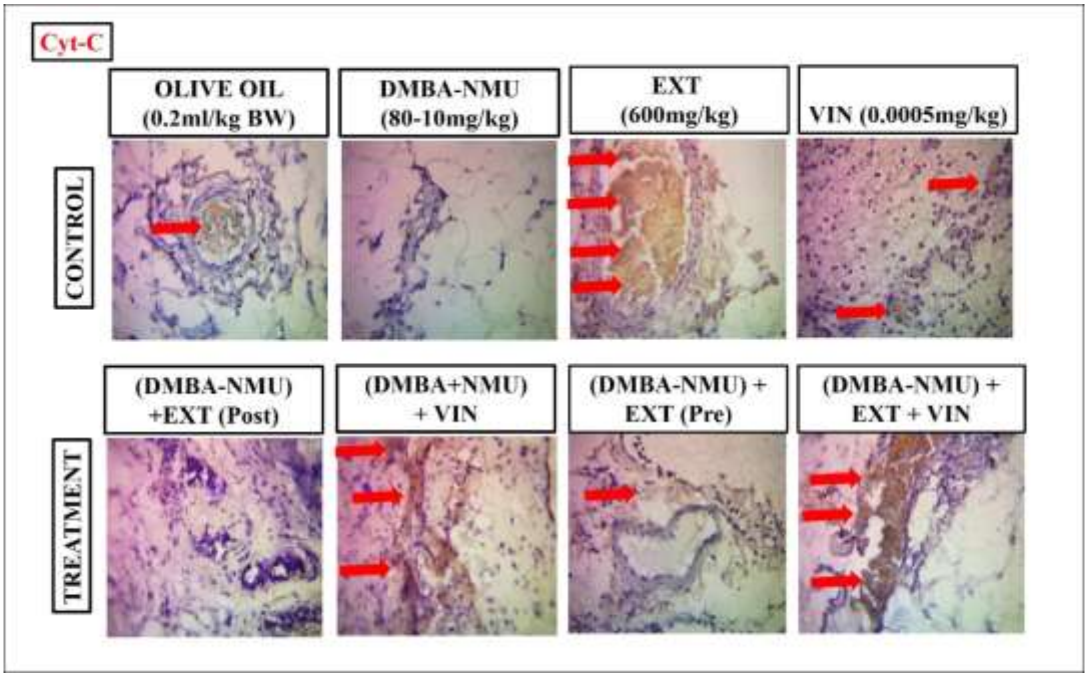

Supplementary Figure 20: Photomicrographs of the effects of *PoEE* on the Expression of Cyt-C protein in breast tissues of DMBA-NMU induced BC in Female SD rats. Red arrows in the photomicrographs show the areas of expression.

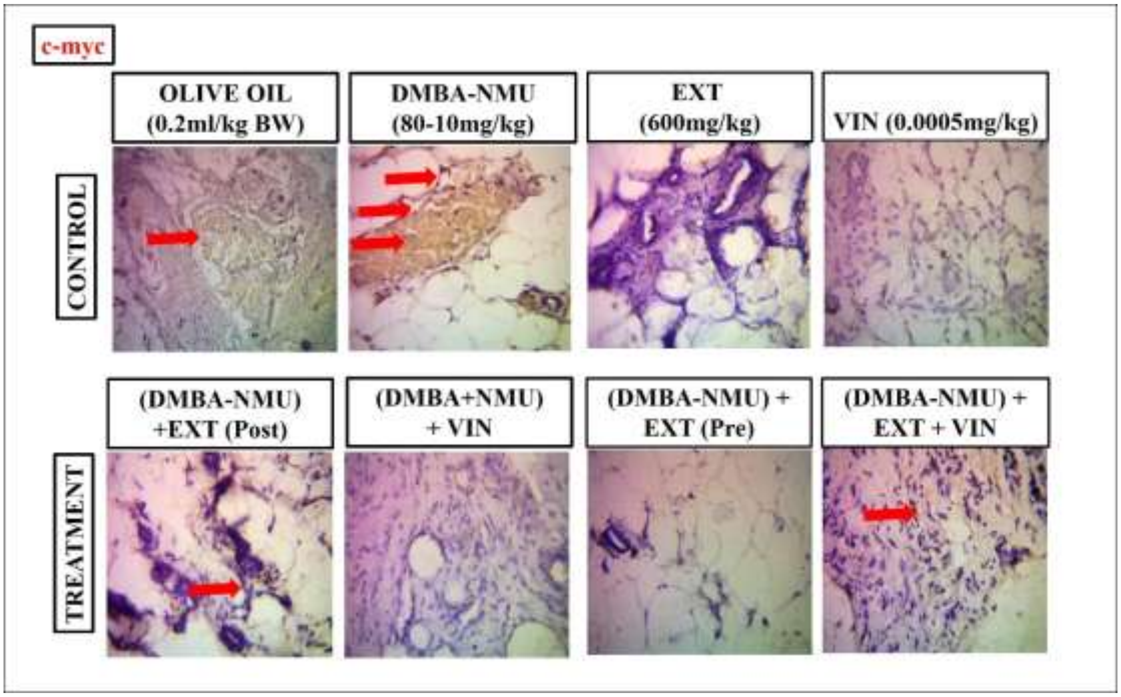

Supplementary Figure 21: Photomicrographs of the effects of *PoEE* on the Expression of c-myc protein in breast tissues of DMBA-NMU induced BC in Female SD rats. Red arrows in the photomicrographs show the areas of expression.

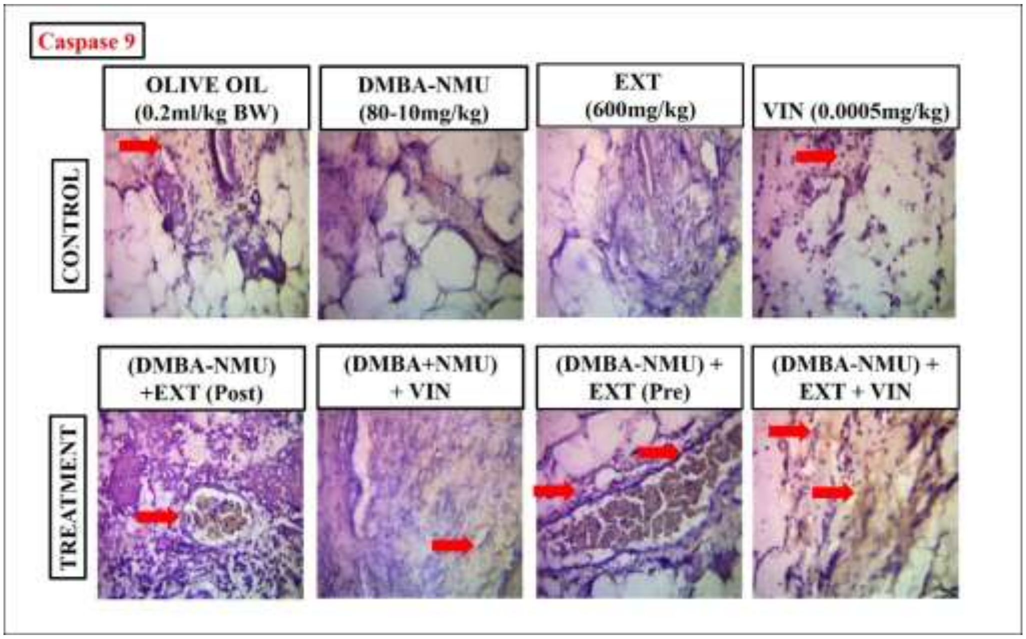

Supplementary Figure 22: Photomicrographs of the effects of *PoEE* on the Expression of Caspase 9 protein in breast tissues of DMBA-NMU induced BC in Female SD rats. Red arrows in the photomicrographs show the areas of expression.

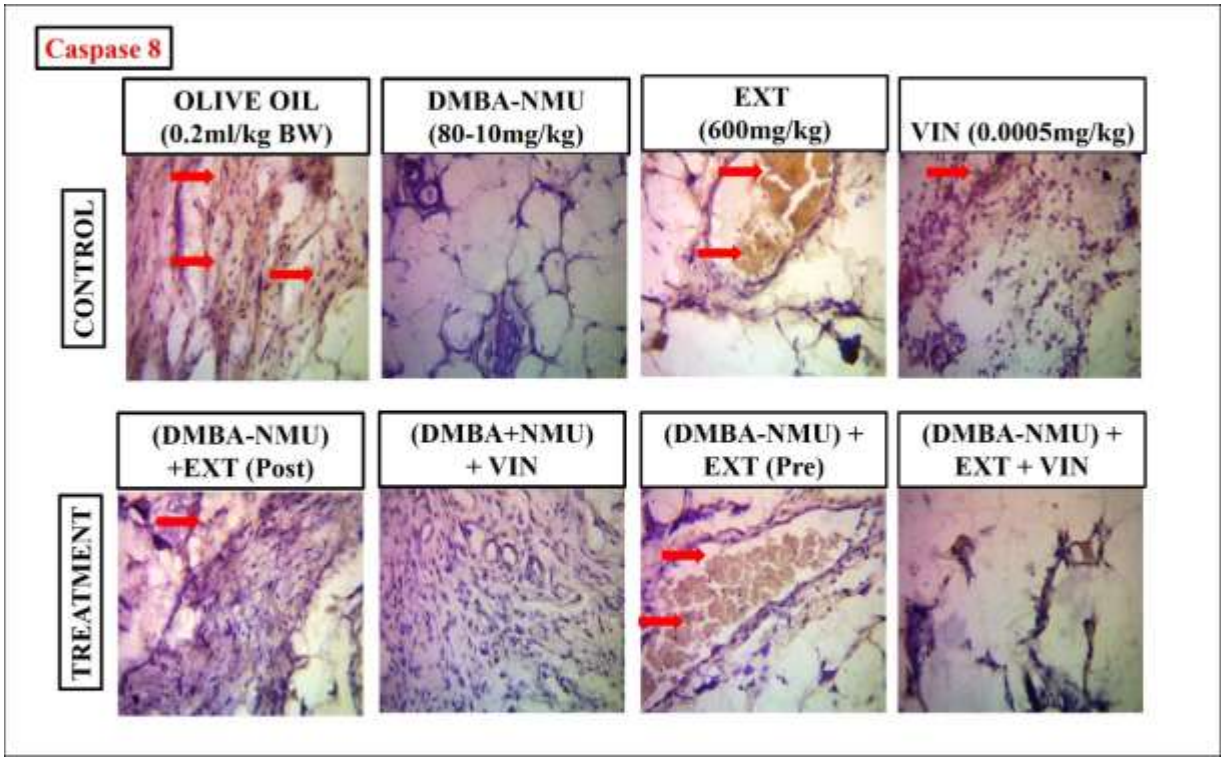

Supplementary Figure 23: Photomicrographs of the effects of *PoEE* on the Expression of Caspase 8 protein in breast tissues of DMBA-NMU induced BC in Female SD rats. Red arrows in the photomicrographs show the areas of expression.

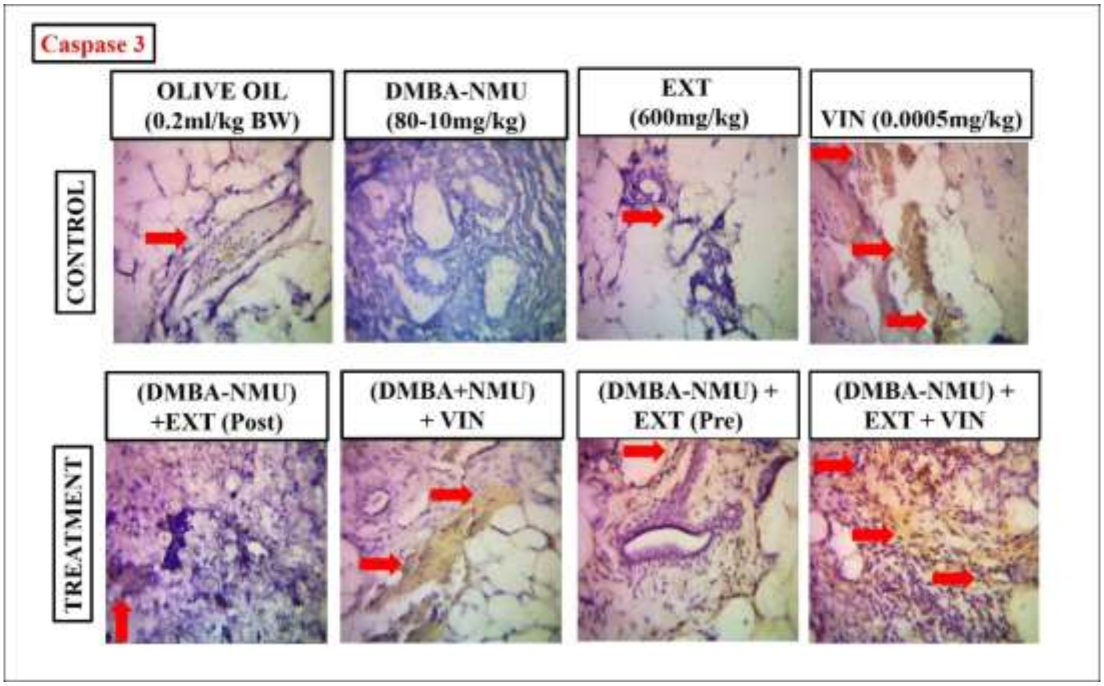

Supplementary Figure 24: Photomicrographs of the effects of *PoEE* on the Expression of Caspase 3 protein in breast tissues of DMBA-NMU induced BC in Female SD rats. Red arrows in the photomicrographs show the areas of expression.

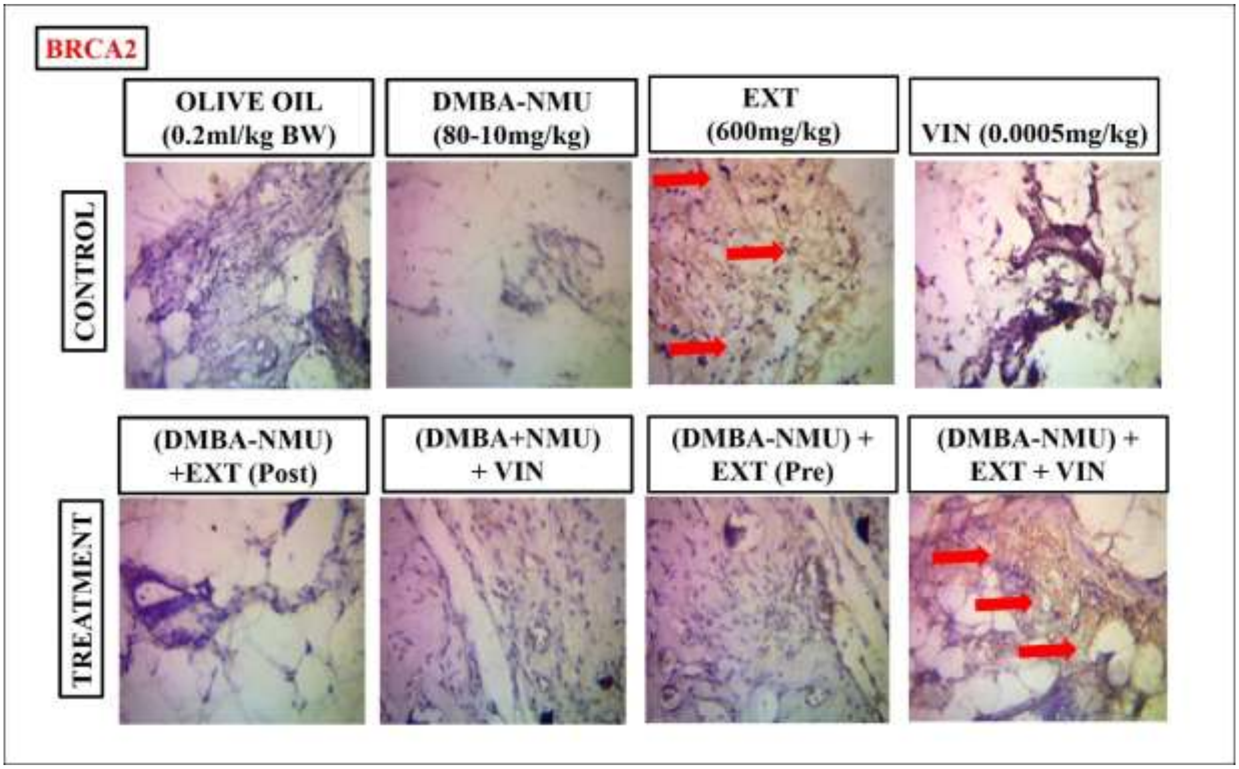

Supplementary Figure 25: Photomicrographs of the effects of *PoEE* on the Expression of BRCA2 protein in breast tissues of DMBA-NMU induced BC in Female SD rats. Red arrows in the photomicrographs show the areas of expression.

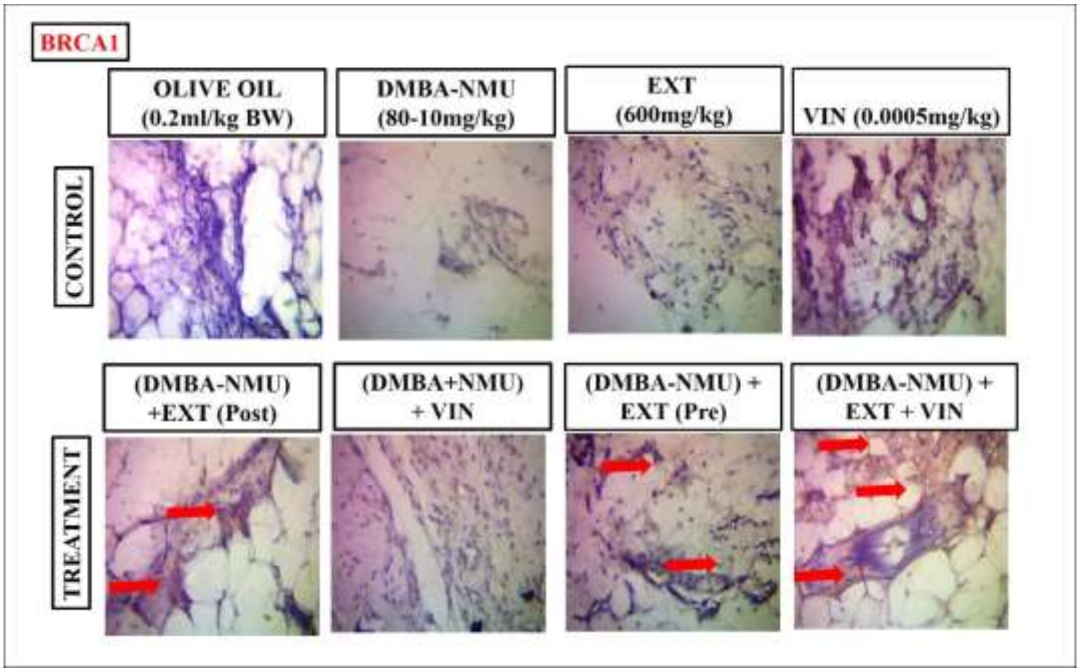

Supplementary Figure 26: Photomicrographs of the effects of *PoEE* on the Expression of BRCA1 protein in breast tissues of DMBA-NMU induced BC in Female SD rats. Red arrows in the photomicrographs show the areas of expression.

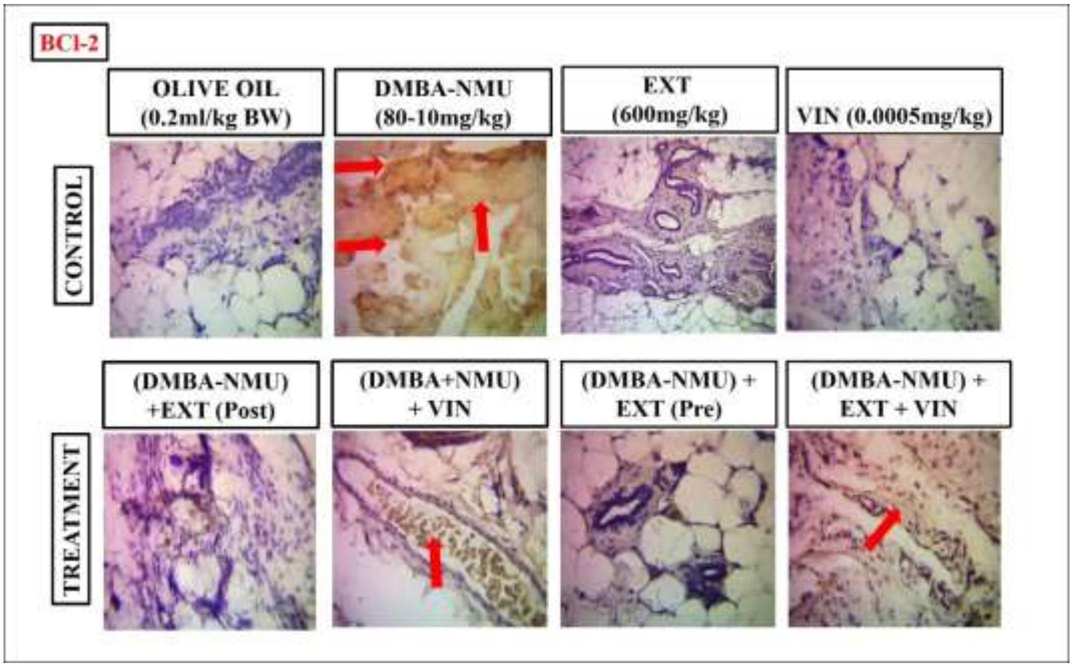

Supplementary Figure 27: Photomicrographs of the effects of *PoEE* on the Expression of BCL-2 protein in breast tissues of DMBA-NMU induced BC in Female SD rats. Red arrows in the photomicrographs show the areas of expression.

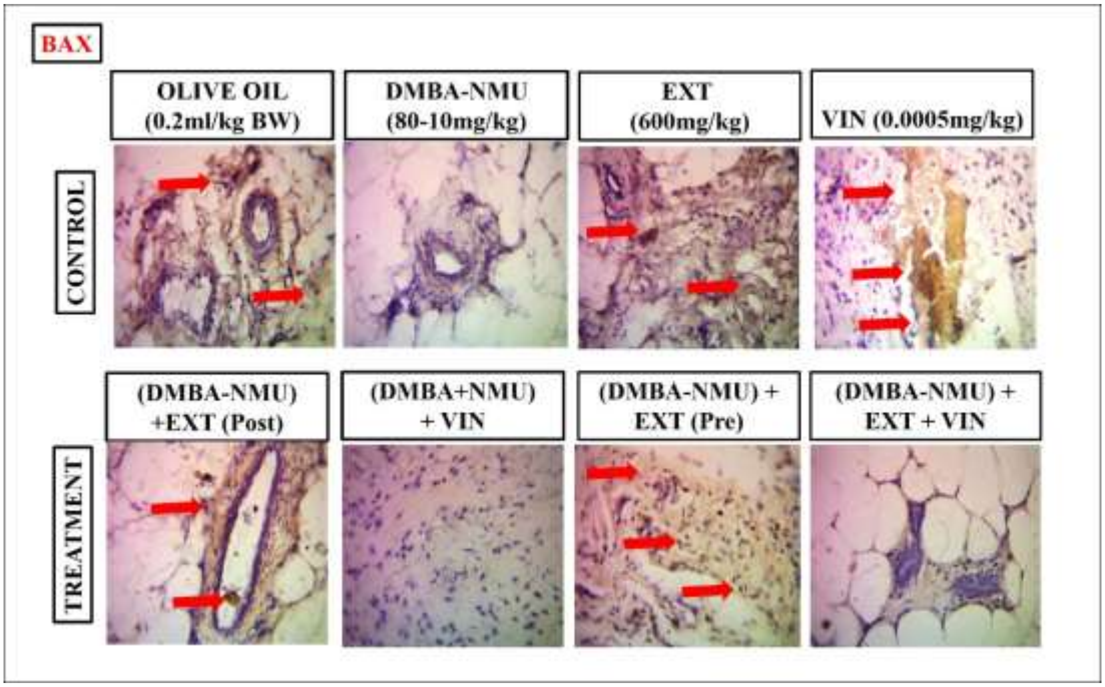

Supplementary Figure 28: Photomicrographs of the effects of *PoEE* on the Expression of BAX protein in breast tissues of DMBA-NMU induced BC in Female SD rats. Red arrows in the photomicrographs show the areas of expression.

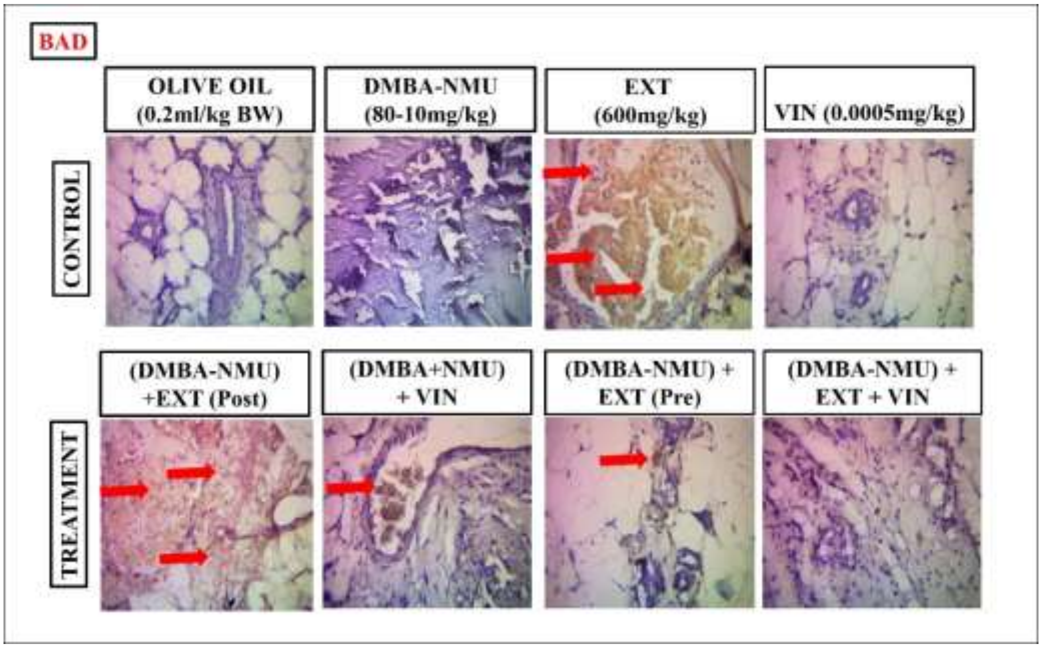

Supplementary Figure 29: Photomicrographs of the effects of *PoEE* on the Expression of BAD protein in breast tissues of DMBA-NMU induced BC in Female SD rats. Red arrows in the photomicrographs show the areas of expression.

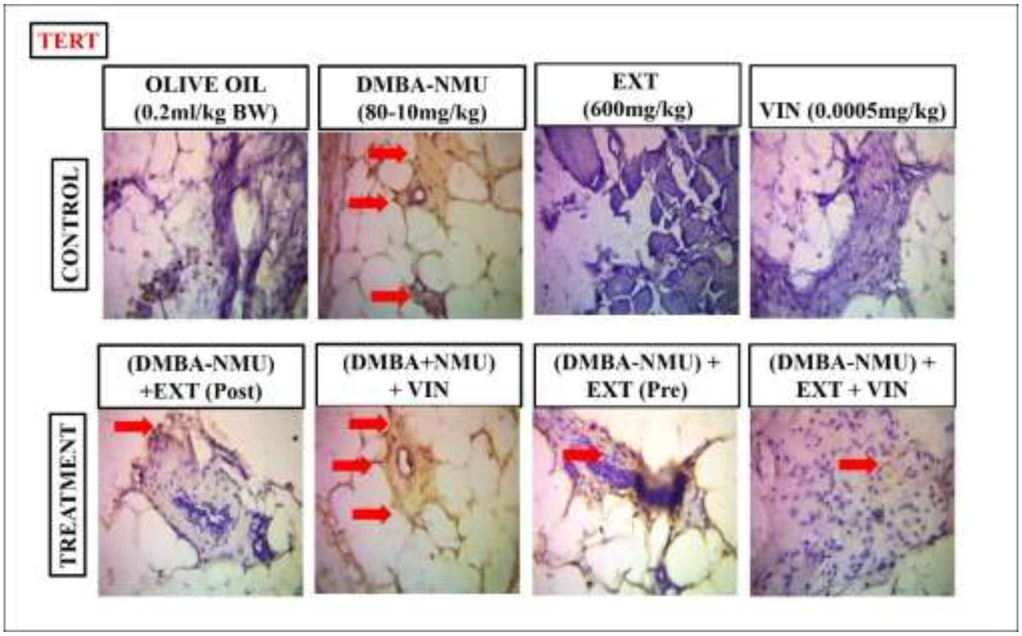

Supplementary Figure 30: Photomicrographs of the effects of *PoEE* on the Expression of TERT protein in breast tissues of DMBA-NMU induced BC in Female SD rats. Red arrows in the photomicrographs show the areas of expression.
